# A study on the monitoring of LNAPL migration using ERT

**DOI:** 10.1371/journal.pone.0315624

**Published:** 2025-01-24

**Authors:** Kui Suo, Mingdong Zhao, Yu Liu, Hongwei Liu, Menghan Jia

**Affiliations:** 1 North China University of Water Resources and Electric Power, Zhengzhou City, Henan Province, P.R. China; 2 Zhongsheng Environmental Science and technology Development Co., LTD, Xi’an, Shaanxi Province, P.R. China; Linköping University: Linkopings universitet, SWEDEN

## Abstract

This study employs electrical resistivity tomography (ERT) to experimentally investigate the migration characteristics of light non-aqueous phase liquids (LNAPL) under various groundwater conditions. Through cross-hole measurements and time-lapse inversion, the migration process of LNAPL under three scenarios—unsaturated conditions, constant groundwater levels, and declining water levels—was systematically analyzed. The results indicate that LNAPL migration behavior exhibits significant differences under different conditions. Under unsaturated conditions, the vertical migration rate of LNAPL gradually decreases over time, with an average rate of 1.06 cm/h, and is influenced by preferential migration pathways formed in coarse-grained regions. At constant water levels, the migration of LNAPL is significantly constrained by the groundwater level, spreading horizontally near the water table after reaching it, with an average rate of 0.51 cm/h. When the groundwater level declines, LNAPL migrates rapidly downward along preferential flow paths, with an average rate increasing to 1.45 cm/h. Miller Soil Box experiments further reveal the relationship between LNAPL content and electrical resistivity, showing that an increase in LNAPL can significantly alter soil resistivity, especially under low moisture conditions. Overall, this study confirms the monitoring advantages of ERT technology for LNAPL migration behavior under different conditions and provides important references for remediation strategies at contaminated sites.

## 1 Introduction

Since the early 20th century, petroleum has been widely used as a primary energy source, bringing economic benefits while also causing significant water pollution issues. Particularly, Light Non-Aqueous Phase Liquid (LNAPL), which is less dense than water, can seep downwards from the surface and spread continuously, primarily migrating vertically after a leak, causing severe contamination to the groundwater environment. The migration mechanism of LNAPL becomes extremely complex in the unsaturated-saturated zone due to the immiscibility of oil and water. Moreover, the remediation of contaminated sites is also quite challenging [1]. Therefore, studying the migration characteristics of LNAPL under different groundwater storage conditions is of great significance for understanding the migration behavior of LNAPL and provides important reference value for guiding the actual remediation of contaminated sites. Consequently, numerous scholars have conducted extensive research in the field of LNAPL contamination. Early on, P.J. Van Geel and J.F. Sykes et al., in 1994, simulated the migration process of LNAPL in variably saturated sandy media through two-dimensional multiphase flow experiments, revealing the impact of trapped air on LNAPL migration and maximum saturation, as well as the failure of the static equilibrium capillary pressure-saturation relationship at the LNAPL front [2]. In recent years, Su Kong Ngien and Norhan Abd. Rahman pointed out in their 2012 research that the flow velocity of LNAPL in aggregated soil is significantly faster than that in single-pore soil [3]. N. A. Rahman and L. K. Foong, in 2018, used digital image processing technology and observed that an increase in soil moisture content increases capillary pressure, thereby accelerating the downward migration of LNAPL [4, 5]. Praseeja A.V. and Sajikumar N., in their 2022 research, discussed the impact of natural fibers on the migration of LNAPL in unsaturated soil [6, 7]. When soil is contaminated by LNAPL, its basic physical properties will change [8]. Pan, Y’s team, in 2021, measured and analyzed the migration process of petroleum in heterogeneous soil using high-density resistivity methods and verified the resistivity measurement results through physical and chemical property analysis [9–11]. Meng Jian and Dong Yanhui, in 2022, characterized LNAPL pollution plumes using electrical resistivity tomography (ERT), employing optimized arrays to improve the resolution and cost estimation strategies for contaminated site investigations [12, 13]. Jonás García-Rincón and Evangelos Gatsios, in 2020, effectively improved the high-resolution characterization of LNAPL migration using laser-induced fluorescence (LIF) logging technology [14]. C.Kechavarzi, in 2023, described the distribution and transport of LNAPL in a two-dimensional indoor model by inserting resistivity probes to measure water and LNAPL saturation, concluding that the initial transport of LNAPL is faster and slows over time [15]. Li ZhiPing and Liu Yu et al., in 2023, used time-series electrical resistivity tomography to study the migration mechanism of LNAPL under different conditions, proving the significant value and effectiveness of ERT as a geophysical method for monitoring and characterizing the migration characteristics of LNAPL [16, 17].

Clearly, geophysical techniques have been widely applied in the field of LNAPL contamination research, with ERT experiencing rapid development due to its advantages of being fast and non-destructive [18]. There has been in-depth research on the impact of LNAPL on the physical properties of the oil phase, the capillary fringe, and the particle size and uniformity of the experimental medium. However, there is relatively little research on the application of ERT with cross-borehole measurements for studying the migration of LNAPL under different groundwater storage conditions.

Therefore, to investigate the full process of LNAPL migration under different groundwater storage conditions, this paper employs ERT cross-hole measurements and conducts time-lapse inversion processing to summarize the patterns of resistivity changes during the LNAPL migration process. By integrating camera monitoring, the relationship between LNAPL migration rate and time is derived through functional fitting and differentiation, discussing the migration characteristics of LNAPL under various groundwater storage conditions.

## 2 Materials and methods

### 2.1 Experimental setup and materials

To ensure that LNAPL does not affect subsequent experiments, two identical sets of apparatus and materials were used in this study. The experimental setup includes two acrylic cylindrical tubes (with a diameter of 23.5 cm, height of 60 cm, and wall thickness of 0.5 cm), a peristaltic pump, a loading platform, a Markov flask, polyurethane (PU) tubing, and a DUK-2B High-Density Electrical Measurement System (referred to as DUK-2B). To make the experimental results more representative of real-world conditions, sand samples were randomly selected from the south bank of the Yellow River in the Beijiao Huayuankou area, Huiji District, Zhengzhou City, Henan Province. The use of natural samples aims to better understand the behavior of LNAPL in natural environments. After decontamination, natural air-drying, re-decontamination, crushing, and sieving processes, the sand sample parameters were obtained through geotechnical tests (**Table 1**). The particle size of the sand sample ranges from 0.005 mm to 2 mm, with an average diameter of 0.205 mm. It is an extremely non-uniform medium, characterized by a non-uniformity coefficient greater than 10 and a curvature coefficient exceeding 3. This suggests that the sand sample may lack intermediate-sized particles, classifying it as a discontinuous gradation.

**Table 1:** Particle size distribution table of sand samples.

Particle Diameter (mm)	Percentage Content (%)	Average Particle Diameter (mm)	Specific Gravity	Uniformity Coefficient	Curvature Coefficient	Designation
2–0.5	17.5	0.205	2.68	30.87	4.78	Fine Sand
0.5–0.25	26.2					
0.25–0.075	38.1					
0.75–0.005	12.9					
<0.005	5.3					

The LNAPL experimental reagent used in this study was diesel oil, which was dyed with a small amount of Sudan Red III. The diesel oil was obtained from the 16th PetroChina gas station in Zhengzhou. The physical properties of the diesel oil are detailed in **Table 2**. The purpose of staining the LNAPL reagent with dye was to enhance its visibility for observation. The dye content is minimal and does not affect the physical or chemical properties of the diesel oil. Therefore, it is not expected to have any impact on the results of this experiment.

**Table 2:** Basic physical properties of diesel.

Diesel Type	Density (g/ml)	Boiling Point (°C)	Surface Tension at 20° C (dyne/cm)	Dynamic Viscosity at 20°C (cst)	Flash Point (°C)
0#	0.829	180–370	27.8	2.54	220

### 2.2 Experimental design

**Fig 1:**
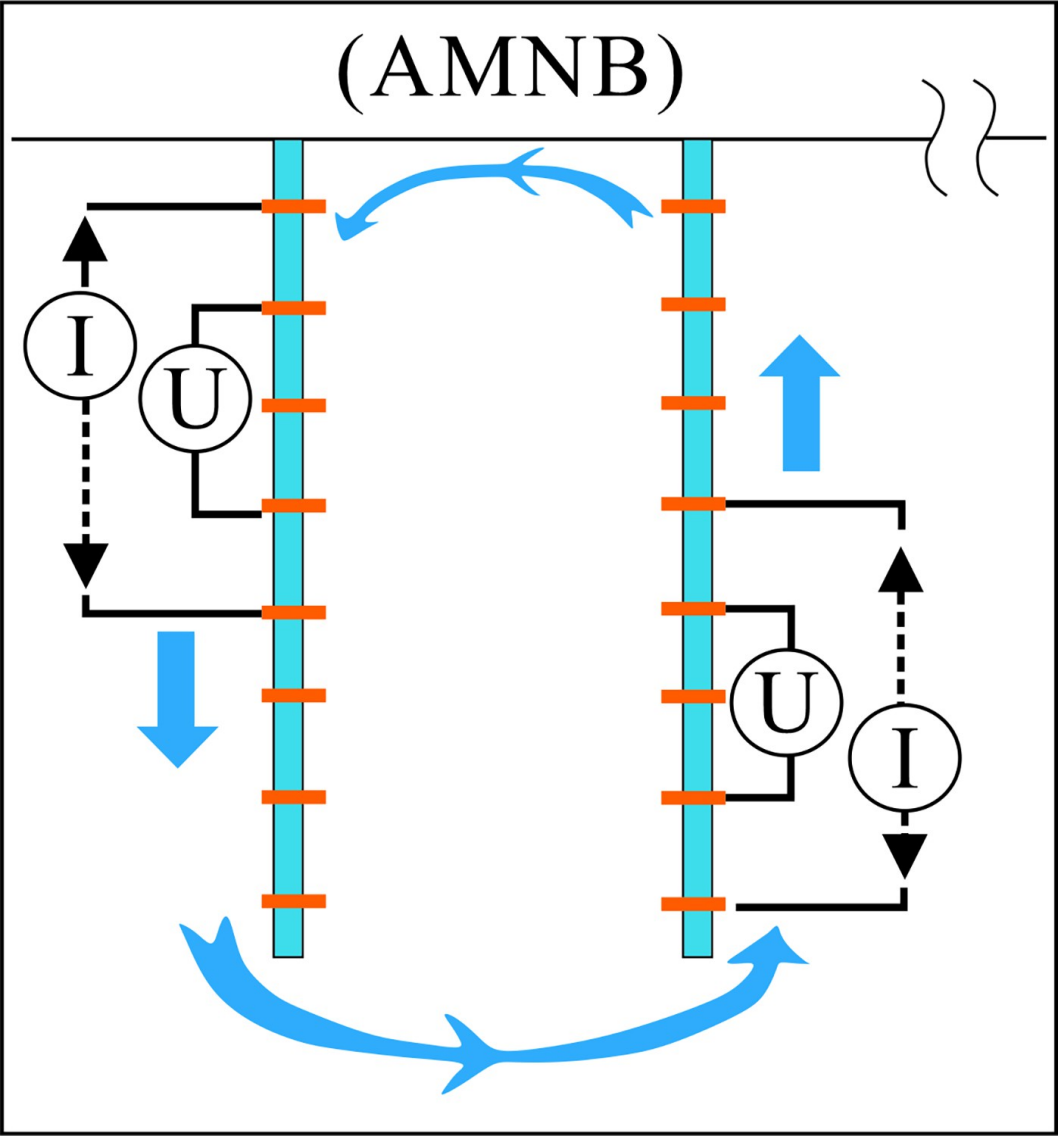
Schematic diagram of the Wenner cross-hole measurement principle.

To explore the differences in infiltration mode and rate of LNAPL under different groundwater existence states, we conducted a study. We examined the migration process of LNAPL under three conditions: unsaturated conditions, constant groundwater level, and declining groundwater level. By observing the LNAPL infiltration front and monitoring resistivity data, combined with the ERT cross-hole measurement method (**Fig 1**), we were able to determine the vertical migration patterns of LNAPL.

Additionally, we measured the electrical conductivity of LNAPL at various concentrations using the Miller Soil Box to investigate changes in electrical resistivity upon LNAPL entry into the medium. It is worth noting that electrical conductivity and electrical resistivity are reciprocal values, and we utilized Formula (1) to obtain the corresponding values of electrical resistivity.


$$\rho =\frac{1}{\sigma }$$ (1)

In this context, ρ represents electrical resistivity, and *σ* represents electrical conductivity.

#### 2.2.1 Migration under unsaturated condition

**Fig 2:**
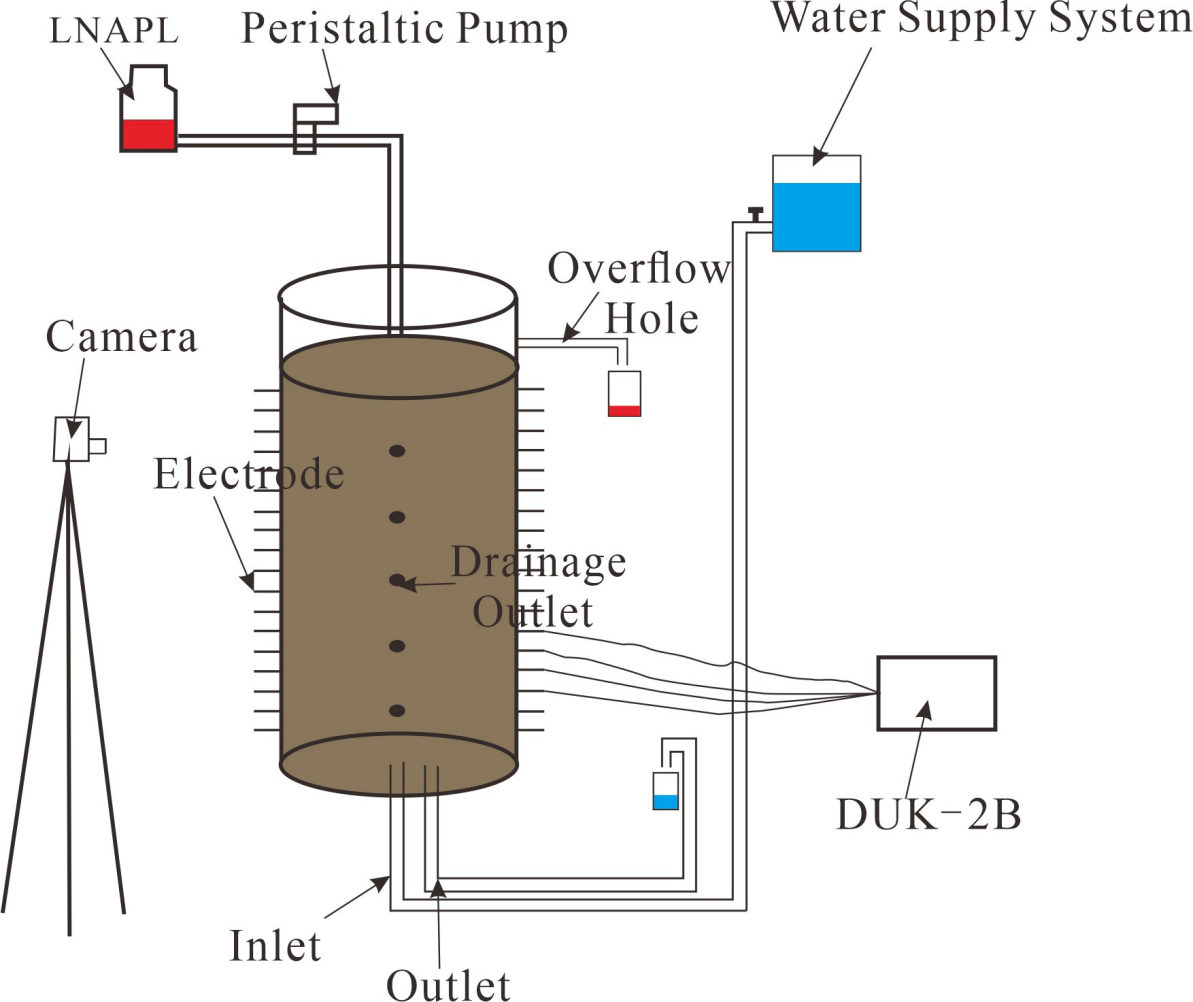
Experimental setup.

The experiment was conducted using the apparatus depicted in **Fig 2**. The sand within the setup was in an unsaturated state, and the oil surface completely covered the top sand layer of the acrylic cylinder. A constant pressure was maintained to facilitate natural infiltration. The experiment data were recorded using a high-definition camera and the DUK-2B. Recording was stopped when LNAPL appeared in the bottom drainage pipe.

#### 2.2.2 Migration under conditions of constant groundwater table and declining groundwater table

During the experiment, the sand body is first fully saturated. A water stop clamp at an appropriate height is opened to create a certain water level within the soil column, simulating a condition with a constant groundwater table. LNAPL is then introduced from the top using a peristaltic pump, with the oil surface completely covering the sand layer at the top of the acrylic cylinder. The entire migration process of LNAPL is monitored using a DUK-2B and a high-definition camera. After the experiment with a constant groundwater table is concluded, the bottom drainage hole is opened to lower the groundwater table, and the entire process is recorded using the DUK-2B and a high-definition camera.

#### 2.2.3 Miller soil box experiment

**Fig 3:**
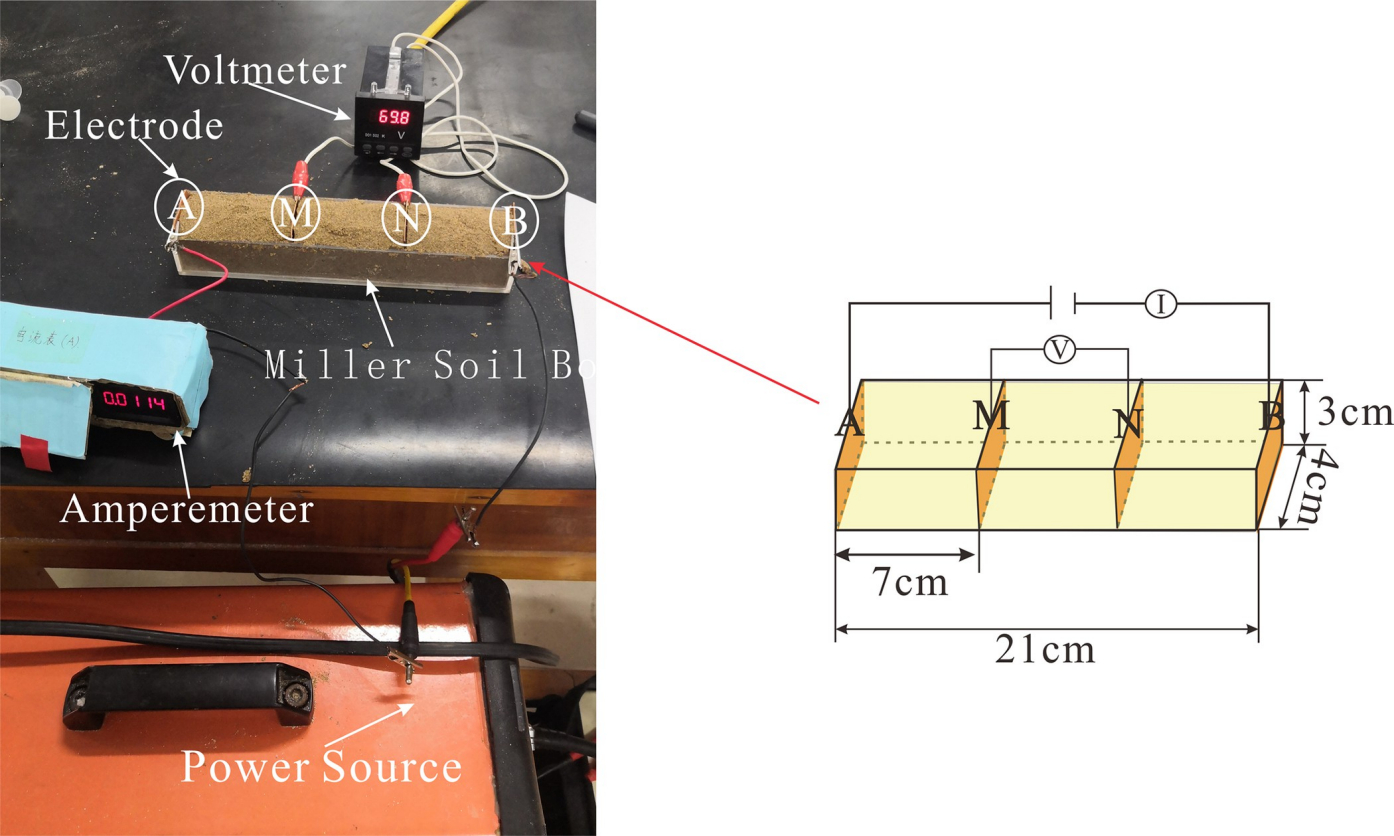
Schematic diagram of the miller soil box.

The Miller Soil Box, made of non-conductive materials such as acrylic panels, is depicted in **Fig 3**. It consists of copper sheet electrodes A, M, N, and B, each measuring 4cm × 4cm. The distance between electrodes AB is 21 cm, and the distances AM, MN, and NB are all 7cm [19]. The experimental sand used is the same as that in the migration experiments under different groundwater storage conditions. Since the relative content of water and oil in the medium changes under different groundwater storage conditions, sand samples with LNAPL contents of 0%, 3%, 5%, 8%, 10%, 15%, 20%, and 25% were first prepared and placed into the Miller Soil Box for measurement. Then, corresponding amounts of water were added to each sand sample with different LNAPL contents, with water contents being 0%, 3%, 5%, 8%, 10%, 15%, and 20%. To ensure uniformity, the sand is gently pressed and compacted during the filling process. Subsequently, individual measurements are taken by inserting electrodes, and the data is recorded. During measurements, the positive terminal of the power supply is connected to electrode A, the negative terminal is connected to electrode B, and an ammeter is placed in between. Electrodes M and N are connected to a voltmeter.

### 2.3 Experimental steps

#### 2.3.1. Preparation for the experiment

The water supply and LNAPL delivery devices are positioned higher than the acrylic cylinder apparatus. To prevent sand from leaking out of the holes, permeable gauze is placed at all inlets and outlets. The treated sand samples are evenly spread in the acrylic cylinder apparatus, and every 1–2 cm of sand is gently compacted with a tamper to ensure the formation of a sand layer that simulates a uniform medium. The final filling height is 60 cm, with the top of the sand layer kept flat. The side wall of the apparatus is equipped with five circular holes, each with a diameter of 1 cm, for controlling the groundwater water table. There are 18 symmetrically positioned electrode holes on both sides of the apparatus, used for arranging electrodes. Each measurement line is 54 cm long, with electrode spacing at 3 cm, and measurements are taken using the Wenner configuration.

#### 2.3.2. Saturation adjustment

In order to simulate a fully saturated state of the medium, water is injected into the acrylic cylindrical setup from the bottom using the water supply unit. The water level is allowed to rise to the surface of the sand layer, forming a water film. Once this occurs, the water stopper is closed. After standing for 48 hours, the bottom outlet is opened to allow water to drain naturally under gravity until no water flows out of the drainage pipe. This process is repeated three times to achieve a stable non-saturated state of the medium. To simulate a constant groundwater level, the water stopper at the drainage hole, located 23 cm from the top on the side wall, is opened.

#### 2.3.3. Injecting LNAPL

The experiments conducted at room temperature are divided into three groups: LNAPL migration under unsaturated conditions, LNAPL migration under a constant groundwater table, and LNAPL migration during declining groundwater table conditions. LNAPL is injected into the acrylic cylinder using a peristaltic pump, with the oil surface completely covering the sand layer at the top of the acrylic cylinder and maintaining a constant oil layer thickness. Excess LNAPL is drained through an overflow hole at the top, with a final addition of 1.5 L of LNAPL. In the experiment with a constant groundwater table, the water level is controlled at a distance of 23 cm from the top. After the experiment is concluded, the water clamp at the bottom outlet is opened to conduct the migration experiment of LNAPL during declining groundwater table conditions.

#### 2.3.4. Data acquisition

Experimental data were recorded using a high-definition camera and the DUK-2B high-density electrical resistivity measurement system, starting from the moment LNAPL came into contact with the sand layer. For the unsaturated experiments, a photo was taken every 10 s, which was slowed down to one photo every 3 min after the infiltration rate decreased. When the infiltration rate became imperceptible to the naked eye, a photo was taken every 3 h, and after 24 h, the interval was changed to one photo every 8 h. For the constant groundwater table experiments, a photo was taken every 10 s at the start, which was slowed down to one photo every 3 min after the rate decreased. After the oil-water contact, the interval was changed to one photo every 3 h, and after 24 h, the interval was changed to one photo every 8 h. The migration experiment of LNAPL during the declining groundwater table was conducted based on the constant groundwater table experiment, with a photo taken every 5 s at the start, which was slowed down to one photo every 1.5 h after the rate decreased. After the experiment began, the DUK-2B high-density electrical resistivity instrument continuously monitored for 2 h and then changed to monitoring every hour until the end of the experiment.

### 2.4 Ethic statement

This study did not involve human or animal subjects, nor did it involve the collection and use of sensitive data or personal privacy information. As such, ethical review and approval were not required for this research. We affirm that all research activities adhered to scientific integrity and academic ethics, ensuring the impartiality and transparency of the study.

## 3 Data analysis

### 3.1 Data processing

To enhance the understanding of LNAPL migration in the resistivity inversion plot, the resistivity data collected in this study underwent a time-shift inversion. The inversion method proposed by Schütze et al. (2002), known as the commercial inversion or ratio method, was employed, as it is suitable for small-contrast scenarios [20]. This method can eliminate the influence of absolute numerical values, making changes of different scales comparable, and allowing us to more clearly understand the relative changes between two values. The formula for this method is shown as Eq (1). The processed data were then plotted using Surfer software to create graphical representations that highlight the most significant variations for presentation.


$$\rho =\frac{\rho_{t}-\rho_{o}}{\rho_{o}}$$ (2)

In the formula, represents the background resistivity value, which refers to the resistivity value before the introduction of LNAPL. represents the resistivity value at time t after the introduction of LNAPL. By utilizing the ruler placed inside the apparatus, along with measurements and high-density electrical data, it is possible to obtain data on the depth of infiltration.

**Fig 4:**
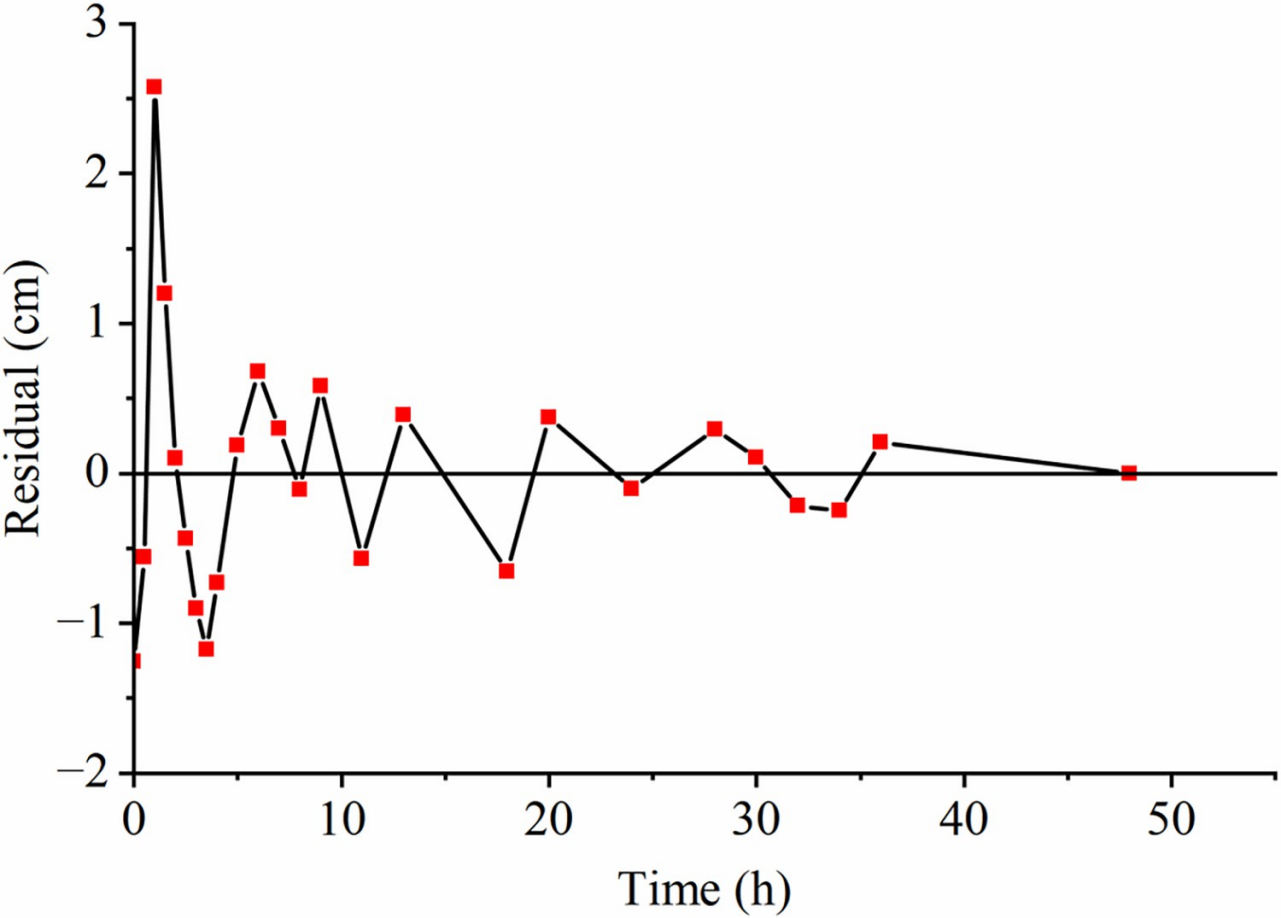
Residual distribution.

**Fig 5:**
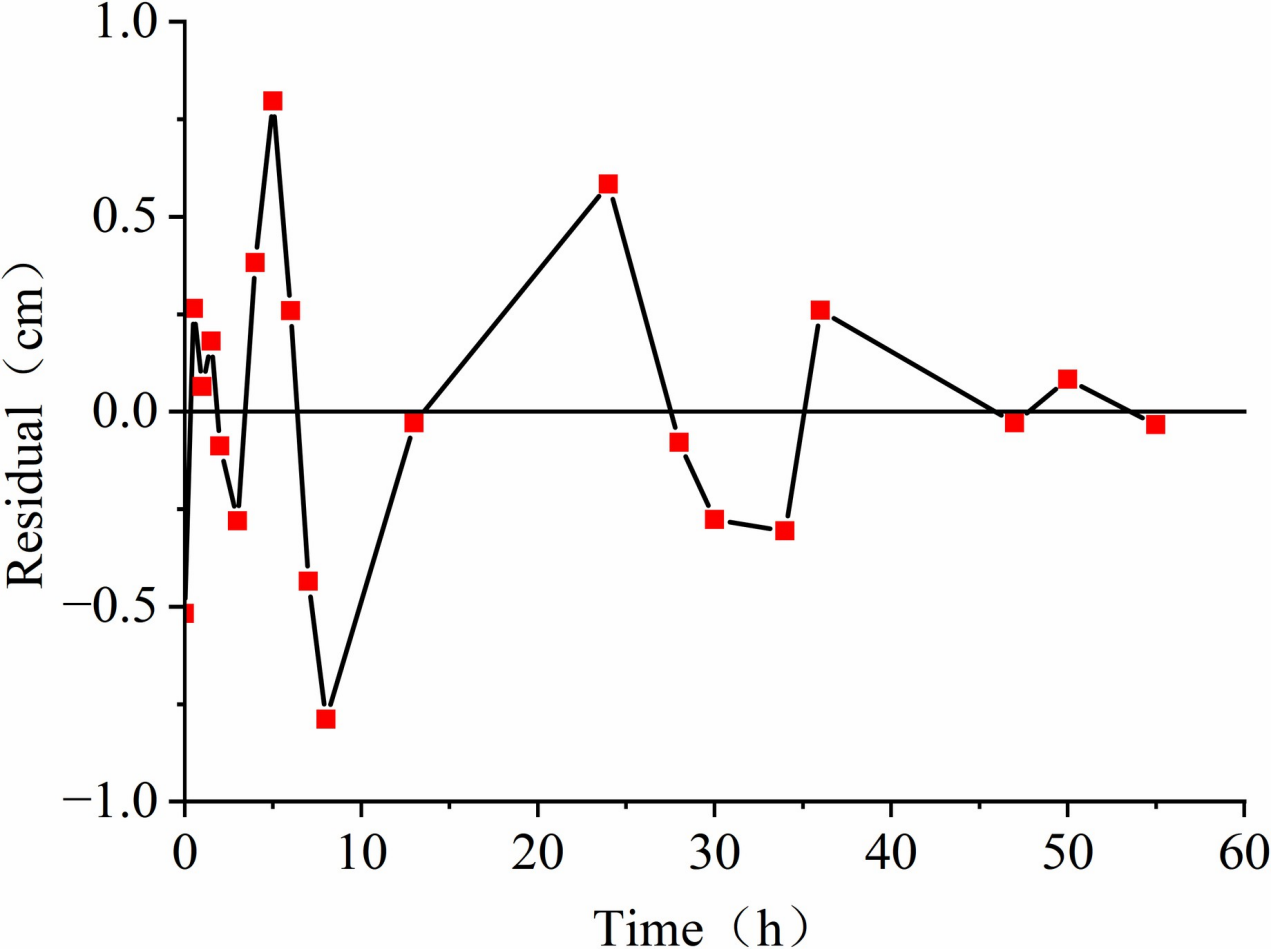
Residual distribution.

**Fig 6:**
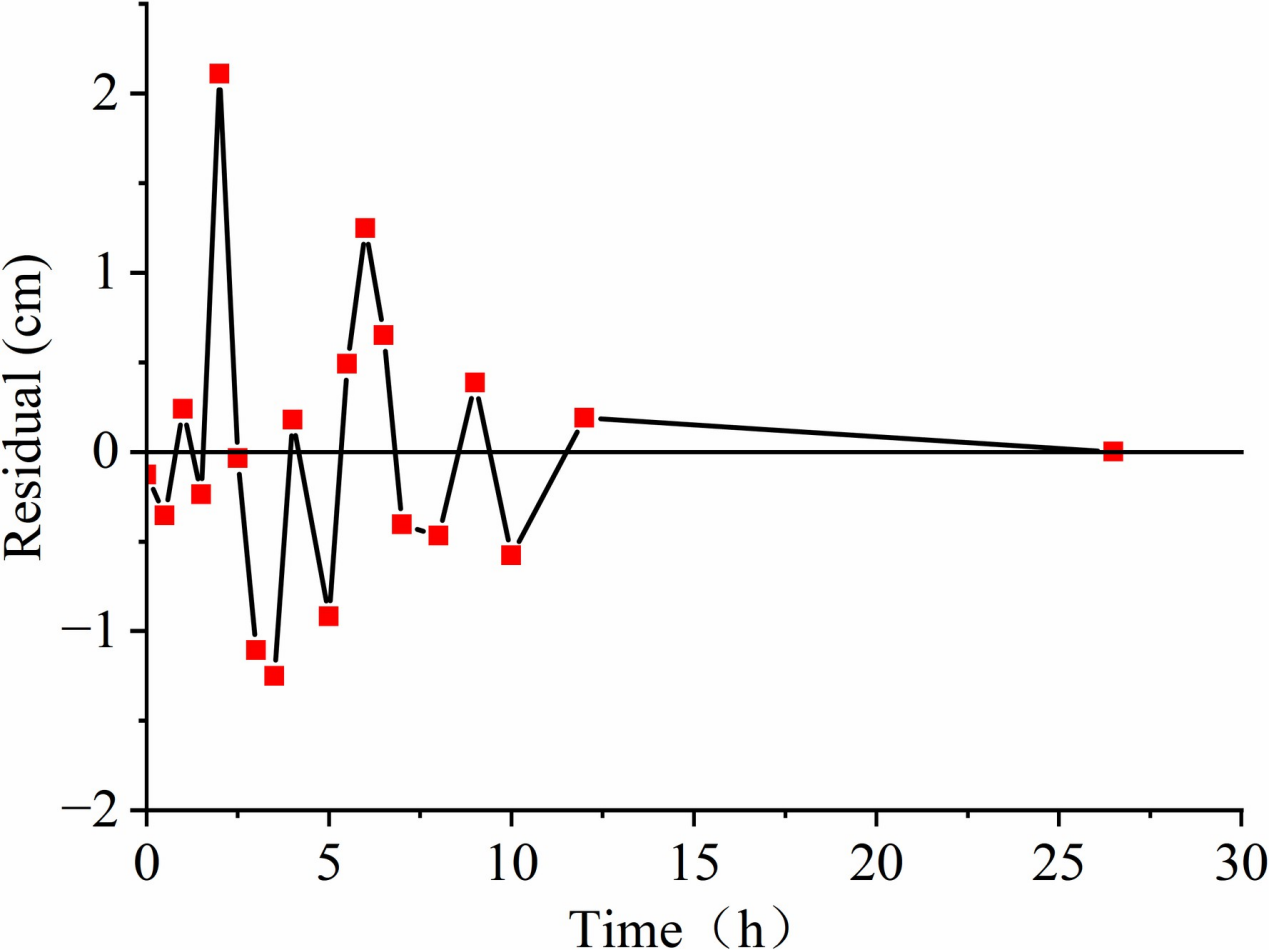
Residual distribution.

To examine the impact of groundwater conditions on LNAPL migration, we conducted three sets of experiments representing an unsaturated medium, a constant groundwater level, and a declining groundwater level. For the unsaturated state experiment, we obtained 24 sets of depth and time data, while for the constant water level and water level decline processes, we obtained 19 sets each. We used the Polyfit function in MATLAB to fit the data, which provided a polynomial expression for depth (h) in centimeters as a function of time (t) in hours: $h=p_{1}x^{n}+p_{2}x^{n-1}+\ldots \ldots p_{n}x+p_{n+1}$, By utilizing the diff function in MATLAB, the relationship between time t (h) and depth h (cm) was differentiated to determine the relationship between time t (h) and velocity v (cm/h): $v=np_{1}x^{n-1}+(n-1)p_{2}x^{n-2}+\ldots \ldots p_{n}$. Plotting **Figs 4–6** based on the functional relationships reveals that the measured values and the fitted values are distributed around 0 cm, mostly within the range of centimeters. This observation indicates that the fitting curve is reasonably accurate and reflects the real situation.

The Miller Soil Box experiment utilizes a direct current (DC) power supply. By connecting a voltmeter and ammeter in the circuit, researchers can measure the voltage and current within the circuit. The electrical conductivity formula (Eq 3) can be used to determine the conductivity corresponding to each LNAPL content:

$$G=\frac{I}{V}$$ (3)

In Formula (3), G represents electrical conductivity, I represents current intensity, and V represents voltage.

Based on the G derived from Eq (3) and the known distance between electrodes MN, the conductivity can be calculated using Eq (4).


$$\sigma =\frac{G}{L}$$ (4)

Where σ represents electrical conductivity, and L represents the distance between electrodes MN. Subsequently, a graph illustrating the relationship between LNAPL content and resistivity is plotted.

### 3.2 Analysis of the results

#### 3.2.1 Influence of LNAPL content on resistivity

The Miller Soil Box experiment investigated the relationship between changes in resistivity and varying LNAPL contents and water contents.

**Fig 7:**
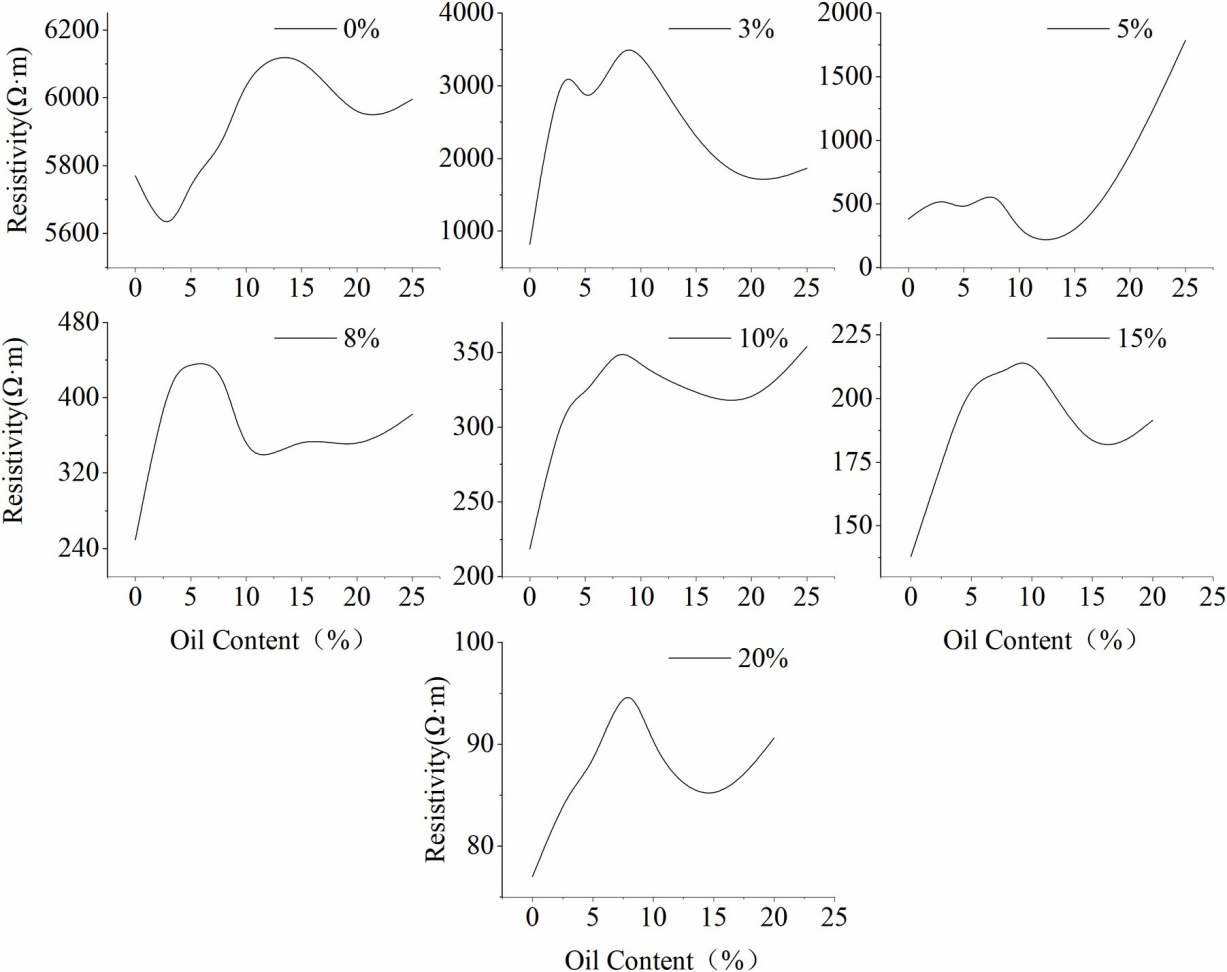
Variation of resistivity with LNAPL content.

**Fig 7** illustrates the trend of resistivity changes with varying LNAPL content under different water contents. The results indicate that when the water content is 0%, the resistivity of the sand sample with 3% LNAPL content is lower compared to the sample without added LNAPL. However, as the water content increases, the change in resistivity does not follow a similar trend. This is because the initial presence of pores in the medium results in relatively high resistivity, and the addition of LNAPL fills some of the pore spaces, reducing the overall resistance of the sand sample and causing the resistivity to decrease. Subsequently, due to the presence of water, the pores are filled with water, and this phenomenon no longer occurs.

Further analysis of the resistivity changes with increasing LNAPL content at different water contents reveals that under the coexistence of gas, oil, and water in a three-phase system, the resistivity of the medium, although subject to minor fluctuations, generally follows a similar overall trend. It exhibits an increasing-decreasing-increasing pattern with the increase of LNAPL content, and overall, it tends to increase.

**Fig 8:**
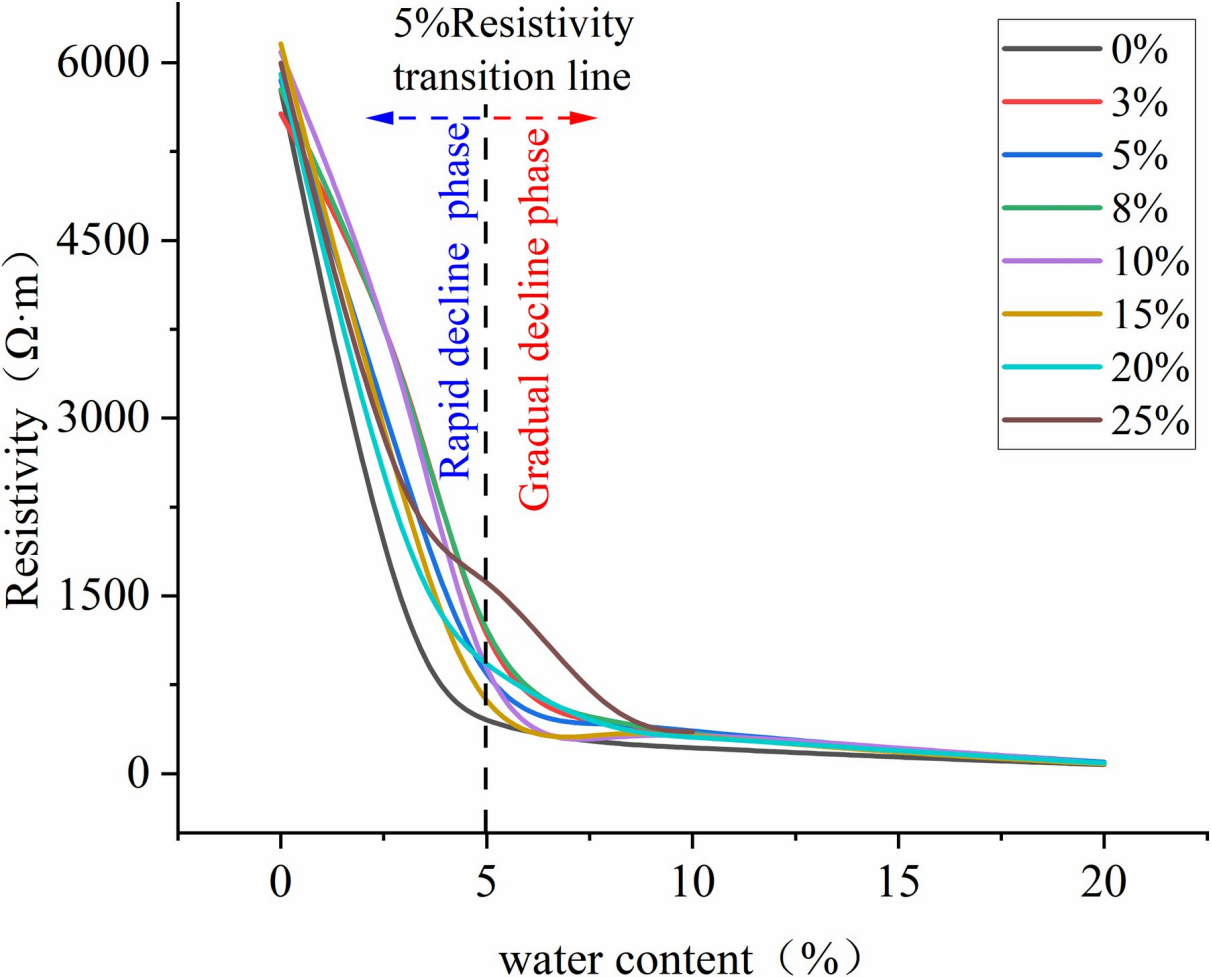
Trend of resistivity changes with increasing water content at different LNAPL contents.

**Fig 8** presents the trend of resistivity changes with increasing water content at different LNAPL contents. It can be observed that as the water content increases, the resistivity changes for different LNAPL contents follow a generally consistent trend. This trend can be divided into two stages, demarcated by a water content of 5%. When the water content is less than 5%, the medium’s resistivity rapidly decreases with increasing water content. When the water content exceeds 5%, the medium’s resistivity steadily decreases with further increases in water content. Since the resistivity for all LNAPL contents changes dramatically at a water content of 5%, this phenomenon may be related to the content of conductive ions in the pores [21]. As the water content increases from 0 to 5%, the originally high-resistivity medium gains a good conductor for electric current due to the addition of water, leading to a rapid decrease in resistivity. However, when the water content surpasses 5%, the moisture in the medium’s pores has reached a certain level, and the current density has essentially reached its maximum, thus the trend of resistivity decrease becomes stable.

From the above, it is evident that when LNAPL migrates underground, the magnitude of the medium’s resistivity is primarily influenced by the water content, while the trend of resistivity change with LNAPL content remains essentially unchanged. Moreover, the resistivity becomes more stable when the water content exceeds 5%. Additionally, soil resistivity is positively correlated with the saturation of LNAPL in the soil. An increase in LNAPL can significantly alter the soil’s resistivity, especially under conditions of low water content. This result resonates with Ansari, S.’s (2021) study on the impact of changes in soil physical properties on LNAPL migration [8]. As the water content increases, the effect of LNAPL on enhancing soil resistivity gradually diminishes. During the injection of LNAPL, the changes in resistivity reflect the vertical and horizontal transport and diffusion of LNAPL within the soil matrix.

#### 3.2.2 Characteristics of LNAPL migration in unsaturated media

**Fig 9:**
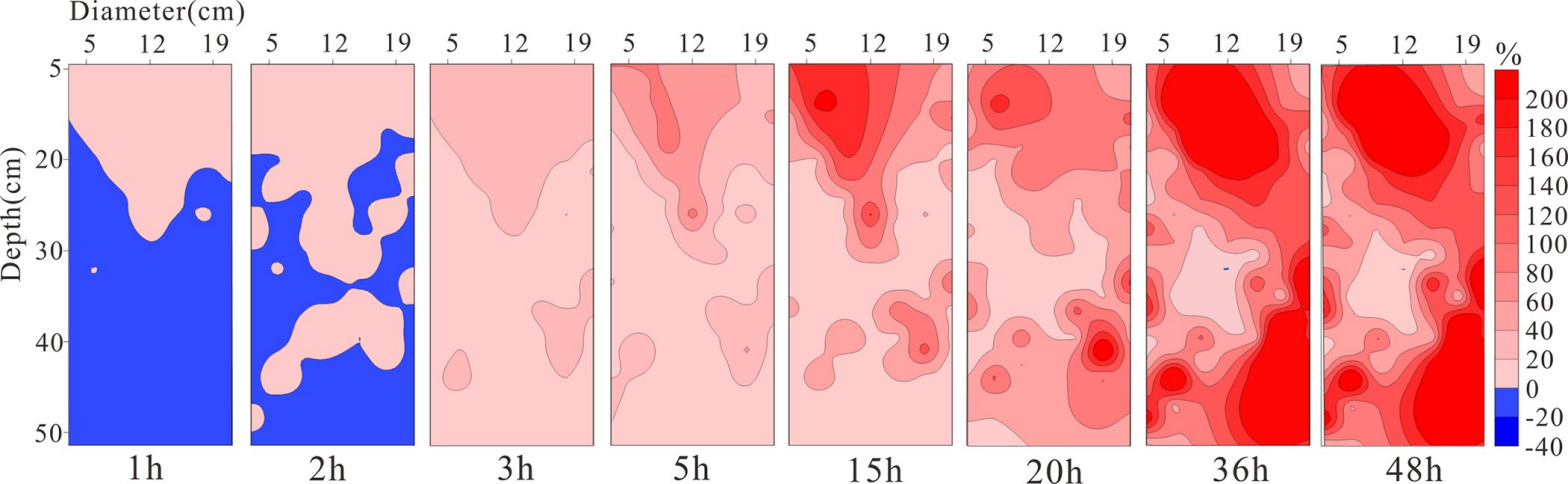
The rate of resistivity change under unsaturated conditions at different times.

**Fig 10:**
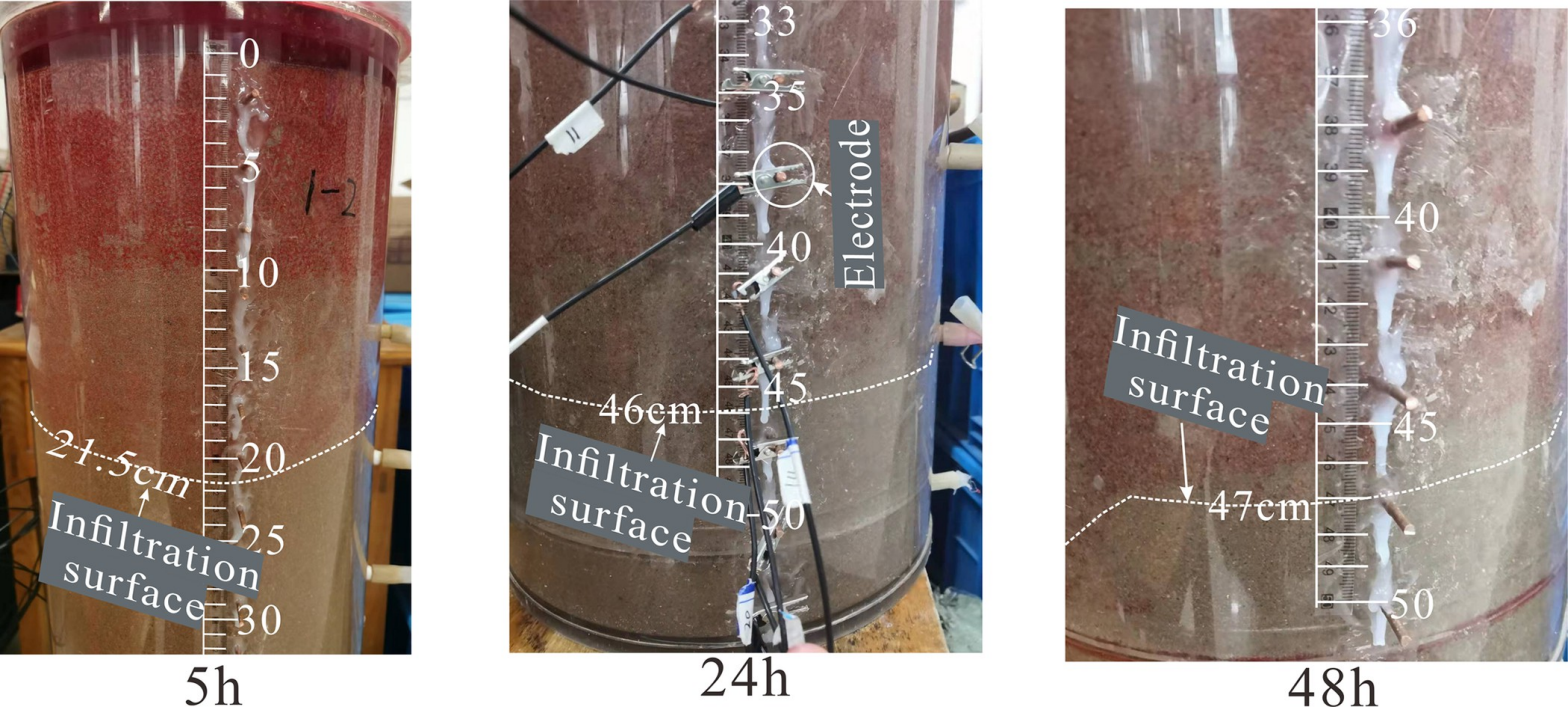
Photographs of LNAPL migration positions at different times.

**Fig 9** presents the distribution results of relative resistivity changes under unsaturated conditions at different times. With the injection of LNAPL, the resistivity of the lower layer of the medium shows a negative increase from 0h to 2h, indicating a decreasing trend in resistivity. This is due to the vertical migration of LNAPL within the medium under the influence of gravity, which continuously displaces water in the pores to accumulate below, thereby reducing the resistivity of the lower layer of the medium. After 3h, the overall resistivity of the medium shows an increasing trend, but the main changes occur in the upper part of the medium before 15h. From 20h to 48h, the rate of change in resistivity increases over time, and it is evident from the figure that after LNAPL migrates vertically from the right side to the bottom and is obstructed, it then migrates horizontally. This corresponds to the LNAPL migration front shown in **Fig 10**, where a real photograph taken at 5h shows the front at 21.5cm, at 24h the front is located at 46cm, and by 48h the front line shows one side at the 47 cm position and the other side has essentially reached the bottom.

**Fig 11:**
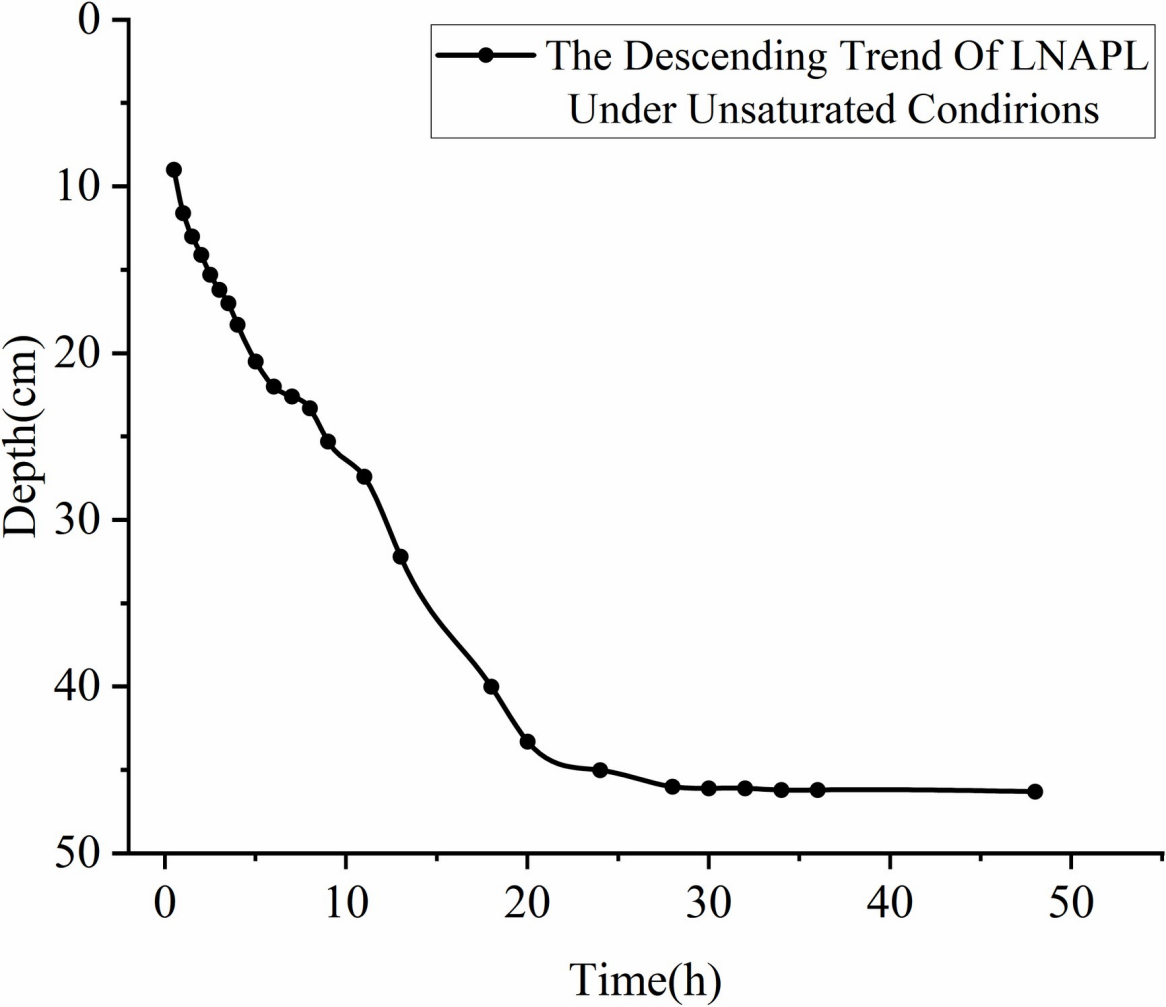
Depth of LNAPL migration in unsaturated conditions over time.

**Fig 12:**
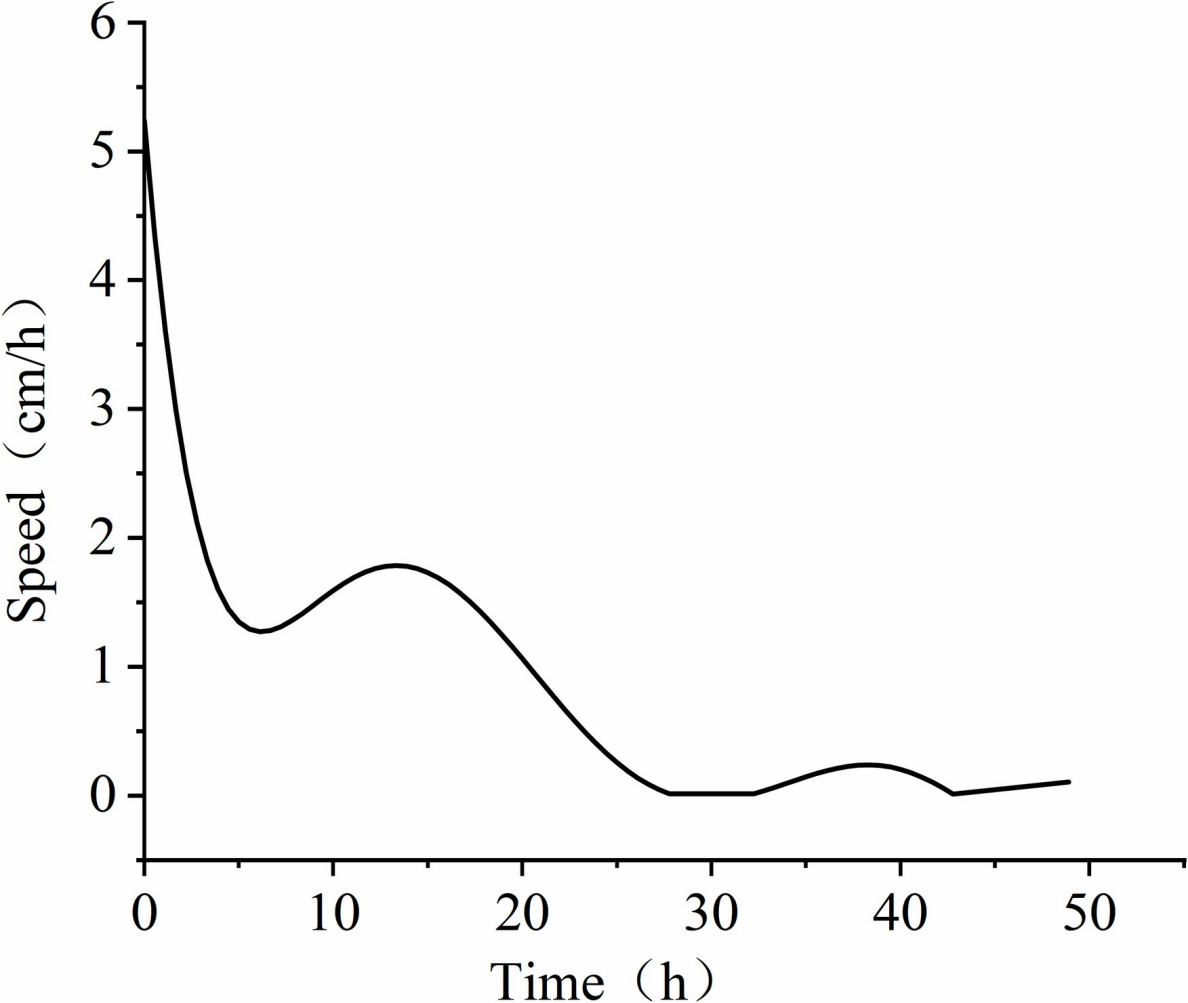
Variation of LNAPL migration rate over time in unsaturated conditions.

Further analysis of the time-depth plot in **Fig 11**, it is evident that LNAPL continuously migrates downward within the unsaturated medium under the influence of gravity. The migration depth steadily increases over time, but the capillary resistance slows down the migration rate. At 24 hours, the migration depth of LNAPL reaches approximately 46 cm, and it no longer increases with time, indicating a near cessation of vertical migration. The time-rate plot (see **Fig 12**) demonstrates the variation in LNAPL migration rates at different time points. The rate gradually decreases after LNAPL enters the medium. At 5 hours, the depth is 21.5 cm, and the rate shows a fluctuating pattern. From the addition of LNAPL to 5 hours, the rate rapidly decreases from 5.2 cm/h to 1.3 cm/h. It then increases to 18 cm/h between 5.5 hours and 14 hours. After 14.5 hours, a slow decline begins, and by 30 hours, the rate drops to 0.02 cm/h before starting to rise again. At 39 hours, the rate increases to 0.2 cm/h and then declines again, reaching the lowest point of 0 cm/h by 48 hours. The average migration rate from the beginning to the end is 1.06 cm/h.

**Fig 13:**
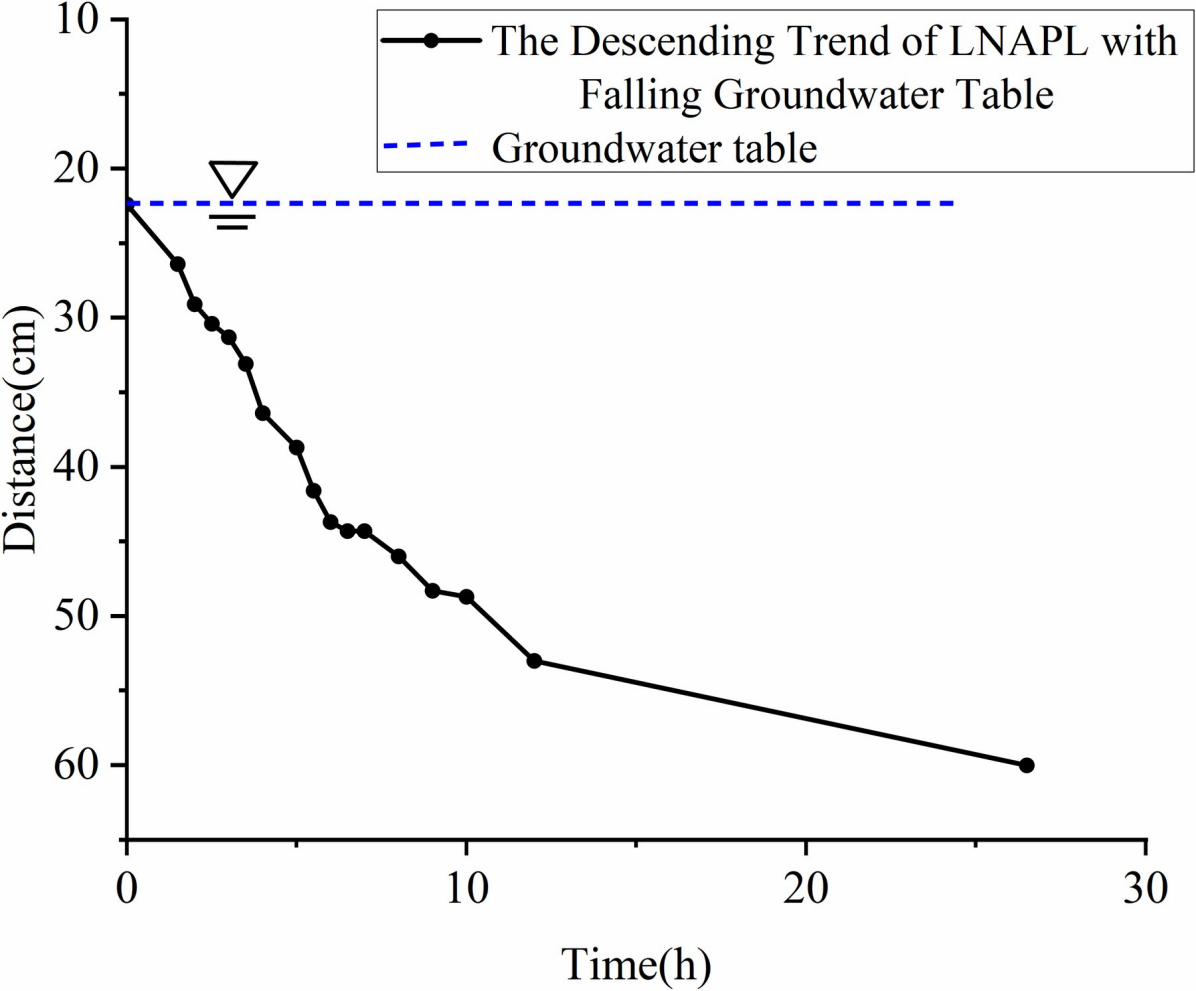
The depth of LNAPL migration as the groundwater table declines over time.

In unsaturated conditions, LNAPL migration is a displacement process that involves displacing air and residual water. As LNAPL moves downward, it covers the entire infiltration surface, causing water and a significant portion of gases to be expelled through drainage holes at the bottom. This displacement mechanism, similar to the infiltration method shown in **Fig 13** with decreasing water levels, is known as piston infiltration and leads to fluctuations in migration rates. At a depth of 21.5 cm, when water accumulates, the rate at which LNAPL displaces water exceeds the drainage rate of the drainage holes, hindering vertical migration. This results in lateral diffusion, which is consistent with the resistivity inversion results shown at 5 h in **Fig 9**. Once the water is displaced, the resistance to vertical migration weakens, leading to an increase in migration rate. However, as the depth increases, capillary resistance grows, causing a gradual decrease in migration rate. In **Fig 9**, at 20 h, a dominant migration pathway is observed on the right side. This is primarily due to the accumulation of most coarse particles on the right side during the artificial filling of the non-uniform medium, creating a pore structure with higher porosity on the right than on the left.

**Fig 14:**
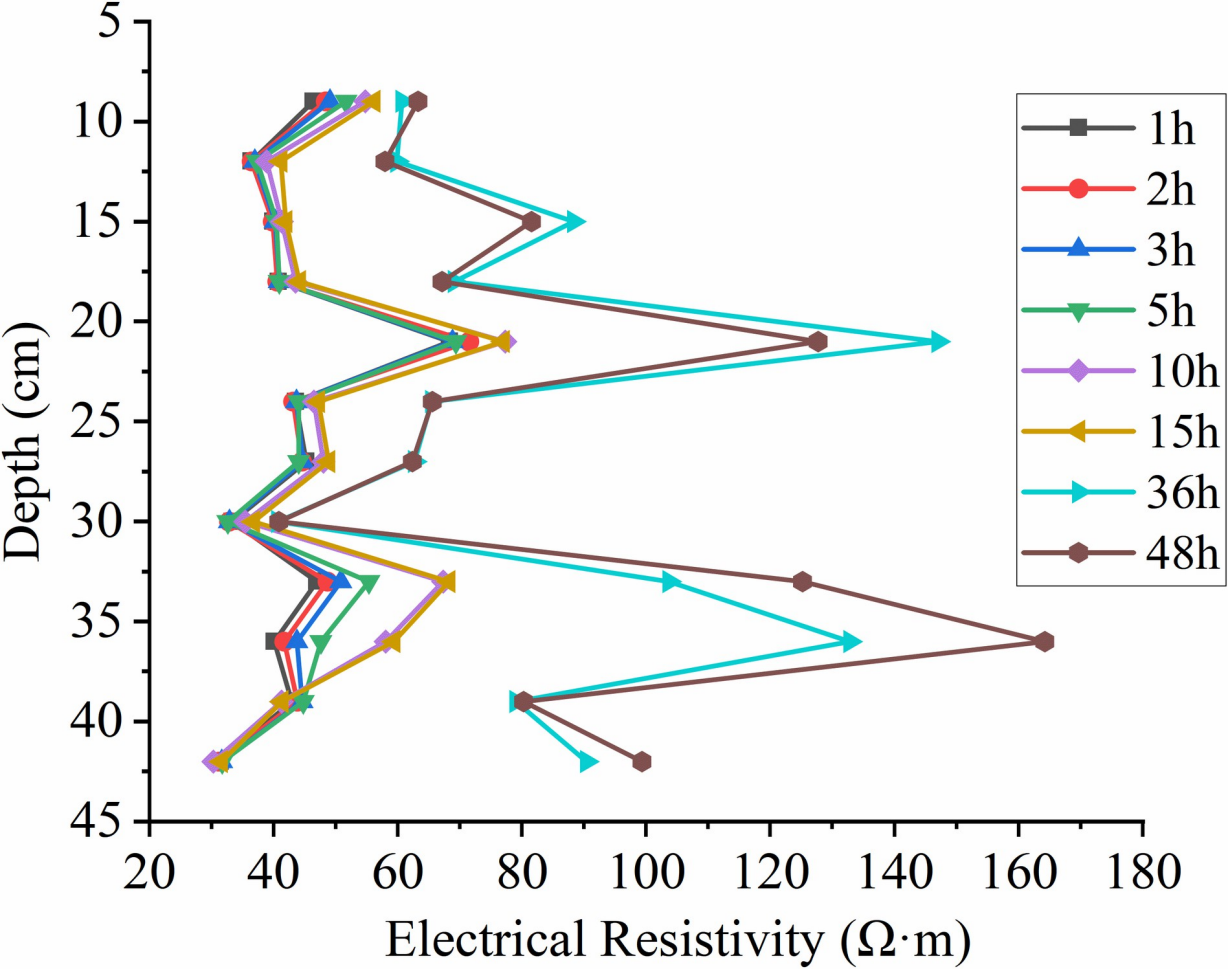
The resistivity at a depth of 18cm at different time points under unsaturated conditions varies with depth.

When extracting resistivity profiles at different times from the inverted profiles along the distance at a depth of 18 cm from the left side, as shown in **Fig 14**, it is observed that the resistivity variation with depth follows a consistent trend at different time points. There is a sudden increase in resistivity at a depth of 21.5 cm, followed by a decrease at a depth of 30 cm, where the resistivity values are relatively close. As the depth increases, the resistivity first increases and then decreases at various time points. Between 1 h and 15 h, the resistivity values remain essentially the same around a depth of 42.5 cm. However, at 36 h and 48 h, the resistivity values are larger and exhibit an increasing trend.

This location is close to the dominant migration pathway, as indicated by the time-depth plot in **Fig 11**. The migration depth reaching 21.5 cm encounters vertical migration obstruction due to capillary resistance and the effects of displacing air and water. LNAPL first reaches this depth at 5 h, and from 10 h to 48 h, the resistivity variation trend at this depth remains consistent, indicating significant capillary resistance at this location. At a depth of 30 cm, the resistivity values at different time points are similar, with a slight increase as time progresses. Based on the relative resistivity change distribution results in **Fig 9**, it can be observed that preferential pathways primarily exist at this depth, indicating that these preferential pathways are the main routes for LNAPL migration, and under these conditions, only a small amount of LNAPL underwent lateral migration.

#### 3.2.3 Migration characteristics of LNAPL under conditions of constant groundwater table and groundwater table drops

Unlike the unsaturated state, the presence of a groundwater table isolates the drainage holes, forming a sealed space with the LNAPL. Air and water in the medium are displaced, and since the pressure cannot be released, the force and resistance will eventually reach a balance. Under the action of the buoyant force, LNAPL ceases its vertical migration.

**Fig 15:**
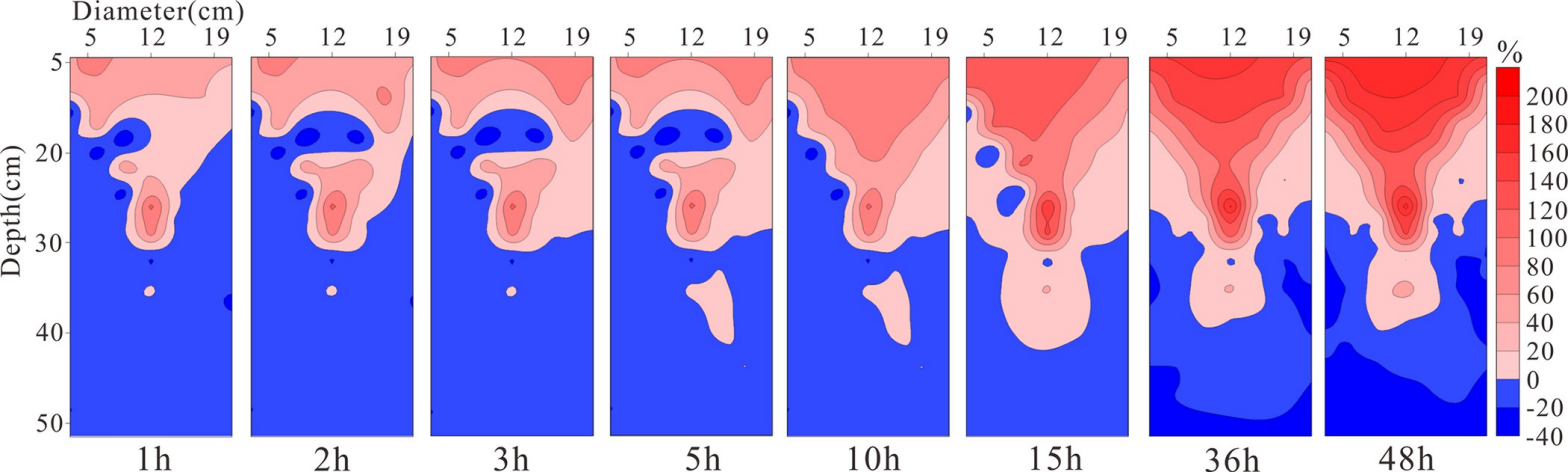
Rate of resistivity change at different times under constant groundwater table conditions.

**Fig 15** presents the distribution of relative resistivity changes at different times under conditions of a constant groundwater table. The water table is located at 23 cm. It can be observed that, due to the presence of the groundwater table, the resistivity changes in the medium during the 0h to 3h period are mainly concentrated in the upper layer near the water table. From 5h to 48h, the resistivity changes move downward and gradually expand horizontally. This is because, with the increase in LNAPL content, a small amount of LNAPL dissolves in the water phase at the oil-water interface, leading to a relative increase in resistivity. The stratification of resistivity changes is deeper than the actual position of the water table, but the overall stratification is distinct. It is a normal phenomenon for the resistivity range in ERT imaging to exceed the actual range, and this situation can be well improved by incorporating constrained inversion in data processing [22].

**Fig 16:**
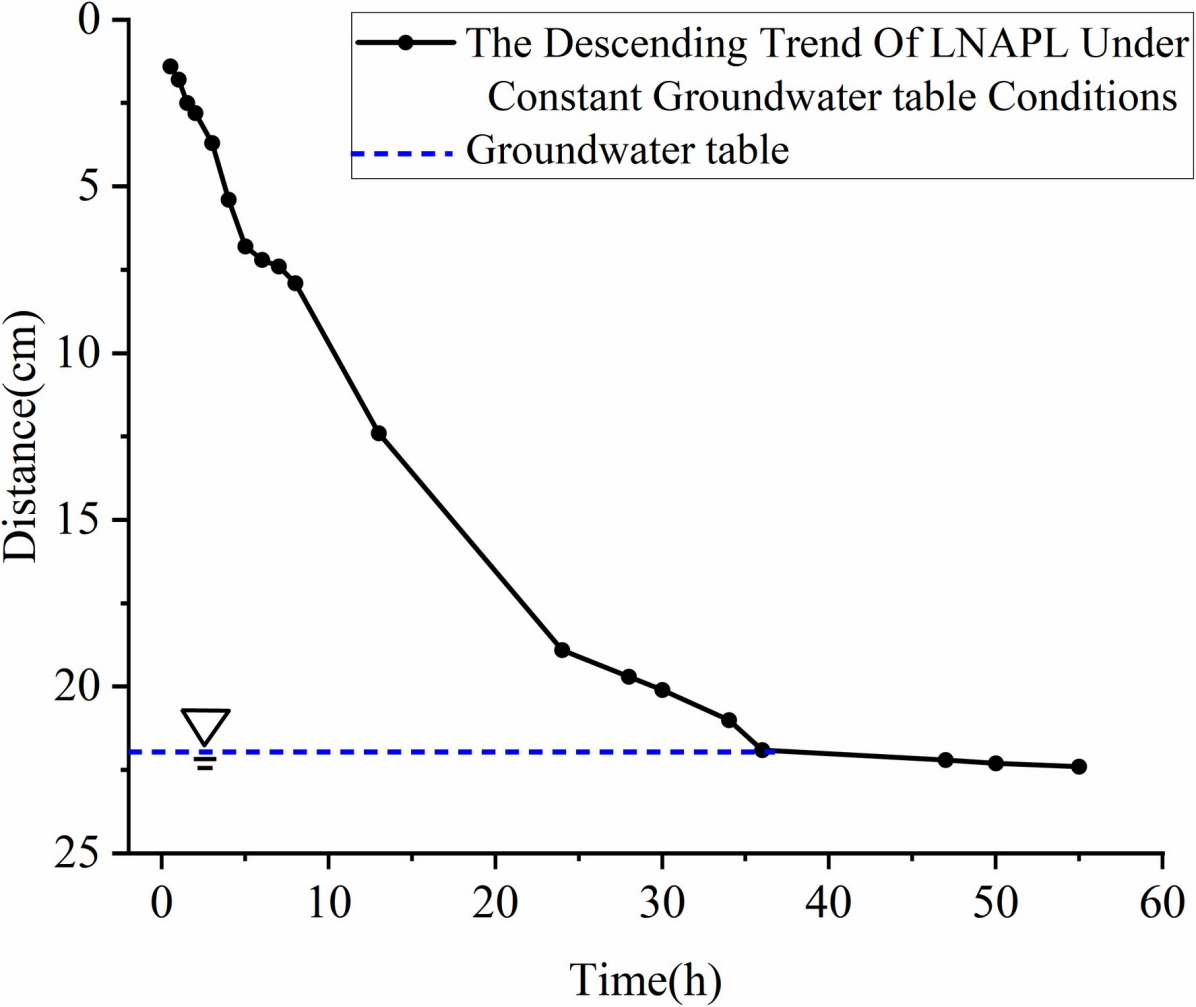
The Depth of LNAPL migration under constant groundwater table conditions over time.

**Fig 17:**
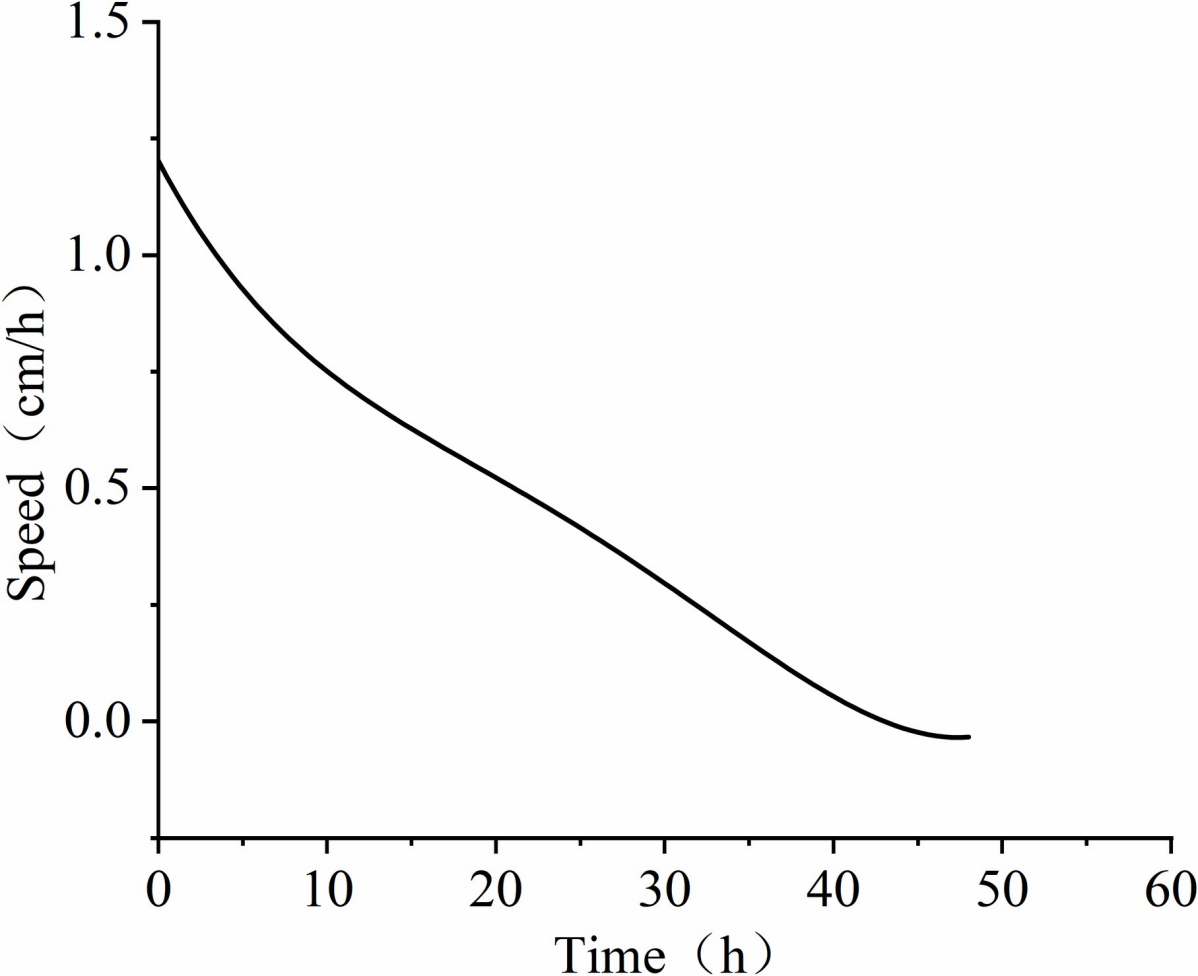
The Rate of LNAPL migration under constant groundwater table conditions over time.

From **Fig 16**, it can be observed that after reaching a depth of 22.4 cm, the migration depth essentially no longer increases with time, with the groundwater table located at 23 cm. As LNAPL migrates near the groundwater table, when the vertical gravitational force reaches equilibrium with capillary resistance, buoyancy, and gas pressure, the vertical migration transitions to horizontal migration. **Fig 15** shows that LNAPL infiltrates in a conical pattern, similar to the unsaturated experiments. **Fig 17** reveals that the rate of migration decreases linearly, approaching 0 cm/h at 48 hours, which is when LNAPL reaches the water table. The average migration rate is 0.51 cm/h.

**Fig 18:**
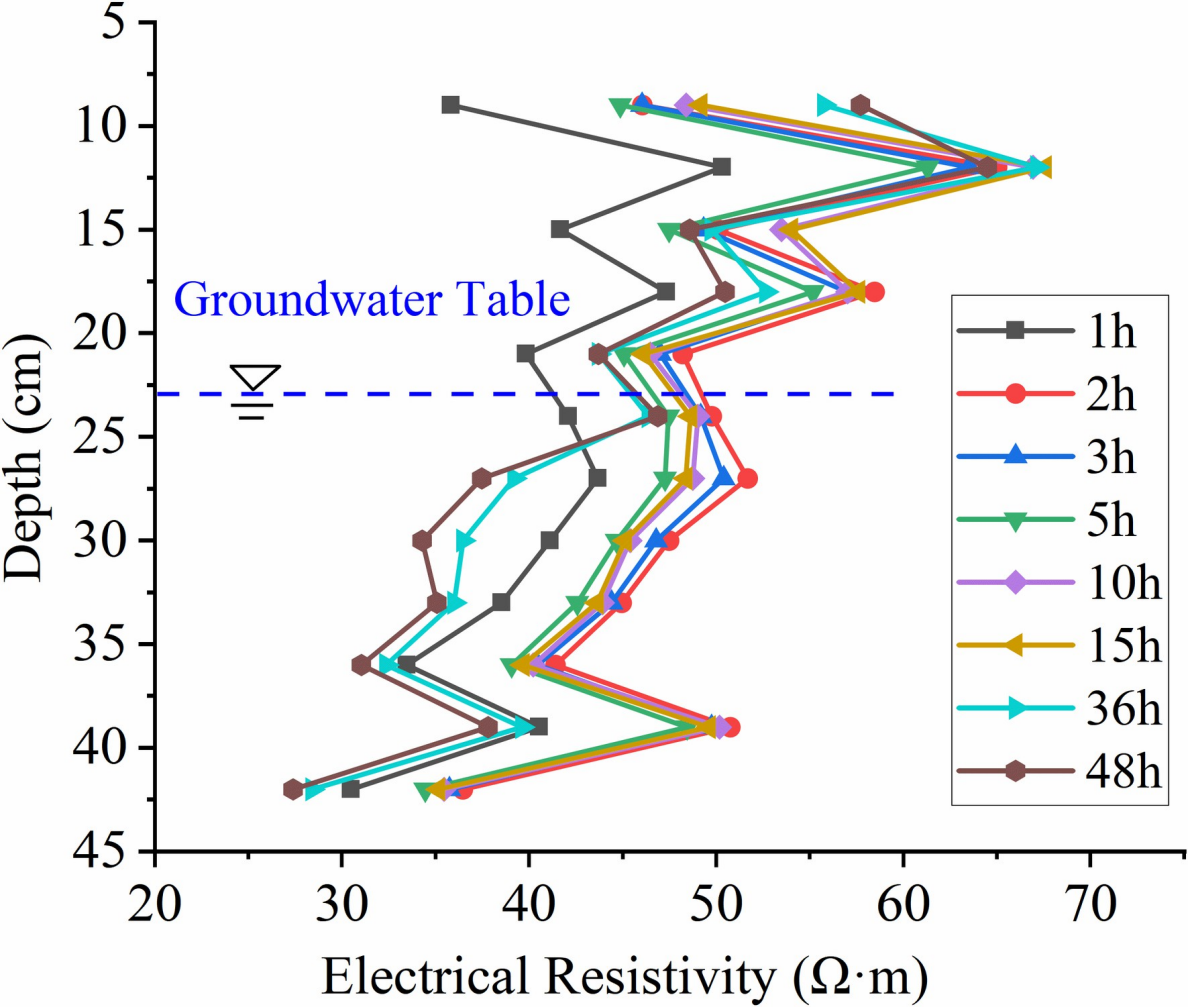
Resistivity variation with depth at different times under constant groundwater table conditions.

In this state, the resistivity extracted at 18 cm (as shown in **Fig 18**) generally follows a consistent trend with depth. There is a widespread decrease in resistivity at a depth of 22.4 cm, which, as can be seen from **Fig 16**, is near the position of the water table. The increasing trend in resistivity with depth is due to some LNAPL dissolving in water, causing an increase in resistivity at this location.

When the water table drops, the equilibrium is disrupted and the resistance suddenly decreases. As shown in **Fig 13**, LNAPL begins to migrate downward.

**Fig 19:**
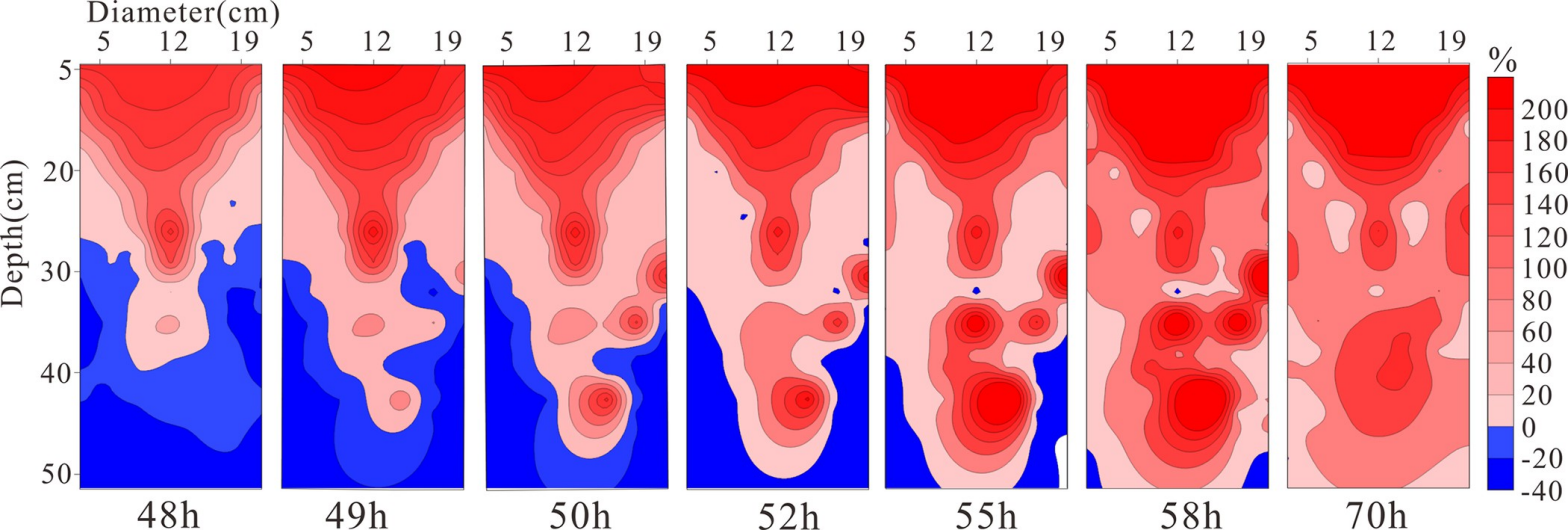
Resistivity change rate under declining groundwater table conditions at different times.

**Fig 19** displays the distribution of relative resistivity changes at different times under conditions of declining groundwater levels. From 48h to 52h, it can be observed that as the water level declines, the resistivity of the upper layer of the medium gradually increases with a rate of change greater than at deeper locations, and the area of change gradually diffuses towards the deeper parts, reaching the bottom by 52h. Between 52h and 70h, the positively changing resistivity in the medium spreads from the center to both sides at deeper levels. This indicates that LNAPL migrates more rapidly in the middle, and on both sides of the medium, the water is displaced by LNAPL towards the sides, leading to an increase in water content and a corresponding negative increase in resistivity. After reaching the bottom, the vertical migration is hindered and transitions into horizontal migration.

**Fig 20:**
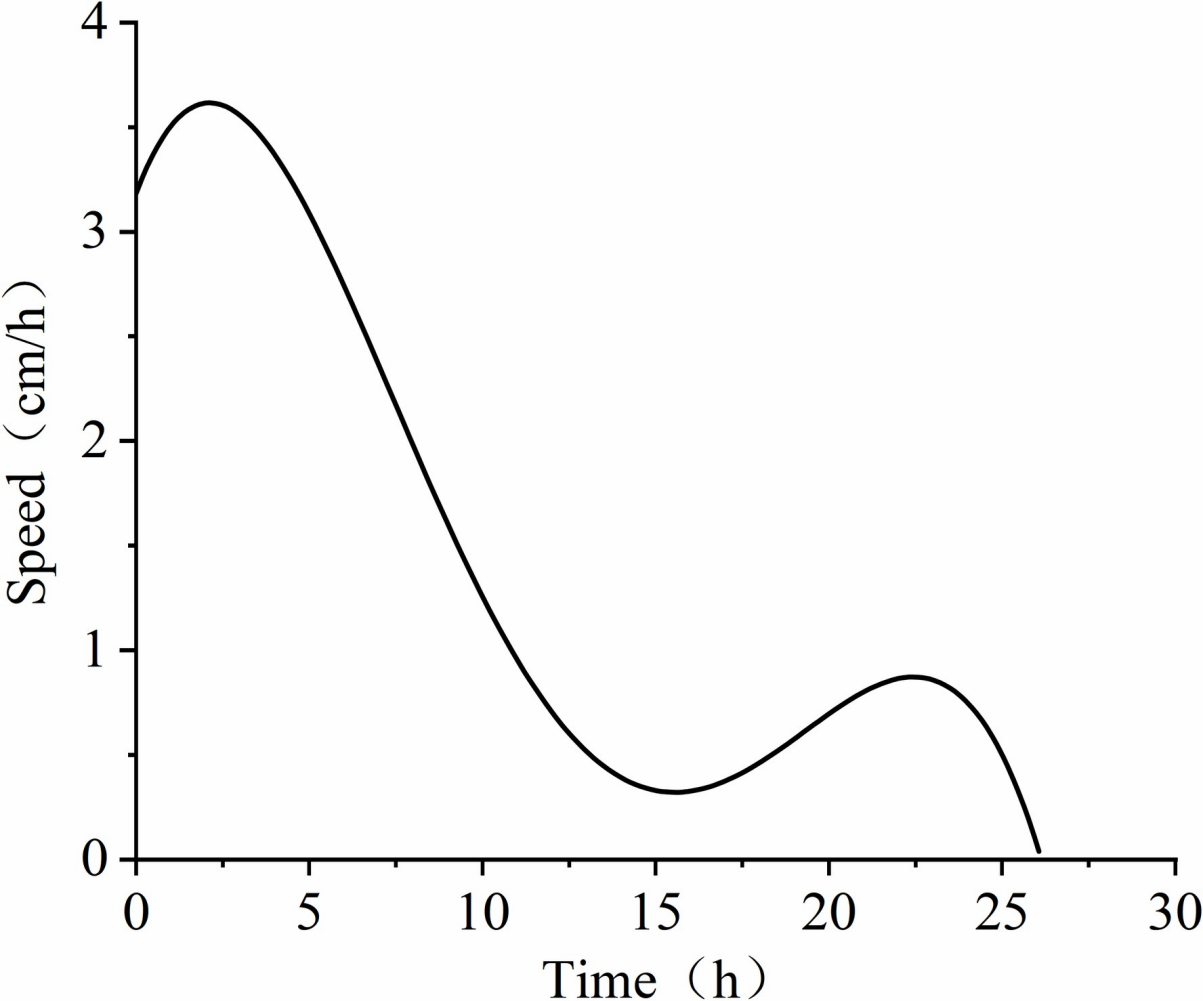
The rate of LNAPL migration as the groundwater table declines over time.

As can be seen from **Fig 20**, the migration rate suddenly increases after the initiation of migration. This is due to the release of pressure, and the process of declining groundwater also forms preferential flow paths, providing a conduit for the migration of LNAPL. Under the action of capillary pressure, the descent rate of the oil in the upper part is less than that of the water, creating a low-pressure tension in the middle that counteracts the water, leading to a decrease in the water flow speed. After the pressure is balanced, the water resumes its migration rate, and this cycle repeats, causing some air to be expelled upward from the oil medium, resulting in fluctuations in the migration rate. The average migration rate is 1.45 cm/h. This phenomenon is consistent with the findings of Meng Jian and Dong Yanhui (2022), who characterized LNAPL contamination plumes using ERT technology [13].

**Fig 21:**
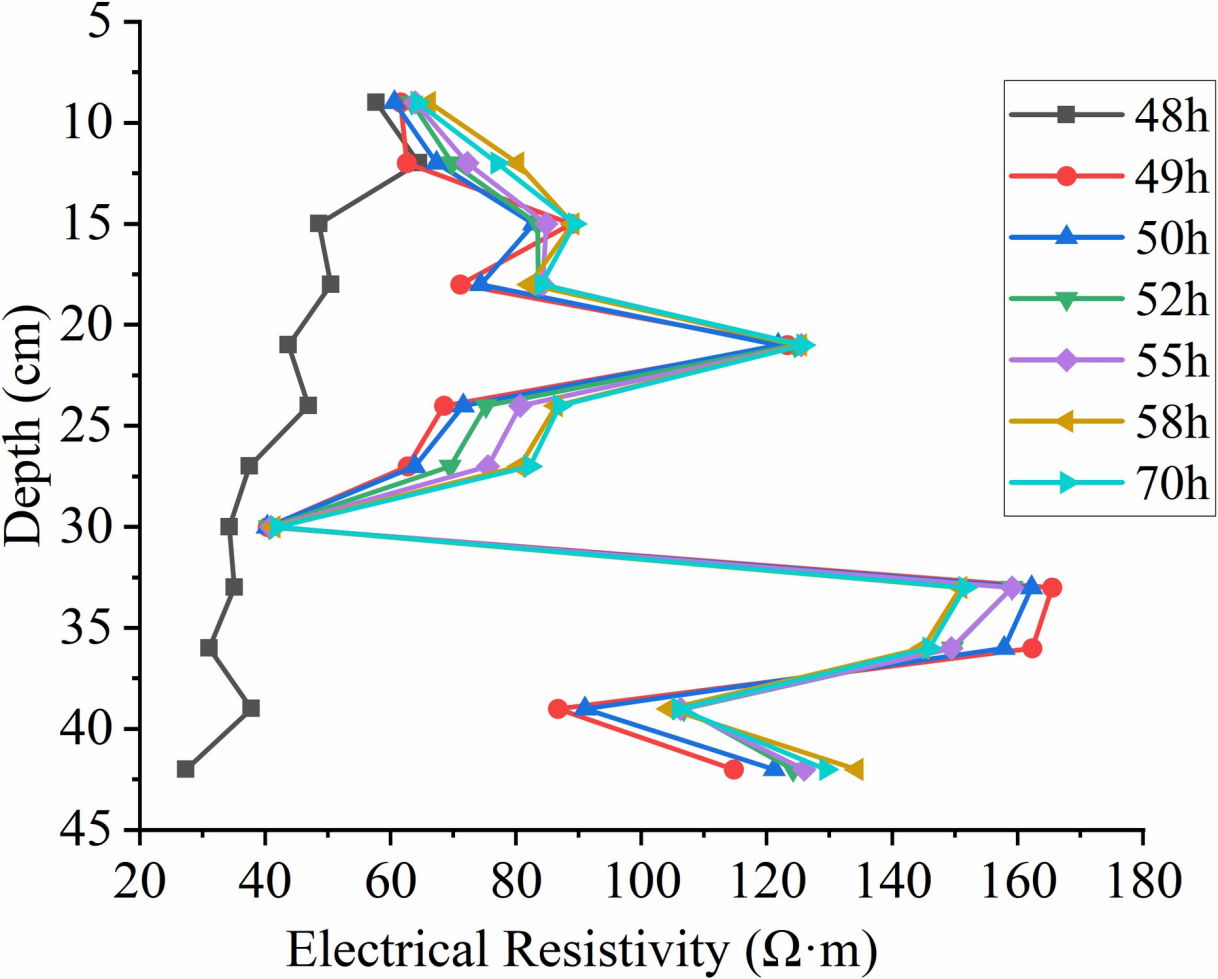
Resistivity variation with depth at different times during groundwater table decline.

According to **Fig 21**, which shows the changes in resistivity with depth at different times as the groundwater table declines, it can be observed that the resistivity significantly increases between 49h and 70h after the water table drops. The first peak in resistivity occurs near the original water table position at 23 cm depth. At a depth of 30 cm, the resistivity suddenly decreases to essentially the same value and then increases with depth, showing a second peak near 35 cm depth before starting to decrease again. Overall, it follows a pattern of increase—decrease—increase.

Due to the decline in the groundwater table, LNAPL begins to migrate downward, and the rapid increase in LNAPL content in the medium leads to an increase in resistivity. The 23 cm depth is the position of the original water table, and the resistivity changes significantly as LNAPL migrates when the water table drops. A decrease in resistivity to essentially the same value occurs near the 30 cm depth, and from **Fig 19**, it can be seen that the declining groundwater forms preferential flow paths, with LNAPL primarily migrating within these paths. Without external forces, it will not migrate in other directions, hence the resistivity at this depth remains essentially unchanged. As depth increases, the resistivity increases as well, indicating that the preferential flow paths are only present near the 30 cm depth.

From the above, it can be concluded that the migration of LNAPL during the decline of the groundwater table is primarily characterized by diffusion from the center outwards. The main reason for this pattern is the formation of preferential flow paths during the groundwater decline, which reduces the resistance to LNAPL migration.

## 4 Discussion

Our experimental findings reveal distinctive LNAPL migration behaviors under varying groundwater conditions, with migration rates of 1.06 cm/h (unsaturated), 0.51 cm/h (constant water table), and 1.45 cm/h (declining water table). These variations highlight the critical role of both groundwater conditions and medium heterogeneity in controlling LNAPL transport mechanisms.

The formation of preferential migration pathways in coarse-grained regions, particularly evident under unsaturated conditions and declining water table states, significantly influenced LNAPL transport. These pathways, characterized by reduced migration resistance, created channels for preferential LNAPL movement, demonstrating how medium heterogeneity can substantially impact contaminant diffusion patterns. This finding extends beyond laboratory observations to natural soil environments, suggesting that soil structural non-uniformity may accelerate localized pollution migration rates—a crucial consideration for remediation strategy development.

The observed "piston infiltration" mechanism in unsaturated conditions provides new insights into the complex interplay between gravity, capillary forces, and pore space dynamics. The rapid initial migration rate (5.2 cm/h) followed by significant deceleration indicates that the initial LNAPL infiltration is predominantly controlled by gravitational forces, while the later stages are increasingly dominated by capillary forces and pore space availability. This pattern indicates that initial LNAPL infiltration is dominated by gravitational forces, while later stages are increasingly controlled by capillary forces and pore space availability.

Our resistivity measurements revealed a critical threshold at 5% water content, marking a fundamental shift in the medium’s electrical properties. The increasing-decreasing-increasing pattern of resistivity with LNAPL content indicates complex phase interactions that cannot be explained by simple displacement models. The ERT technology employed in this study demonstrated significant advantages in providing high temporal resolution and non-destructive monitoring of resistivity changes under different groundwater conditions. However, several limitations must be acknowledged. The accuracy of ERT imaging is constrained by resistivity changes, and the complex interactions between water and LNAPL content can affect precision. Furthermore, in highly heterogeneous media, ERT may overestimate pollution migration extent. These limitations suggest the need for incorporating constrained inversion in data processing or combining ERT with complementary monitoring techniques for multi-dimensional validation.

As part of the verification experiments for the "Monitoring the Distribution and Migration of LNAPLs Based on 3D Borehole-to-Surface Resistivity Method" project, this paper has achieved some findings but also has some shortcomings: First, there is a lack of extensive testing on the migration characteristics under different soil types, different LNAPL substances, and various environmental conditions. Second, there is a lack of monitoring in actual field sites. Therefore, our future research will comprehensively analyze the impact of different soils and pollutants on LNAPL migration and implement the application of laboratory results to actual contaminated sites, in order to further explore and refine related studies. Contributing to environmental remediation.

Our findings highlight the need for a paradigm shift in approaching LNAPL contamination problems, moving from static, single-parameter monitoring to dynamic, multi-parameter assessment frameworks. This understanding is crucial for developing more effective strategies for preventing and remediating LNAPL contamination in diverse hydrogeological settings. The complex interactions between soil properties, groundwater conditions, and contaminant behavior underscore the importance of integrated approaches to environmental remediation.

## 5 Conclusion

This study conducted an in-depth analysis of the migration characteristics of LNAPL under various groundwater storage conditions using ERT cross-hole measurement methods, combined with high-definition camera monitoring. The experimental results indicate that the migration behavior of LNAPL is significantly affected by the conditions of the groundwater table, and changes in resistivity can effectively reflect the migration and distribution of LNAPL within the soil matrix.

Firstly, the Miller Soil Box experiment revealed the relationship between LNAPL content and resistivity. The study found that as the LNAPL content increased, the resistivity exhibited an increasing-decreasing-increasing pattern, and when the water content was greater than 5%, the changes in resistivity became stable. This indicates that under low water content conditions, the addition of LNAPL significantly increased the soil’s resistivity, and as the water content increased, the influence of LNAPL on resistivity gradually diminished.

In unsaturated media, the migration rate of LNAPL gradually decreases over time, and the relationship between migration depth and time shows a distinct nonlinear characteristic. Experimental data indicates that the average migration rate of LNAPL in unsaturated media is 1.06 cm/h, and during the migration process, fluctuations in the migration rate occur due to capillary resistance and displacement effects. Additionally, preferential migration pathways in unsaturated media significantly influence the migration path of LNAPL.

Under conditions with a constant groundwater table, the migration of LNAPL is restricted by the water table, and the migration depth essentially stops increasing after reaching near the water table line. At this point, the average migration rate of LNAPL is 0.51 cm/h, and changes in resistivity are mainly concentrated in the upper layer near the water table. When the groundwater table declines, the migration rate of LNAPL suddenly increases, with an average migration rate reaching 1.45 cm/h. The experiment found that under conditions of heterogeneous sandy media, LNAPL tends to form preferential migration pathways along areas where coarse particles aggregate. This not only accelerates the migration process of LNAPL but also further affects its vertical and horizontal diffusion patterns. Especially when the water table declines, the preferential pathways greatly reduce the migration resistance and enhance the diffusive ability of LNAPL within the soil matrix.

## Supporting information

S1 File (PDF)
