# Supplementary material for: A study on the monitoring of LNAPL migration using ERT
Source: PLoS One. 2025 Jan 24;20(1):e0315624. doi: 10.1371/journal.pone.0315624 (PMC11759388; doi:10.1371/journal.pone.0315624)
Supplement: S1 File — (PDF) [file pone.0315624.s001.pdf]

FIG4-1	Oil Content (Vertical Axis)Water Content	0%	3%	5%	8%	10%	15%	20%
	0%	5770.53	819.363	383.78	249.63	218.8	138.1	77.028
	3%	5568.25	3654.3	575.54	428.06	317.9	181.5	85.724
	5%	5772.45	2511.52	418.3	435.77	319.6	208.6	87.61
	8%	5849.35	3549.71	664.76	440.26	356.7	210.1	97.903
	10%	6090.68	3536.94	202.64	315.85	339.9	220	88.556
	15%	6159.66	2185.63	193.69	364.44	322.3	172.9	83.144
	20%	5903.27	1586.11	842.79	341.66	312.1	191.6	90.639
	25%	5996.64	1869.76	1786.1	382.78	353.9		

FIG4-2	Oil Content (Vertical Axis)Water	0%	3%	5%	8%	10%	15%	20%
	0%	5770.53	819.363	383.78	249.63	218.8	138.1	77.028
	3%	5568.25	3654.3	575.54	428.06	317.9	181.5	85.724
	5%	5772.45	2511.52	418.3	435.77	319.6	208.6	87.61
	8%	5849.35	3549.71	664.76	440.26	356.7	210.1	97.903
	10%	6090.68	3536.94	202.64	315.85	339.9	220	88.556
	15%	6159.66	2185.63	193.69	364.44	322.3	172.9	83.144
	20%	5903.27	1586.11	842.79	341.66	312.1	191.6	90.639
	25%	5996.64	1869.76	1786.1	382.78	353.9		

FIG5-1	1h(left)									
	X/m	resistivity	K	U	I	K*U/I		Y/m	X/m	(G-H)/H*100
	0.03	367.11	0.1884	6383.7	65.56	18.34	17.93	0.045	0.03	2.2999
	0.03	357.97	0.1884	6384.3	67.24	17.89	17.56	0.075	0.03	1.8397
	0.03	254	0.1884	6385	94.78	12.69	12.65	0.105	0.03	0.3277
	0.03	443.84	0.1884	6383.9	54.23	22.18	22.06	0.135	0.03	0.5332
	0.03	417.83	0.1884	6384.1	57.61	20.88	21.09	0.165	0.03	-1.008
	0.03	363.5	0.1884	6383.8	66.21	18.16	18.26	0.195	0.03	-0.523
	0.03	406.54	0.1884	6384	59.2	20.32	19.85	0.225	0.03	2.3744
	0.03	315.85	0.1884	6384.4	76.21	15.78	15.85	0.255	0.03	-0.452
	0.03	260.34	0.1884	6384.7	92.46	13.01	13.11	0.285	0.03	-0.74
	0.03	320.08	0.1884	6384.6	75.2	16	16.04	0.315	0.03	-0.296
	0.03	284.06	0.1884	6384.8	84.74	14.2	14.4	0.345	0.03	-1.397
	0.03	269.19	0.1884	6385	89.42	13.45	13.62	0.375	0.03	-1.246
	0.03	117.12	0.1884	6384.9	102.77	11.7	11.84	0.405	0.03	-1.138
	0.03	128.96	0.1884	6385	93.33	12.89	12.68	0.435	0.03	1.644
	0.03	123.12	0.1884	6385.1	97.77	12.3	12.5	0.465	0.03	-1.588
	0.06	474.85	0.3768	6384.4	76.04	31.64	31.24	0.09	0.06	1.2742
	0.06	459.34	0.3768	6384.6	78.61	30.6	30.28	0.12	0.06	1.0532
	0.06	413.4	0.3768	6384.6	87.34	27.54	27.61	0.15	0.06	-0.253

0.06	642.55	0.3768	6384	56.19	42.81	42.06	0.18	0.06	1.7762
0.06	651.03	0.3768	6384.2	55.46	43.37	43.84	0.21	0.06	-1.065
0.06	549.48	0.3768	6384.1	65.71	36.61	36.91	0.24	0.06	-0.813
0.06	626.06	0.3768	6384.2	57.67	41.71	41.56	0.27	0.06	0.3768
0.06	555.32	0.3768	6384.5	65.02	37	37.34	0.3	0.06	-0.913
0.06	436.09	0.3768	6384.9	82.8	29.06	29.32	0.33	0.06	-0.898
0.06	128.4	0.3768	6384.9	93.74	25.67	25.67	0.36	0.06	-0.036
0.06	154.29	0.3768	6384.4	78	30.84	30.06	0.39	0.06	2.5971
0.06	110.05	0.3768	6384.9	109.37	22	22.29	0.42	0.06	-1.329
0.09	613.06	0.5652	6384.7	78.53	45.95	44.33	0.135	0.09	3.6508
0.09	640.84	0.5652	6384.4	75.12	48.04	47.54	0.165	0.09	1.0493
0.09	554.18	0.5652	6384.9	86.87	41.54	41.92	0.195	0.09	-0.911
0.09	889.07	0.5652	6384.3	54.15	66.64	66.33	0.225	0.09	0.4611
0.09	987.42	0.5652	6384.3	48.75	74.02	74.37	0.255	0.09	-0.473
0.09	-1	0.5652	-1.55	0.18	-4.87	60.48	0.285	0.09	-108
0.09	167.35	0.5652	6384.4	71.92	50.17	50.21	0.315	0.09	-0.064
0.09	208.32	0.5652	6383.9	57.77	62.46	61.96	0.345	0.09	0.8049
0.09	149.78	0.5652	6384.5	80.36	44.9	45.06	0.375	0.09	-0.355
0.12	1		#N/A	#N/A	#N/A	#N/A	0.18	0.12	#N/A
0.12	160.53	0.7536	6383.3	74.96	64.17	62.87	0.21	0.12	2.079
0.12	208.27	0.7536	6383.2	57.78	83.25	82.8	0.24	0.12	0.5505
0.12	159.52	0.7536	6384.2	75.44	63.77	63.38	0.27	0.12	0.6199
0.12	259.03	0.7536	6384	46.46	103.6	102.2	0.3	0.12	1.3307
0.12	234.76	0.7536	6383.9	51.26	93.85	94.18	0.33	0.12	-0.352

2h(left)

X/m	resistivity	K	U	I	K*U/I		Y/m	X/m	(G-H)/H*100
0.03	482.55	0.1884	6384.7	49.89	24.11	17.93	0.045	0.03	34.453
0.03	439.72	0.1884	6385.3	54.75	21.97	17.56	0.075	0.03	25.092
0.03	302.56	0.1884	6385.7	79.57	15.12	12.65	0.105	0.03	19.519
0.03	495.8	0.1884	6385.2	48.56	24.77	22.06	0.135	0.03	12.294
0.03	447.51	0.1884	6385.3	53.8	22.36	21.09	0.165	0.03	6.022
0.03	416.47	0.1884	6385.5	57.81	20.81	18.26	0.195	0.03	13.962
0.03	475.79	0.1884	6385.4	50.6	23.77	19.85	0.225	0.03	19.801
0.03	363.86	0.1884	6385.9	66.17	18.18	15.85	0.255	0.03	14.68
0.03	308.91	0.1884	6386	77.94	15.44	13.11	0.285	0.03	17.776
0.03	381.6	0.1884	6385.9	63.09	19.07	16.04	0.315	0.03	18.867
0.03	332.61	0.1884	6386	72.39	16.62	14.4	0.345	0.03	15.447
0.03	316.02	0.1884	6386.1	76.19	15.79	13.62	0.375	0.03	15.922
0.03	139.1	0.1884	6386.2	86.55	13.9	11.84	0.405	0.03	17.414
0.03	150.32	0.1884	6386.2	80.09	15.02	12.68	0.435	0.03	18.469
0.03	143.95	0.1884	6386.1	83.63	14.39	12.5	0.465	0.03	15.07
0.06	634.74	0.3768	6385.2	56.89	42.29	31.24	0.09	0.06	35.381
0.06	587.03	0.3768	6385.5	61.52	39.11	30.28	0.12	0.06	29.144
0.06	488.53	0.3768	6385.5	73.92	32.55	27.61	0.15	0.06	17.873
0.06	716.29	0.3768	6385.3	50.41	47.73	42.06	0.18	0.06	13.468
0.06	696.67	0.3768	6385.4	51.84	46.41	43.84	0.21	0.06	5.863
0.06	625.51	0.3768	6385.8	57.74	41.67	36.91	0.24	0.06	12.907
0.06	723.45	0.3768	6385.6	49.92	48.2	41.56	0.27	0.06	15.984
0.06	637.83	0.3768	6385.8	56.62	42.5	37.34	0.3	0.06	13.81

0.06	512.61	0.3768	6386.1	70.45	34.16	29.32	0.33	0.06	16.497
0.06	154.3	0.3768	6386.1	78.02	30.84	25.67	0.36	0.06	20.128
0.06	186.85	0.3768	6385.8	64.43	37.35	30.06	0.39	0.06	24.233
0.06	129.17	0.3768	6386.3	93.2	25.82	22.29	0.42	0.06	15.815
0.09	821.33	0.5652	6385.4	58.62	61.57	44.33	0.135	0.09	38.87
0.09	818.56	0.5652	6385.3	58.82	61.36	47.54	0.165	0.09	29.071
0.09	650.01	0.5652	6385.9	74.08	48.72	41.92	0.195	0.09	16.215
0.09	978.67	0.5652	6385.5	49.2	73.36	66.33	0.225	0.09	10.589
0.09	1055.04	0.5652	6385.6	45.64	79.08	74.37	0.255	0.09	6.3304
0.09	908.66	0.5652	6385.9	52.99	68.11	60.48	0.285	0.09	12.625
0.09	193.47	0.5652	6385.9	62.22	58.01	50.21	0.315	0.09	15.543
0.09	247.08	0.5652	6385.3	48.72	74.08	61.96	0.345	0.09	19.557
0.09	173.57	0.5652	6385.8	69.36	52.04	45.06	0.375	0.09	15.472
0.12	1		#N/A	#N/A	#N/A	#N/A	0.18	0.12	#N/A
0.12	216.23	0.7536	6384.6	55.66	86.44	62.87	0.21	0.12	37.501
0.12	243.47	0.7536	6384.7	49.44	97.32	82.8	0.24	0.12	17.538
0.12	181.21	0.7536	6385.3	66.43	72.44	63.38	0.27	0.12	14.286
0.12	282.16	0.7536	6385.1	42.66	112.8	102.2	0.3	0.12	10.376
0.12	246.87	0.7536	6385.3	48.76	98.69	94.18	0.33	0.12	4.7793

3h(left)

X/m	resistivity	K	U	I	K*U/I		Y/m	X/m	(G-H)/H*100
0.03	631.42	0.1884	6384.3	38.12	31.55	17.93	0.045	0.03	75.955
0.03	541.86	0.1884	6384.7	44.43	27.07	17.56	0.075	0.03	54.134
0.03	312.9	0.1884	6385.3	76.94	15.64	12.65	0.105	0.03	23.596
0.03	477.26	0.1884	6384.9	50.44	23.85	22.06	0.135	0.03	8.103
0.03	478.08	0.1884	6385	50.35	23.89	21.09	0.165	0.03	13.282
0.03	407.66	0.1884	6385.2	59.05	20.37	18.26	0.195	0.03	11.564
0.03	484.75	0.1884	6385.2	49.66	24.22	19.85	0.225	0.03	22.064
0.03	360.31	0.1884	6385.7	66.82	18	15.85	0.255	0.03	13.56
0.03	314.53	0.1884	6385.6	76.54	15.72	13.11	0.285	0.03	19.923
0.03	364.38	0.1884	6385.8	66.07	18.21	16.04	0.315	0.03	13.503
0.03	337.81	0.1884	6385.7	71.27	16.88	14.4	0.345	0.03	17.255
0.03	320.49	0.1884	6385.7	75.12	16.02	13.62	0.375	0.03	17.567
0.03	143.78	0.1884	6385.9	83.72	14.37	11.84	0.405	0.03	21.376
0.03	154.43	0.1884	6385.8	77.95	15.43	12.68	0.435	0.03	21.714
0.03	146.27	0.1884	6385.9	82.3	14.62	12.5	0.465	0.03	16.926
0.06	805.76	0.3768	6384.6	44.81	53.69	31.24	0.09	0.06	71.86
0.06	754.67	0.3768	6384.9	47.85	50.28	30.28	0.12	0.06	66.021
0.06	507.23	0.3768	6385.1	71.19	33.8	27.61	0.15	0.06	22.386
0.06	723.57	0.3768	6384.9	49.9	48.21	42.06	0.18	0.06	14.621
0.06	742.73	0.3768	6385	48.62	49.48	43.84	0.21	0.06	12.868
0.06	606.08	0.3768	6385.5	59.58	40.38	36.91	0.24	0.06	9.4159
0.06	731.07	0.3768	6385.2	49.4	48.7	41.56	0.27	0.06	17.199
0.06	637.24	0.3768	6385.5	56.67	42.46	37.34	0.3	0.06	13.704
0.06	515.59	0.3768	6385.7	70.04	34.35	29.32	0.33	0.06	17.171
0.06	159.69	0.3768	6385.7	75.38	31.92	25.67	0.36	0.06	24.328
0.06	180.84	0.3768	6385.4	66.57	36.14	30.06	0.39	0.06	20.231
0.06	129.42	0.3768	6385.9	93.02	25.87	22.29	0.42	0.06	16.032
0.09	1099.19	0.5652	6384.6	43.8	82.39	44.33	0.135	0.09	85.835

0.09	1062.75	0.5652	6384.8	45.3	79.66	47.54	0.165	0.09	67.579
0.09	667.69	0.5652	6385.5	72.11	50.05	41.92	0.195	0.09	19.384
0.09	980.9	0.5652	6385.2	49.09	73.52	66.33	0.225	0.09	10.832
0.09	1117.58	0.5652	6385.2	43.08	83.77	74.37	0.255	0.09	12.642
0.09	878.02	0.5652	6385.7	54.84	65.81	60.48	0.285	0.09	8.8212
0.09	187.88	0.5652	6385.6	64.07	56.33	50.21	0.315	0.09	12.201
0.09	239.06	0.5652	6385.1	50.35	71.68	61.96	0.345	0.09	15.683
0.09	172.58	0.5652	6385.5	69.75	51.74	45.06	0.375	0.09	14.821
0.12	1		#N/A	#N/A	#N/A	#N/A	0.18	0.12	#N/A
0.12	291.08	0.7536	6384.1	41.35	116.4	62.87	0.21	0.12	85.074
0.12	317.42	0.7536	6384.2	37.92	126.9	82.8	0.24	0.12	53.236
0.12	179.63	0.7536	6385.1	67.01	71.81	63.38	0.27	0.12	13.295
0.12	280.77	0.7536	6384.9	42.87	112.2	102.2	0.3	0.12	9.8309
0.12	258.09	0.7536	6384.8	46.64	103.2	94.18	0.33	0.12	9.5343

5h(left)

X/m	resistivity	K	U	I	K*U/I		Y/m	X/m	(G-H)/H*100
0.03	730.04	0.1884	6384	32.97	36.48	17.93	0.045	0.03	103.43
0.03	604.79	0.1884	6384.4	39.8	30.22	17.56	0.075	0.03	72.057
0.03	317.04	0.1884	6385.3	75.93	15.84	12.65	0.105	0.03	25.239
0.03	477.94	0.1884	6384.6	50.37	23.88	22.06	0.135	0.03	8.2492
0.03	483.68	0.1884	6384.8	49.77	24.17	21.09	0.165	0.03	14.597
0.03	402.98	0.1884	6385	59.74	20.14	18.26	0.195	0.03	10.27
0.03	478.88	0.1884	6384.9	50.27	23.93	19.85	0.225	0.03	20.578
0.03	356.39	0.1884	6385.4	67.55	17.81	15.85	0.255	0.03	12.327
0.03	316.77	0.1884	6385.4	76	15.83	13.11	0.285	0.03	20.771
0.03	362.3	0.1884	6385.5	66.45	18.1	16.04	0.315	0.03	12.848
0.03	337.21	0.1884	6385.5	71.39	16.85	14.4	0.345	0.03	17.056
0.03	321.47	0.1884	6385.6	74.89	16.06	13.62	0.375	0.03	17.926
0.03	146	0.1884	6385.7	82.45	14.59	11.84	0.405	0.03	23.241
0.03	156.65	0.1884	6385.6	76.85	15.65	12.68	0.435	0.03	23.453
0.03	146.06	0.1884	6385.8	82.42	14.6	12.5	0.465	0.03	16.753
0.06	970.9	0.3768	6384.3	37.19	64.68	31.24	0.09	0.06	107.07
0.06	861.23	0.3768	6384.6	41.93	57.37	30.28	0.12	0.06	89.452
0.06	515.67	0.3768	6385	70.02	34.36	27.61	0.15	0.06	24.428
0.06	736.99	0.3768	6384.7	48.99	49.11	42.06	0.18	0.06	16.747
0.06	752.17	0.3768	6384.9	48.01	50.11	43.84	0.21	0.06	14.299
0.06	597.91	0.3768	6385.5	60.4	39.84	36.91	0.24	0.06	7.9295
0.06	713.35	0.3768	6385.1	50.62	47.53	41.56	0.27	0.06	14.372
0.06	631.28	0.3768	6385.3	57.2	42.06	37.34	0.3	0.06	12.647
0.06	514.45	0.3768	6385.7	70.2	34.28	29.32	0.33	0.06	16.903
0.06	161.62	0.3768	6385.4	74.48	32.3	25.67	0.36	0.06	25.824
0.06	182.43	0.3768	6385.3	65.98	36.47	30.06	0.39	0.06	21.306
0.06	128.98	0.3768	6385.7	93.33	25.78	22.29	0.42	0.06	15.643
0.09	1344.9	0.5652	6384.3	35.8	100.8	44.33	0.135	0.09	127.35
0.09	1231.92	0.5652	6384.4	39.08	92.34	47.54	0.165	0.09	94.238
0.09	677.16	0.5652	6385.2	71.1	50.76	41.92	0.195	0.09	21.074
0.09	988.94	0.5652	6385	48.69	74.12	66.33	0.225	0.09	11.739
0.09	1128.01	0.5652	6385	42.68	84.56	74.37	0.255	0.09	13.695
0.09	864.22	0.5652	6385.5	55.72	64.77	60.48	0.285	0.09	7.1003

0.09	185.02	0.5652	6385.4	65.06	55.47	50.21	0.315	0.09	10.49
0.09	232.86	0.5652	6384.8	51.69	69.81	61.96	0.345	0.09	12.678
0.09	171.11	0.5652	6385.3	70.35	51.3	45.06	0.375	0.09	13.837
0.12	1		#N/A	#N/A	#N/A	#N/A	0.18	0.12	#N/A
0.12	346.2	0.7536	6384	34.76	138.4	62.87	0.21	0.12	120.16
0.12	354.58	0.7536	6350.6	33.77	141.7	82.8	0.24	0.12	71.161
0.12	179.02	0.7536	6385	67.24	71.56	63.38	0.27	0.12	12.905
0.12	276.55	0.7536	6384.7	43.52	110.6	102.2	0.3	0.12	8.1873
0.12	259.01	0.7536	6384.6	46.47	103.5	94.18	0.33	0.12	9.9304

15h(left)

X/m	resistivity	K	U	I	K*U/I		Y/m	X/m	(G-H)/H*100
0.03	803.38	0.1884	6383.6	29.96	40.14	17.93	0.045	0.03	123.85
0.03	729.89	0.1884	6383.9	32.98	36.47	17.56	0.075	0.03	107.62
0.03	336.61	0.1884	6384.9	71.52	16.82	12.65	0.105	0.03	32.953
0.03	502.75	0.1884	6384.4	47.88	25.12	22.06	0.135	0.03	13.874
0.03	556.37	0.1884	6384.3	43.26	27.8	21.09	0.165	0.03	31.833
0.03	417.59	0.1884	6384.8	57.65	20.87	18.26	0.195	0.03	14.265
0.03	489.87	0.1884	6384.5	49.14	24.48	19.85	0.225	0.03	23.343
0.03	373.27	0.1884	6384.9	64.49	18.65	15.85	0.255	0.03	17.648
0.03	310.83	0.1884	6385	77.45	15.53	13.11	0.285	0.03	18.502
0.03	349.8	0.1884	6385	68.82	17.48	16.04	0.315	0.03	8.9541
0.03	323.34	0.1884	6385.2	74.45	16.16	14.4	0.345	0.03	12.239
0.03	311.52	0.1884	6385.3	77.28	15.57	13.62	0.375	0.03	14.273
0.03	138.93	0.1884	6385.2	86.64	13.88	11.84	0.405	0.03	17.274
0.03	156.54	0.1884	6385.2	76.89	15.65	12.68	0.435	0.03	23.38
0.03	141.89	0.1884	6385.5	84.84	14.18	12.5	0.465	0.03	13.417
0.06	1045.85	0.3768	6383.9	34.52	69.68	31.24	0.09	0.06	123.07
0.06	1032.73	0.3768	6384	34.96	68.81	30.28	0.12	0.06	127.2
0.06	532.9	0.3768	6384.9	67.76	35.5	27.61	0.15	0.06	28.575
0.06	787.64	0.3768	6384.3	45.84	52.48	42.06	0.18	0.06	24.761
0.06	870.73	0.3768	6384.3	41.47	58.01	43.84	0.21	0.06	32.312
0.06	619.59	0.3768	6384.8	58.28	41.28	36.91	0.24	0.06	11.844
0.06	721.59	0.3768	6384.7	50.04	48.08	41.56	0.27	0.06	15.69
0.06	647.1	0.3768	6384.9	55.8	43.12	37.34	0.3	0.06	15.467
0.06	510.83	0.3768	6385.2	70.69	34.04	29.32	0.33	0.06	16.084
0.06	158.97	0.3768	6385	75.72	31.77	25.67	0.36	0.06	23.755
0.06	181.15	0.3768	6384.8	66.44	36.21	30.06	0.39	0.06	20.455
0.06	125.19	0.3768	6385.4	96.15	25.02	22.29	0.42	0.06	12.246
0.09	1462.61	0.5652	6383.9	32.91	109.6	44.33	0.135	0.09	147.3
0.09	1504.55	0.5652	6383.9	32	112.8	47.54	0.165	0.09	137.19
0.09	699.53	0.5652	6385.1	68.83	52.43	41.92	0.195	0.09	25.064
0.09	1042.36	0.5652	6384.5	46.19	78.12	66.33	0.225	0.09	17.777
0.09	1279.81	0.5652	6384.3	37.62	95.92	74.37	0.255	0.09	28.973
0.09	899.68	0.5652	6384.9	53.51	67.44	60.48	0.285	0.09	11.512
0.09	196.22	0.5652	6385	61.34	58.83	50.21	0.315	0.09	17.185
0.09	231.56	0.5652	6384.7	51.98	69.42	61.96	0.345	0.09	12.048
0.09	177.07	0.5652	6384.9	67.97	53.09	45.06	0.375	0.09	17.816
0.12	1		#N/A	#N/A	#N/A	#N/A	0.18	0.12	#N/A
0.12	384.9	0.7536	6383.5	31.27	153.8	62.87	0.21	0.12	144.71

0.12	412.47	0.7536	5918.3	27.05	164.9	82.8	0.24	0.12	99.137
0.12	192.3	0.7536	6384.5	62.59	76.87	63.38	0.27	0.12	21.283
0.12	291.83	0.7536	6384.5	41.24	116.7	102.2	0.3	0.12	14.166
0.12	300.72	0.7536	6384.2	40.02	120.2	94.18	0.33	0.12	27.641

20h(left)

X/m	resistivity	K	U	I	K*U/I		Y/m	X/m	(G-H)/H*100
0.03	929.91	0.1884	6377	25.86	46.46	17.93	0.045	0.03	159.08
0.03	799.95	0.1884	6384.7	30.09	39.98	17.56	0.075	0.03	127.59
0.03	375.66	0.1884	6385.8	64.09	18.77	12.65	0.105	0.03	48.389
0.03	679.72	0.1884	6384.9	35.42	33.96	22.06	0.135	0.03	53.945
0.03	579.49	0.1884	6385.4	41.55	28.95	21.09	0.165	0.03	37.281
0.03	440.98	0.1884	6385.9	54.6	22.03	18.26	0.195	0.03	20.668
0.03	544.13	0.1884	6385.3	44.24	27.19	19.85	0.225	0.03	37.021
0.03	402.22	0.1884	6385.7	59.86	20.1	15.85	0.255	0.03	26.764
0.03	336.87	0.1884	6385.7	71.47	16.83	13.11	0.285	0.03	28.432
0.03	493.58	0.1884	6385.5	48.78	24.66	16.04	0.315	0.03	53.726
0.03	312.5	0.1884	6386	77.04	15.62	14.4	0.345	0.03	8.4794
0.03	302.66	0.1884	6386	79.55	15.12	13.62	0.375	0.03	11.024
0.03	131.78	0.1884	6386.1	91.35	13.17	11.84	0.405	0.03	11.243
0.03	156.73	0.1884	6385.9	76.81	15.66	12.68	0.435	0.03	23.523
0.03	136.04	0.1884	6386.2	88.5	13.59	12.5	0.465	0.03	8.7383
0.06	1452.83	0.3768	6384.5	24.86	96.77	31.24	0.09	0.06	209.78
0.06	1107.28	0.3768	6384.9	32.61	73.78	30.28	0.12	0.06	143.61
0.06	553.82	0.3768	6386.1	65.21	36.9	27.61	0.15	0.06	33.629
0.06	830.79	0.3768	6385.4	43.47	55.35	42.06	0.18	0.06	31.585
0.06	903.21	0.3768	6385.4	39.98	60.18	43.84	0.21	0.06	37.267
0.06	654.24	0.3768	6385.7	55.2	43.59	36.91	0.24	0.06	18.102
0.06	798.51	0.3768	6385.4	45.23	53.19	41.56	0.27	0.06	28.007
0.06	681.15	0.3768	6385.6	53.02	45.38	37.34	0.3	0.06	21.534
0.06	556.77	0.3768	6385.9	64.86	37.1	29.32	0.33	0.06	26.533
0.06	171.06	0.3768	6386	70.37	34.19	25.67	0.36	0.06	33.184
0.06	252.54	0.3768	6385.3	47.67	50.47	30.06	0.39	0.06	67.898
0.06	123.64	0.3768	6386.1	97.37	24.71	22.29	0.42	0.06	10.851
0.09	1671.06	0.5652	6384.8	28.81	125.3	44.33	0.135	0.09	182.54
0.09	1567.66	0.5652	6384.9	30.71	117.5	47.54	0.165	0.09	147.2
0.09	727.02	0.5652	6386.2	66.24	54.49	41.92	0.195	0.09	29.978
0.09	1083.48	0.5652	6385.6	44.44	81.21	66.33	0.225	0.09	22.437
0.09	1314.68	0.5652	6385.3	36.63	98.52	74.37	0.255	0.09	32.479
0.09	949.74	0.5652	6385.7	50.7	71.19	60.48	0.285	0.09	17.708
0.09	219.54	0.5652	6385.8	54.84	65.81	50.21	0.315	0.09	31.09
0.09	337.51	0.5652	6385.3	35.67	101.2	61.96	0.345	0.09	63.296
0.09	190.63	0.5652	6385.9	63.15	57.15	45.06	0.375	0.09	26.829
0.12	1		#N/A	#N/A	#N/A	#N/A	0.18	0.12	#N/A
0.12	419.95	0.7536	6199.8	27.83	167.9	62.87	0.21	0.12	167.04
0.12	424.07	0.7536	5722.3	25.44	169.5	82.8	0.24	0.12	104.73
0.12	200.8	0.7536	6385.4	59.95	80.27	63.38	0.27	0.12	26.642
0.12	315.55	0.7536	6385.3	38.15	126.1	102.2	0.3	0.12	23.428
0.12	316.86	0.7536	6385.1	37.99	126.7	94.18	0.33	0.12	34.48

36h(left)

X/m	resistivity	K	U	I	K*U/I		Y/m	X/m	(G-H)/H*100
0.03	735.66	0.1884	6383.5	32.72	36.76	17.93	0.045	0.03	104.97
0.03	744.7	0.1884	6383.8	32.32	37.21	17.56	0.075	0.03	111.86
0.03	354.72	0.1884	6385.1	67.87	17.72	12.65	0.105	0.03	40.108
0.03	908.82	0.1884	4931.6	20.46	45.41	22.06	0.135	0.03	105.85
0.03	606.2	0.1884	6384.5	39.71	30.29	21.09	0.165	0.03	43.623
0.03	443.27	0.1884	6385	54.31	22.15	18.26	0.195	0.03	21.295
0.03	699.95	0.1884	6384.6	34.39	34.98	19.85	0.225	0.03	76.246
0.03	437.63	0.1884	6385.4	55.01	21.87	15.85	0.255	0.03	37.934
0.03	497.14	0.1884	6384.8	48.42	24.84	13.11	0.285	0.03	89.543
0.03	903.87	0.1884	4905.2	20.46	45.17	16.04	0.315	0.03	181.55
0.03	353.74	0.1884	6385.4	68.06	17.68	14.4	0.345	0.03	22.781
0.03	327.42	0.1884	6386.2	73.54	16.36	13.62	0.375	0.03	20.101
0.03	214.06	0.1884	6385.1	56.23	21.39	11.84	0.405	0.03	80.692
0.03	247.53	0.1884	6384.9	48.63	24.74	12.68	0.435	0.03	95.072
0.03	140.53	0.1884	6386.4	85.67	14.04	12.5	0.465	0.03	12.335
0.06	1783.67	0.3768	4995.8	15.84	118.8	31.24	0.09	0.06	280.42
0.06	1117.19	0.3768	6384.1	32.32	74.43	30.28	0.12	0.06	145.76
0.06	559.86	0.3768	6385.3	64.5	37.3	27.61	0.15	0.06	35.083
0.06	959.84	0.3768	6384.3	37.62	63.95	42.06	0.18	0.06	52.023
0.06	932.2	0.3768	6384.8	38.74	62.1	43.84	0.21	0.06	41.647
0.06	839.37	0.3768	6385	43.02	55.92	36.91	0.24	0.06	51.522
0.06	964.14	0.3768	6384.5	37.45	64.24	41.56	0.27	0.06	54.58
0.06	672.93	0.3768	6385.4	53.66	44.84	37.34	0.3	0.06	20.08
0.06	588.43	0.3768	6385.7	61.37	39.21	29.32	0.33	0.06	33.723
0.06	184.97	0.3768	6385.6	65.08	36.97	25.67	0.36	0.06	44.002
0.06	492.37	0.3768	4991.1	19.11	98.41	30.06	0.39	0.06	227.38
0.06	165.86	0.3768	6385.1	72.57	33.15	22.29	0.42	0.06	48.712
0.09	1497.61	0.5652	6384	32.15	112.2	44.33	0.135	0.09	153.15
0.09	1587.27	0.5652	6384.1	30.33	119	47.54	0.165	0.09	150.26
0.09	975.02	0.5652	6385.2	49.38	73.08	41.92	0.195	0.09	74.329
0.09	1172.42	0.5652	6385.1	41.07	87.87	66.33	0.225	0.09	32.473
0.09	1253.54	0.5652	6384.9	38.41	93.95	74.37	0.255	0.09	26.331
0.09	919.19	0.5652	6385.4	52.38	68.9	60.48	0.285	0.09	13.927
0.09	209.18	0.5652	6385.2	57.54	62.72	50.21	0.315	0.09	24.927
0.09	497.71	0.5652	4995.3	18.92	149.2	61.96	0.345	0.09	140.85
0.09	210.54	0.5652	6385.3	57.17	63.13	45.06	0.375	0.09	40.081
0.12	1		#N/A	#N/A	#N/A	#N/A	0.18	0.12	#N/A
0.12	293.48	0.7536	6383.5	41.01	117.3	62.87	0.21	0.12	86.591
0.12	409.96	0.7536	5964.8	27.43	163.9	82.8	0.24	0.12	97.921
0.12	173.24	0.7536	6384.7	69.48	69.25	63.38	0.27	0.12	9.2601
0.12	307.5	0.7536	6384.3	39.14	122.9	102.2	0.3	0.12	20.287
0.12	304.02	0.7536	6384.4	39.59	121.5	94.18	0.33	0.12	29.031

48h(left)

X/m	resistivity	K	U	I	K*U/I		Y/m	X/m	(G-H)/H*100
0.03	806.09	0.1884	6382.7	29.86	40.27	17.93	0.045	0.03	124.57
0.03	633.14	0.1884	6383.1	38.01	31.64	17.56	0.075	0.03	80.122

0.03	357.48	0.1884	6384	67.33	17.86	12.65	0.105	0.03	41.209
0.03	791.38	0.1884	6383	30.41	39.54	22.06	0.135	0.03	79.255
0.03	553.85	0.1884	6383.9	43.46	27.67	21.09	0.165	0.03	31.217
0.03	439.71	0.1884	6384.3	54.74	21.97	18.26	0.195	0.03	20.33
0.03	802.56	0.1884	6383.6	29.99	40.1	19.85	0.225	0.03	102.07
0.03	445.37	0.1884	6384.6	54.05	22.25	15.85	0.255	0.03	40.366
0.03	524.49	0.1884	6384	45.89	26.21	13.11	0.285	0.03	99.969
0.03	761.79	0.1884	6383.6	31.6	38.06	16.04	0.315	0.03	137.23
0.03	364.48	0.1884	6384.9	66.05	18.21	14.4	0.345	0.03	26.507
0.03	344.91	0.1884	6385.3	69.8	17.23	13.62	0.375	0.03	26.52
0.03	227.72	0.1884	6384.4	52.85	22.76	11.84	0.405	0.03	92.227
0.03	301.11	0.1884	6383.9	39.97	30.09	12.68	0.435	0.03	137.3
0.03	145.23	0.1884	6385.7	82.89	14.51	12.5	0.465	0.03	16.09
0.06	1648.13	0.3768	6382.5	21.9	109.8	31.24	0.09	0.06	251.53
0.06	930.23	0.3768	6383.5	38.81	61.98	30.28	0.12	0.06	104.65
0.06	566.21	0.3768	6384.3	63.77	37.72	27.61	0.15	0.06	36.609
0.06	1069.51	0.3768	6383.5	33.76	71.25	42.06	0.18	0.06	69.382
0.06	841.74	0.3768	6384.2	42.89	56.09	43.84	0.21	0.06	27.929
0.06	844.49	0.3768	6384.2	42.75	56.27	36.91	0.24	0.06	52.46
0.06	963.23	0.3768	6384	37.48	64.18	41.56	0.27	0.06	54.442
0.06	681.86	0.3768	6384.5	52.95	45.43	37.34	0.3	0.06	21.674
0.06	610.99	0.3768	6384.9	59.1	40.71	29.32	0.33	0.06	38.843
0.06	194.84	0.3768	6385	61.78	38.94	25.67	0.36	0.06	51.678
0.06	476.34	0.3768	6383.4	25.27	95.18	30.06	0.39	0.06	216.64
0.06	172.11	0.3768	6384.3	69.93	34.4	22.29	0.42	0.06	54.307
0.09	1877.8	0.5652	6382.9	25.63	140.8	44.33	0.135	0.09	217.5
0.09	1235.16	0.5652	6383.7	38.97	92.59	47.54	0.165	0.09	94.764
0.09	994.25	0.5652	6384.4	48.42	74.52	41.92	0.195	0.09	77.762
0.09	1129.61	0.5652	6384.3	42.62	84.66	66.33	0.225	0.09	27.639
0.09	1108	0.5652	6384.4	43.45	83.05	74.37	0.255	0.09	11.67
0.09	896.43	0.5652	6384.9	53.71	67.19	60.48	0.285	0.09	11.097
0.09	208.95	0.5652	6384.5	57.6	62.65	50.21	0.315	0.09	24.784
0.09	437.57	0.5652	6383.4	27.5	131.2	61.96	0.345	0.09	111.75
0.09	216.86	0.5652	6384.5	55.5	65.02	45.06	0.375	0.09	44.279
0.12	1		#N/A	#N/A	#N/A	#N/A	0.18	0.12	#N/A
0.12	319.77	0.7536	6382.7	37.63	127.8	62.87	0.21	0.12	103.32
0.12	354.91	0.7536	6327.4	33.61	141.9	82.8	0.24	0.12	71.348
0.12	163.58	0.7536	6383.8	73.57	65.39	63.38	0.27	0.12	3.1711
0.12	294.99	0.7536	6383.5	40.8	117.9	102.2	0.3	0.12	15.378
0.12	279.66	0.7536	6383.5	43.03	111.8	94.18	0.33	0.12	18.7

1h(right)

Y/m	X/m	resistivity	0h resistivity	K	U	I	K*U/I	R	((K*U/I)-R)/R*100
0.45	-0.3	380.93	336.88	0.1884	6384	71.45	16.834	16.17	4.0953
0.75	-0.3	400.97	258.07	0.1884	6385	93.28	12.896	12.9	-0.024
1.05	-0.3	386.23	349.65	0.1884	6385	68.85	17.472	16.84	3.7286
1.35	-0.3	338.71	451.52	0.1884	6384	53.31	22.563	22.82	-1.115

1.65	-0.3	316.96	552	0.1884	6384	43.61	27.58	26.39	4.5022
1.95	-0.3	339.7	380.19	0.1884	6385	63.31	18.999	19.14	-0.718
2.25	-0.3	279.47	373.12	0.1884	6385	64.51	18.646	19.03	-2.022
2.55	-0.3	306.34	283.71	0.1884	6385	84.85	14.177	14.46	-1.924
2.85	-0.3	256.48	312.68	0.1884	6385	76.99	15.625	15.15	3.152
3.15	-0.3	309.41	379.89	0.1884	6385	63.37	18.983	19.1	-0.593
3.45	-0.3	262.65	280.55	0.1884	6385	85.81	14.019	14.35	-2.276
3.75	-0.3	307.5	305.06	0.1884	6385	78.91	15.244	15.59	-2.235
4.05	-0.3	113.96	120.61	0.1884	6385	99.81	12.053	12.33	-2.245
4.35	-0.3	94.08	123.5	0.1884	6386	97.47	12.343	12.73	-3.047
4.65	-0.3	70.17	126.27	0.1884	6385	95.33	12.619	12.93	-2.426
0.9	-0.6	572.71	540.89	0.3768	6385	66.76	36.037	34.68	3.919
1.2	-0.6	582.72	425.82	0.3768	6385	84.8	28.372	28.6	-0.804
1.5	-0.6	577.88	490.39	0.3768	6385	73.63	32.675	32.8	-0.382
1.8	-0.6	403.66	544.85	0.3768	6385	66.27	36.302	37.4	-2.948
2.1	-0.6	358.03	882.24	0.3768	6384	40.92	58.786	55.9	5.1651
2.4	-0.6	413.31	583.4	0.3768	6385	61.89	38.871	39.22	-0.896
2.7	-0.6	497.24	605.92	0.3768	6385	59.59	40.372	40.93	-1.369
3	-0.6	461.58	438.97	0.3768	6385	82.26	29.249	29.72	-1.584
3.3	-0.6	498.25	554.41	0.3768	6385	65.13	36.94	36.1	2.3392
3.6	-0.6	159.3	152.23	0.3768	6385	79.07	30.427	29.2	4.1935
3.9	-0.6	124.27	174.15	0.3768	6385	69.11	34.81	35.16	-1.004
4.2	-0.6	128.08	133.74	0.3768	6386	90.01	26.731	27.44	-2.579
1.35	-0.9	634.37	551.22	0.5652	6385	87.35	41.317	40.16	2.8693
1.65	-0.9	639.61	616.29	0.5652	6385	78.12	46.195	46.59	-0.837
1.95	-0.9	646.28	673.85	0.5652	6385	71.45	50.506	50.9	-0.775
2.25	-0.9	626.35	781.33	0.5652	6385	61.62	58.564	60.1	-2.552
2.55	-0.9	484.45	1226.8	0.5652	6384	39.24	91.955	86.61	6.1737
2.85	-0.9	705.7	900.78	0.5652	6385	53.45	67.516	67.9	-0.571
3.15	-0.9	162.86	202.87	0.5652	6384	59.33	60.821	58.58	3.8178
3.45	-0.9	172.37	235.43	0.5652	6385	51.12	70.59	70.66	-0.105
3.75	-0.9	189.41	128.55	0.5652	6385	93.64	38.541	39.31	-1.947
2.1	-1.2	161.93	130.44	0.7536	6384	92.26	52.145	50.1	4.0742
2.4	-1.2	154.56	250.52	0.7536	6384	48.04	100.14	92.38	8.3999
2.7	-1.2	184.68	189.87	0.7536	6385	63.39	75.903	75.14	1.0201
3	-1.2	164.6	214.01	0.7536	6385	56.24	85.552	87.22	-1.911
3.3	-1.2	161.55	256.14	0.7536	6385	46.99	102.39	97.45	5.0731

2h(right)

Y/m	X/m	resistivity	K	U	I	K*U/I	R	(H-R)/R*100
	0.03	397.36	0.1884	6385.1	60.58	19.86	16.172	22.79
	0.03	328.67	0.1884	6385.7	73.25	16.42	12.9	27.32
	0.03	452.22	0.1884	6385.5	53.24	22.6	16.844	34.15
	0.03	528.45	0.1884	6385.2	45.56	26.4	22.817	15.72
	0.03	641.35	0.1884	6384.9	37.54	32.04	26.392	21.42
	0.03	426.97	0.1884	6385.5	56.39	21.33	19.137	11.48
	0.03	417.28	0.1884	6385.7	57.7	20.85	19.031	9.56
	0.03	325.23	0.1884	6385.8	74.03	16.25	14.455	12.42
	0.03	394.9	0.1884	6386	60.97	19.73	15.147	30.27

	0.03	462.71	0.1884	6385.8	52.03	23.12	19.096	21.09
	0.03	326.5	0.1884	6386	73.74	16.32	14.346	13.73
	0.03	341.39	0.1884	6385.9	70.52	17.06	15.593	9.41
	0.03	136.85	0.1884	6386.3	87.98	13.68	12.33	10.91
	0.03	142.83	0.1884	6386.2	84.28	14.28	12.731	12.14
	0.03	146.42	0.1884	6386.1	82.22	14.63	12.933	13.15
	0.06	680.49	0.3768	6385.4	53.07	45.34	34.678	30.73
	0.06	539.11	0.3768	6385.7	66.99	35.92	28.602	25.58
	0.06	592.89	0.3768	6385.6	60.91	39.5	32.8	20.43
	0.06	610.91	0.3768	6385.5	59.11	40.7	37.405	8.822
	0.06	1011.81	0.3768	6385	35.69	67.41	55.899	20.59
	0.06	652.9	0.3768	6385.7	55.31	43.5	39.223	10.91
	0.06	678.74	0.3768	6385.7	53.21	45.22	40.933	10.47
	0.06	503.51	0.3768	6386.1	71.73	33.55	29.72	12.88
	0.06	682.13	0.3768	6385.9	52.94	45.45	36.096	25.92
	0.06	193.31	0.3768	6385.9	62.28	38.64	29.202	32.3
	0.06	211.35	0.3768	6385.6	56.96	42.24	35.163	20.13
	0.06	156.11	0.3768	6386.2	77.12	31.2	27.439	13.72
	0.09	666.97	0.5652	6385.9	72.2	49.99	40.165	24.46
	0.09	770.16	0.5652	6385.8	62.52	57.73	46.585	23.92
	0.09	810.73	0.5652	6385.6	59.39	60.77	50.9	19.39
	0.09	878.91	0.5652	6385.5	54.78	65.88	60.098	9.626
	0.09	1394.2	0.5652	6385.2	34.54	104.5	86.608	20.64
	0.09	995.77	0.5652	6385.8	48.36	74.63	67.904	9.91
	0.09	246.81	0.5652	6385.2	48.77	74	58.584	26.31
	0.09	274.17	0.5652	6385.3	43.91	82.19	70.664	16.31
	0.09	149.12	0.5652	6386.2	80.74	44.7	39.306	13.73
	0.12	1		#N/A	#N/A	#N/A	#N/A	#N/A
	0.12	158.42	0.7536	6384.9	75.98	63.33	50.104	26.39
	0.12	309.15	0.7536	6384.5	38.93	123.6	92.381	33.78
	0.12	224.28	0.7536	6385.5	53.67	89.66	75.136	19.33
	0.12	235.84	0.7536	6385.5	51.04	94.28	87.219	8.096
	0.12	298.03	0.7536	6385.5	40.39	119.1	97.45	22.26

5h(right)

Y/m	X/m	resistivity	K	U	I	K*U/I	R	(H-R)/R*100
	0.03	410.66	0.1884	6384.7	58.62	20.52	16.172	26.89
	0.03	333.63	0.1884	6385.3	72.16	16.67	12.9	29.24
	0.03	510.45	0.1884	6384.9	47.16	25.51	16.844	51.43
	0.03	554.71	0.1884	6384.7	43.4	27.72	22.817	21.47
	0.03	659.9	0.1884	6384.5	36.48	32.97	26.392	24.93
	0.03	432.12	0.1884	6385.2	55.71	21.59	19.137	12.84
	0.03	416.17	0.1884	6385.2	57.85	20.79	19.031	9.269
	0.03	322.88	0.1884	6385.5	74.56	16.14	14.455	11.62
	0.03	446.37	0.1884	6385.3	53.93	22.31	15.147	47.26
	0.03	485.05	0.1884	6385.3	49.63	24.24	19.096	26.93
	0.03	326.28	0.1884	6385.6	73.79	16.3	14.346	13.65
	0.03	337.5	0.1884	6385.5	71.33	16.87	15.593	8.162
	0.03	135.26	0.1884	6385.9	89	13.52	12.33	9.635
	0.03	140.4	0.1884	6385.9	85.74	14.03	12.731	10.22

	0.03	145.43	0.1884	6385.8	82.77	14.54	12.933	12.39
	0.06	736.32	0.3768	6384.9	49.04	49.06	34.678	41.47
	0.06	555	0.3768	6385.4	65.07	36.98	28.602	29.27
	0.06	603.93	0.3768	6385.1	59.79	40.24	32.8	22.68
	0.06	613.9	0.3768	6385.1	58.82	40.9	37.405	9.352
	0.06	1032.56	0.3768	6384.6	34.97	68.79	55.899	23.07
	0.06	655.57	0.3768	6385.1	55.08	43.68	39.223	11.36
	0.06	670.94	0.3768	6385.2	53.82	44.7	40.933	9.212
	0.06	493.37	0.3768	6385.9	73.2	32.87	29.72	10.6
	0.06	762.98	0.3768	6385.4	47.33	50.84	36.096	40.83
	0.06	218.88	0.3768	6385.3	55	43.75	29.202	49.8
	0.06	223.11	0.3768	6385.2	53.95	44.6	35.163	26.82
	0.06	158.29	0.3768	6385.9	76.05	31.64	27.439	15.31
	0.09	691.23	0.5652	6385.3	69.66	51.81	40.165	28.99
	0.09	787.71	0.5652	6385.2	61.12	59.05	46.585	26.75
	0.09	822.99	0.5652	6385.1	58.5	61.69	50.9	21.2
	0.09	875.51	0.5652	6385.1	54.99	65.63	60.098	9.201
	0.09	1424.52	0.5652	6384.7	33.8	106.8	86.608	23.27
	0.09	995.84	0.5652	6385.4	48.35	74.64	67.904	9.925
	0.09	277.81	0.5652	6384.7	43.33	83.28	58.584	42.16
	0.09	285.44	0.5652	6384.9	42.17	85.58	70.664	21.1
	0.09	149.52	0.5652	6385.6	80.51	44.83	39.306	14.05
	0.12	1		#N/A	#N/A	#N/A	#N/A	#N/A
	0.12	165.84	0.7536	6384.3	72.57	66.3	50.104	32.32
	0.12	320.86	0.7536	6384.2	37.51	128.3	92.381	38.84
	0.12	231.2	0.7536	6384.9	52.06	92.43	75.136	23.01
	0.12	237.11	0.7536	6385.1	50.77	94.78	87.219	8.666
	0.12	305.29	0.7536	6385	39.43	122	97.45	25.23

15h(right)

Y/m	X/m	resistivity	K	U	I	K*U/I	R	(H-R)/R*100
	0.03	506.41	0.1884	6383.9	47.53	25.3	16.172	56.47
	0.03	367.71	0.1884	6384.8	65.46	18.38	12.9	42.45
	0.03	600.74	0.1884	6384.3	40.07	30.02	16.844	78.21
	0.03	544.02	0.1884	6384.3	44.25	27.18	22.817	19.13
	0.03	719.32	0.1884	6383.9	33.46	35.95	26.392	36.2
	0.03	452.26	0.1884	6384.5	53.23	22.6	19.137	18.08
	0.03	440.28	0.1884	6384.4	54.67	22	19.031	15.61
	0.03	335.33	0.1884	6384.7	71.78	16.76	14.455	15.93
	0.03	514.43	0.1884	6384.4	46.79	25.71	15.147	69.71
	0.03	442.89	0.1884	6384.8	54.35	22.13	19.096	15.9
	0.03	316.8	0.1884	6384.9	75.99	15.83	14.346	10.35
	0.03	319.29	0.1884	6384.9	75.39	15.96	15.593	2.326
	0.03	128.42	0.1884	6385.3	93.73	12.83	12.33	4.094
	0.03	133.12	0.1884	6385.2	90.42	13.3	12.731	4.506
	0.03	141.1	0.1884	6385.1	85.3	14.1	12.933	9.043
	0.06	792.71	0.3768	6384.3	45.55	52.81	34.678	52.29
	0.06	577.4	0.3768	6384.9	62.54	38.47	28.602	34.5
	0.06	614.35	0.3768	6384.7	58.77	40.94	32.8	24.8
	0.06	645.89	0.3768	6384.5	55.9	43.04	37.405	15.05

	0.06	1120.42	0.3768	6383.8	32.22	74.66	55.899	33.55
	0.06	690.09	0.3768	6384.4	52.32	45.98	39.223	17.23
	0.06	706.47	0.3768	6384.5	51.11	47.07	40.933	14.99
	0.06	514.55	0.3768	6385	70.18	34.28	29.72	15.35
	0.06	864.93	0.3768	6384.5	41.75	57.62	36.096	59.63
	0.06	249.87	0.3768	6384.5	48.17	49.94	29.202	71.02
	0.06	205.21	0.3768	6384.4	58.65	41.02	35.163	16.65
	0.06	153.68	0.3768	6385.2	78.32	30.72	27.439	11.96
	0.09	830.23	0.5652	6384.9	57.99	62.23	40.165	54.94
	0.09	815.71	0.5652	6384.9	59.02	61.14	46.585	31.25
	0.09	839.57	0.5652	6384.6	57.34	62.93	50.9	23.64
	0.09	920.29	0.5652	6384.4	52.31	68.98	60.098	14.78
	0.09	1534.97	0.5652	6383.9	31.36	115.1	86.608	32.85
	0.09	1033.04	0.5652	6384.5	46.6	77.44	67.904	14.04
	0.09	329.8	0.5652	6384.1	36.49	98.88	58.584	68.79
	0.09	279.22	0.5652	6384.4	43.11	83.7	70.664	18.45
	0.09	156.25	0.5652	6385.2	77.03	46.85	39.306	19.19
	0.12	1		#N/A	#N/A	#N/A	#N/A	#N/A
	0.12	203.9	0.7536	6383.7	59.02	81.51	50.104	62.68
	0.12	350.32	0.7536	6383.6	34.35	140	92.381	51.6
	0.12	242.27	0.7536	6384.3	49.68	96.84	75.136	28.89
	0.12	259.1	0.7536	6384.4	46.45	103.6	87.219	18.76
	0.12	345.34	0.7536	6384.2	34.85	138.1	97.45	41.66

20h(right)

Y/m	X/m	resistivity	K	U	I	K*U/I	R	(H-R)/R*100
	0.03	566.55	0.1884	6384.9	42.49	28.31	16.172	75.06
	0.03	406.47	0.1884	6385.8	59.23	20.31	12.9	57.46
	0.03	758.89	0.1884	6385	31.72	37.92	16.844	125.1
	0.03	589.12	0.1884	6385.4	40.87	29.44	22.817	29.01
	0.03	818.56	0.1884	6385	29.41	40.9	26.392	54.98
	0.03	521.73	0.1884	6385.7	46.15	26.07	19.137	36.22
	0.03	473.05	0.1884	6385.8	50.9	23.64	19.031	24.2
	0.03	373.05	0.1884	6386	64.54	18.64	14.455	28.96
	0.03	676.14	0.1884	6385.2	35.61	33.78	15.147	123
	0.03	480.16	0.1884	6385.7	50.14	23.99	19.096	25.65
	0.03	362.14	0.1884	6385.7	66.48	18.1	14.346	26.15
	0.03	353.03	0.1884	6385.7	68.2	17.64	15.593	13.13
	0.03	146.65	0.1884	6386	82.09	14.66	12.33	18.87
	0.03	129.59	0.1884	6386.2	92.9	12.95	12.731	1.732
	0.03	135.87	0.1884	6386.1	88.6	13.58	12.933	4.999
	0.06	920.55	0.3768	6385.3	39.23	61.33	34.678	76.85
	0.06	683.54	0.3768	6385.8	52.83	45.55	28.602	59.24
	0.06	699.38	0.3768	6385.7	51.64	46.59	32.8	42.06
	0.06	673.57	0.3768	6385.8	53.62	44.87	37.405	19.97
	0.06	1201.22	0.3768	6385	30.06	80.04	55.899	43.18
	0.06	729.36	0.3768	6385.7	49.51	48.6	39.223	23.9
	0.06	748.6	0.3768	6385.6	48.24	49.88	40.933	21.85
	0.06	576.02	0.3768	6386	62.7	38.38	29.72	29.13
	0.06	1114.72	0.3768	6385.2	32.4	74.26	36.096	105.7

	0.06	348.83	0.3768	6385.4	34.51	69.72	29.202	138.7
	0.06	226.03	0.3768	6385.7	53.26	45.18	35.163	28.48
	0.06	173.34	0.3768	6385.9	69.45	34.65	27.439	26.27
	0.09	948.97	0.5652	6385.9	50.74	71.13	40.165	77.1
	0.09	853.89	0.5652	6386.3	56.4	64	46.585	37.38
	0.09	869.48	0.5652	6386.1	55.38	65.18	50.9	28.05
	0.09	951.39	0.5652	6385.9	50.61	71.32	60.098	18.67
	0.09	1632.62	0.5652	6385.2	29.49	122.4	86.608	41.3
	0.09	1076.15	0.5652	6385.7	44.75	80.65	67.904	18.77
	0.09	415.95	0.5652	6384.9	28.94	124.7	58.584	112.9
	0.09	306.1	0.5652	6385.8	39.33	91.77	70.664	29.87
	0.09	201.5	0.5652	6386.5	59.75	60.41	39.306	53.7
	0.12	1		#N/A	#N/A	#N/A	#N/A	#N/A
	0.12	223.48	0.7536	6384.7	53.86	89.33	50.104	78.3
	0.12	370.85	0.7536	6384.7	32.46	148.2	92.381	60.45
	0.12	248	0.7536	6385.5	48.54	99.14	75.136	31.94
	0.12	268.13	0.7536	6385.6	44.9	107.2	87.219	22.88
	0.12	380.35	0.7536	6385.5	31.65	152	97.45	56.02

36h(right)

Y/m	X/m	resistivity	K	U	I	K*U/I	R	(H-R)/R*100
	0.03	479.37	0.1884	6384	50.21	23.95	16.172	48.12
	0.03	393.09	0.1884	6384.6	61.24	19.64	12.9	52.27
	0.03	858.53	0.1884	6383.8	28.04	42.89	16.844	154.7
	0.03	603.06	0.1884	6384.5	39.92	30.13	22.817	32.06
	0.03	1192.26	0.1884	6383.7	20.19	59.57	26.392	125.7
	0.03	949.52	0.1884	6173.9	24.52	47.44	19.137	147.9
	0.03	753.03	0.1884	6384.5	31.97	37.62	19.031	97.7
	0.03	709.8	0.1884	6384.3	33.91	35.47	14.455	145.4
	0.03	1046.56	0.1884	6384.3	23	52.3	15.147	245.2
	0.03	682.05	0.1884	6384.6	35.3	34.08	19.096	78.44
	0.03	511.74	0.1884	6385.3	47.04	25.57	14.346	78.27
	0.03	913.5	0.1884	5956	24.58	45.65	15.593	192.8
	0.03	479.1	0.1884	6043.8	23.78	47.88	12.33	288.3
	0.03	352.88	0.1884	6384.3	34.11	35.26	12.731	177
	0.03	249.52	0.1884	6385.1	48.24	24.94	12.933	92.82
	0.06	910.9	0.3768	6384.2	39.64	60.68	34.678	74.99
	0.06	897.95	0.3768	6384.2	40.21	59.83	28.602	109.2
	0.06	1325.96	0.3768	6383.4	27.23	88.33	32.8	169.3
	0.06	1037.41	0.3768	6384.4	34.81	69.11	37.405	84.76
	0.06	2207.66	0.3768	6383.6	16.36	147	55.899	163
	0.06	982.3	0.3768	6384.6	36.76	65.44	39.223	66.85
	0.06	940.96	0.3768	6385.1	38.38	62.69	40.933	53.15
	0.06	610.21	0.3768	6385.6	59.18	40.66	29.72	36.8
	0.06	1556.53	0.3768	6384.3	23.2	103.7	36.096	187.3
	0.06	664.85	0.3768	5704.5	16.18	132.8	29.202	354.9
	0.06	395.35	0.3768	6384.4	30.44	79.03	35.163	124.7
	0.06	452.41	0.3768	6384.4	26.61	90.4	27.439	229.5
	0.09	1384.16	0.5652	6384	34.78	103.7	40.165	158.3
	0.09	1437.7	0.5652	6384.2	33.49	107.7	46.585	131.3

	0.09	1160.24	0.5652	6384.8	41.5	86.96	50.9	70.84
	0.09	1142.3	0.5652	6384.9	42.15	85.62	60.098	42.46
	0.09	1723.99	0.5652	6384.5	27.93	129.2	86.608	49.18
	0.09	1284.71	0.5652	6385	37.48	96.29	67.904	41.8
	0.09	475.96	0.5652	6383.8	25.29	142.7	58.584	143.5
	0.09	322.3	0.5652	6385	37.35	96.62	70.664	36.73
	0.09	291.36	0.5652	6384.9	41.31	87.36	39.306	122.2
	0.12	1		#N/A	#N/A	#N/A	#N/A	#N/A
	0.12	177.92	0.7536	6383.7	67.64	71.12	50.104	41.95
	0.12	366.97	0.7536	6350.9	32.63	146.7	92.381	58.77
	0.12	219.32	0.7536	6384.6	54.88	87.67	75.136	16.68
	0.12	237.68	0.7536	6384.6	50.64	95.01	87.219	8.935
	0.12	422.58	0.7536	6384.3	28.48	168.9	97.45	73.35

48h(right)

Y/m	X/m	resistivity	K	U	I	K*U/I	R	(H-R)/R*100
	0.03	470.42	0.1884	6383	51.16	23.51	16.172	45.35
	0.03	392.46	0.1884	6383.6	61.33	19.61	12.9	52.02
	0.03	903.04	0.1884	5999.8	25.05	45.12	16.844	167.9
	0.03	599.96	0.1884	6383.3	40.12	29.98	22.817	31.37
	0.03	950.98	0.1884	6383	25.31	47.51	26.392	80.03
	0.03	868.48	0.1884	6383.1	27.71	43.4	19.137	126.8
	0.03	717.04	0.1884	6383.5	33.57	35.83	19.031	88.25
	0.03	790.11	0.1884	6383	30.46	39.48	14.455	173.1
	0.03	1326.56	0.1884	6286.7	17.87	66.28	15.147	337.6
	0.03	720.09	0.1884	6383.6	33.43	35.98	19.096	88.39
	0.03	504.35	0.1884	6383.9	47.72	25.2	14.346	75.69
	0.03	957.43	0.1884	6383.4	25.14	47.84	15.593	206.8
	0.03	492.56	0.1884	6383.3	24.43	49.23	12.33	299.2
	0.03	366.32	0.1884	6383.6	32.85	36.61	12.731	187.6
	0.03	287.29	0.1884	6384	41.89	28.71	12.933	122
	0.06	949.75	0.3768	6383	38.01	63.28	34.678	82.46
	0.06	869.62	0.3768	6383.2	41.51	57.94	28.602	102.6
	0.06	1224.27	0.3768	6382.8	29.49	81.55	32.8	148.6
	0.06	1007.46	0.3768	6383.4	35.83	67.13	37.405	79.47
	0.06	1917.45	0.3768	6307.8	18.61	127.7	55.899	128.5
	0.06	984.36	0.3768	6383.5	36.68	65.58	39.223	67.19
	0.06	935.89	0.3768	6383.9	38.58	62.35	40.933	52.32
	0.06	613.09	0.3768	6384.7	58.9	40.84	29.72	37.43
	0.06	1879.88	0.3768	6210.7	18.69	125.2	36.096	246.9
	0.06	822.13	0.3768	6011.1	13.79	164.2	29.202	462.5
	0.06	401.55	0.3768	6383.4	29.97	80.26	35.163	128.2
	0.06	497.17	0.3768	6383.2	24.21	99.35	27.439	262.1
	0.09	1400.07	0.5652	6382.9	34.38	104.9	40.165	161.3
	0.09	1592.28	0.5652	6382.9	30.23	119.3	46.585	156.2
	0.09	1204.95	0.5652	6383.7	39.95	90.31	50.9	77.43
	0.09	1152.7	0.5652	6384	41.76	86.4	60.098	43.77
	0.09	1380.5	0.5652	6383.9	34.87	103.5	86.608	19.47
	0.09	1269.71	0.5652	6383.9	37.91	95.18	67.904	40.16
	0.09	440.95	0.5652	5474.9	23.41	132.2	58.584	125.6

	0.09	315.49	0.5652	6383.8	38.15	94.58	70.664	33.84
	0.09	288.63	0.5652	6384	41.7	86.53	39.306	120.1
	0.12	1		#N/A	#N/A	#N/A	#N/A	#N/A
	0.12	180.89	0.7536	6382.8	66.52	72.31	50.104	44.32
	0.12	293.2	0.7536	6382.7	41.04	117.2	92.381	26.87
	0.12	199.91	0.7536	6383.6	60.2	79.91	75.136	6.355
	0.12	219.42	0.7536	6383.6	54.84	87.72	87.219	0.576
	0.12	334.96	0.7536	6383.5	35.93	133.9	97.45	37.39

FIG6-1 1h(left)

Y/m	X/m	resistivity	K	U	I	K*U/I	R	(H-R)/R*100
	0.03	344.13	0.1884	6385.3	69.96	17.2	16.046	7.166
	0.03	455.43	0.1884	6385.4	52.86	22.76	20.039	13.57
	0.03	700.79	0.1884	5964.8	32.09	35.02	28.302	23.74
	0.03	349.87	0.1884	6385.6	68.81	17.48	17.607	-0.7
	0.03	335.28	0.1884	6385.9	71.81	16.75	16.974	-1.3
	0.03	325.64	0.1884	6385.9	73.94	16.27	16.566	-1.78
	0.03	362.39	0.1884	6385.9	66.44	18.11	18.296	-1.03
	0.03	344.1	0.1884	6386.1	69.97	17.2	17.332	-0.79
	0.03	334.74	0.1884	6386.2	71.93	16.73	17.142	-2.42
	0.03	350.94	0.1884	6386.1	68.61	17.54	18.151	-3.39
	0.03	306.78	0.1884	6386.4	78.49	15.33	15.501	-1.11
	0.03	321.51	0.1884	6386.4	74.89	16.07	16.273	-1.27
	0.03	161.54	0.1884	6386.1	74.52	16.15	16.423	-1.69
	0.03	162.98	0.1884	6386.4	73.87	16.29	17.014	-4.27
	0.03	136.2	0.1884	6386.5	88.39	13.61	13.807	-1.41
	0.06	512.76	0.3768	6385.7	70.43	34.16	31.931	6.992
	0.06	688.23	0.3768	6385.4	52.47	45.86	40.475	13.29
	0.06	1148.56	0.3768	5960.3	29.35	76.52	60.978	25.49
	0.06	576.81	0.3768	6385.8	62.61	38.43	39.064	-1.62
	0.06	535.57	0.3768	6385.9	67.43	35.68	36.383	-1.92
	0.06	538.09	0.3768	6386.1	67.12	35.85	36.818	-2.63
	0.06	600.58	0.3768	6386.1	60.13	40.02	41.256	-3
	0.06	532.71	0.3768	6386.2	67.8	35.49	35.705	-0.6
	0.06	497.79	0.3768	6386.5	72.56	33.16	33.804	-1.89
	0.06	147.09	0.3768	6386.3	81.85	29.4	29.834	-1.46
	0.06	154.04	0.3768	6386.2	78.15	30.79	30.955	-0.53
	0.06	145.39	0.3768	6386.5	82.81	29.06	29.477	-1.42
	0.09	757.55	0.5652	6385.7	63.56	56.78	53.929	5.294
	0.09	963.76	0.5652	6385.5	49.96	72.24	63.94	12.98
	0.09	1714.17	0.5652	6384.8	28.09	128.5	95.717	34.22
	0.09	844.37	0.5652	6385.9	57.03	63.29	65.31	-3.1
	0.09	738.75	0.5652	6386.4	65.19	55.37	56.368	-1.77
	0.09	718.08	0.5652	6386.5	67.06	53.83	54.836	-1.84
	0.09	137.9	0.5652	6386.4	87.31	41.34	42.232	-2.11
	0.09	160.3	0.5652	6386.1	75.1	48.06	47.961	0.209
	0.09	157.81	0.5652	6386.3	76.29	47.31	47.266	0.101
		1						
	0.12	152.57	0.7536	6385.6	78.9	60.99	56.345	8.245
	0.12	204.82	0.7536	6385.2	58.77	81.88	71.899	13.88

	0.12	336.96	0.7536	6233.8	34.88	134.7	105.58	27.56
	0.12	173.4	0.7536	6385.9	69.43	69.31	69.829	-0.74
	0.12	163.39	0.7536	6386.1	73.68	65.32	65.702	-0.59

2h(left)

Y/m	X/m	resistivity	K	U	I	K*U/I	R	(H-R)/R*100
	0.03	412.06	0.1884	6385.3	58.42	20.59	16.046	28.33
	0.03	584.45	0.1884	6385.2	41.19	29.21	20.039	45.74
	0.03	875.48	0.1884	4485.3	19.32	43.74	28.302	54.54
	0.03	412.63	0.1884	6385.6	58.35	20.62	17.607	17.1
	0.03	391.87	0.1884	6385.8	61.44	19.58	16.974	15.36
	0.03	381.02	0.1884	6385.9	63.19	19.04	16.566	14.93
	0.03	426.75	0.1884	6385.9	56.42	21.32	18.296	16.55
	0.03	405.5	0.1884	6386	59.38	20.26	17.332	16.9
	0.03	395.2	0.1884	6386.2	60.93	19.75	17.142	15.19
	0.03	409.01	0.1884	6386.3	58.87	20.44	18.151	12.6
	0.03	359.9	0.1884	6386.3	66.9	17.98	15.501	16.02
	0.03	376.86	0.1884	6386.3	63.89	18.83	16.273	15.73
	0.03	192.03	0.1884	6386.1	62.69	19.19	16.423	16.86
	0.03	189.61	0.1884	6386.4	63.5	18.95	17.014	11.37
	0.03	160.19	0.1884	6386.3	75.16	16.01	13.807	15.94
	0.06	616.76	0.3768	6385.8	58.55	41.1	31.931	28.7
	0.06	883.83	0.3768	6385.2	40.86	58.88	40.475	45.48
	0.06	1440.94	0.3768	4420.3	17.35	96	60.978	57.43
	0.06	672.77	0.3768	6385.8	53.68	44.82	39.064	14.75
	0.06	624.73	0.3768	6385.9	57.81	41.62	36.383	14.4
	0.06	632.82	0.3768	6385.9	57.07	42.16	36.818	14.52
	0.06	698.58	0.3768	6386	51.7	46.54	41.256	12.81
	0.06	625.14	0.3768	6386.2	57.77	41.65	35.705	16.66
	0.06	580.91	0.3768	6386.4	62.17	38.71	33.804	14.5
	0.06	172.87	0.3768	6386	69.64	34.55	29.834	15.82
	0.06	182.31	0.3768	6386.3	66.04	36.44	30.955	17.71
	0.06	171.85	0.3768	6386.4	70.06	34.35	29.477	16.52
	0.09	899.11	0.5652	6385.5	53.55	67.4	53.929	24.97
	0.09	1243.66	0.5652	6385.2	38.72	93.2	63.94	45.77
	0.09	2199.09	0.5652	4706.2	16.14	164.8	95.717	72.18
	0.09	975.98	0.5652	6385.8	49.34	73.15	65.31	12
	0.09	859.21	0.5652	6386.2	56.05	64.4	56.368	14.24
	0.09	836.15	0.5652	6386.3	57.59	62.68	54.836	14.3
	0.09	161.42	0.5652	6386.2	74.58	48.4	42.232	14.6
	0.09	191.5	0.5652	6386.1	62.86	57.42	47.961	19.72
	0.09	187.02	0.5652	6386.2	64.37	56.07	47.266	18.64
		1						
	0.12	183.57	0.7536	6385.4	65.57	73.39	56.345	30.25
	0.12	264.49	0.7536	6385.1	45.51	105.7	71.899	47.06
	0.12	414.65	0.7536	4633.9	21.07	165.7	105.58	56.98
	0.12	204.45	0.7536	6385.9	58.88	81.73	69.829	17.05
	0.12	191.91	0.7536	6385.9	62.73	76.72	65.702	16.77

3h(left)

Y/m	X/m	resistivity	K	U	I	K*U/I	R	(H-R)/R*100
	0.03	417.28	0.1884	6384.7	57.69	20.85	16.046	29.95
	0.03	595.59	0.1884	6384.7	40.42	29.76	20.039	48.51
	0.03	789.75	0.1884	5008.8	23.91	39.47	28.302	39.45
	0.03	410.53	0.1884	6385.1	58.64	20.51	17.607	16.51
	0.03	389.63	0.1884	6385.4	61.79	19.47	16.974	14.7
	0.03	379.59	0.1884	6385.5	63.42	18.97	16.566	14.51
	0.03	424.85	0.1884	6385.3	56.66	21.23	18.296	16.04
	0.03	403.36	0.1884	6385.5	59.69	20.15	17.332	16.28
	0.03	395.41	0.1884	6385.7	60.89	19.76	17.142	15.26
	0.03	407.91	0.1884	6385.8	59.02	20.38	18.151	12.3
	0.03	357.69	0.1884	6385.7	67.31	17.87	15.501	15.3
	0.03	374.71	0.1884	6385.8	64.25	18.72	16.273	15.07
	0.03	192.73	0.1884	6385.5	62.46	19.26	16.423	17.28
	0.03	189.48	0.1884	6385.8	63.53	18.94	17.014	11.31
	0.03	159.29	0.1884	6385.8	75.57	15.92	13.807	15.31
	0.06	624.58	0.3768	6385.2	57.82	41.61	31.931	30.31
	0.06	902.94	0.3768	6384.6	39.99	60.16	40.475	48.63
	0.06	1286.15	0.3768	4983.8	21.92	85.67	60.978	40.49
	0.06	668.38	0.3768	6385.1	54.03	44.53	39.064	13.99
	0.06	620.95	0.3768	6385.3	58.15	41.38	36.383	13.72
	0.06	631.14	0.3768	6385.4	57.22	42.05	36.818	14.21
	0.06	696.02	0.3768	6385.4	51.88	46.38	41.256	12.41
	0.06	621.02	0.3768	6385.5	58.15	41.38	35.705	15.89
	0.06	577.28	0.3768	6385.9	62.56	38.46	33.804	13.78
	0.06	172.09	0.3768	6385.6	69.95	34.4	29.834	15.3
	0.06	181.62	0.3768	6385.7	66.28	36.3	30.955	17.27
	0.06	171.05	0.3768	6385.9	70.38	34.19	29.477	15.98
	0.09	907.52	0.5652	6385	53.05	68.03	53.929	26.14
	0.09	1268.56	0.5652	6384.6	37.95	95.09	63.94	48.71
	0.09	2032.25	0.5652	5603.8	20.8	152.3	95.717	59.09
	0.09	970.8	0.5652	6385.1	49.6	72.76	65.31	11.41
	0.09	853.91	0.5652	6385.7	56.39	64	56.368	13.55
	0.09	832.2	0.5652	6385.7	57.86	62.38	54.836	13.75
	0.09	160.61	0.5652	6385.7	74.95	48.15	42.232	14.02
	0.09	191.1	0.5652	6385.6	62.99	57.3	47.961	19.46
	0.09	186.24	0.5652	6385.7	64.64	55.83	47.266	18.13
		1						
	0.12	185.86	0.7536	6384.9	64.76	74.3	56.345	31.87
	0.12	269.61	0.7536	6384.4	44.64	107.8	71.899	49.91
	0.12	382.83	0.7536	5227.5	25.74	153	105.58	44.95
	0.12	203.63	0.7536	6385.2	59.11	81.41	69.829	16.58
	0.12	191.07	0.7536	6385.4	63	76.38	65.702	16.25

5h(left)

Y/m	X/m	resistivity	K	U	I	K*U/I	R	(H-R)/R*100
	0.03	415.12	0.1884	6383.7	57.98	20.74	16.046	29.28
	0.03	639.86	0.1884	6383.8	37.62	31.97	20.039	59.54

	0.03	1009.44	0.1884	4061.2	15.17	50.44	28.302	78.21
	0.03	391.96	0.1884	6384.4	61.41	19.59	17.607	11.24
	0.03	370.22	0.1884	6384.6	65.02	18.5	16.974	8.988
	0.03	361.18	0.1884	6384.8	66.65	18.05	16.566	8.946
	0.03	406.09	0.1884	6384.6	59.28	20.29	18.296	10.9
	0.03	384.63	0.1884	6384.8	62.58	19.22	17.332	10.9
	0.03	373.13	0.1884	6385.1	64.52	18.64	17.142	8.764
	0.03	386.18	0.1884	6385.1	62.34	19.3	18.151	6.313
	0.03	340.53	0.1884	6385.1	70.69	17.02	15.501	9.781
	0.03	356.83	0.1884	6385	67.46	17.83	16.273	9.581
	0.03	182.35	0.1884	6384.9	66.01	18.22	16.423	10.96
	0.03	178.49	0.1884	6385.2	67.44	17.84	17.014	4.843
	0.03	151.95	0.1884	6385.2	79.22	15.19	13.807	9.982
	0.06	619.55	0.3768	6384.4	58.28	41.28	31.931	29.27
	0.06	977.2	0.3768	6383.9	36.95	65.1	40.475	60.84
	0.06	1642.4	0.3768	3963.9	13.65	109.4	60.978	79.44
	0.06	633.25	0.3768	6384.4	57.02	42.19	39.064	8.002
	0.06	589.63	0.3768	6384.7	61.24	39.28	36.383	7.973
	0.06	594.86	0.3768	6384.8	60.7	39.63	36.818	7.65
	0.06	661.13	0.3768	6384.9	54.62	44.05	41.256	6.765
	0.06	591.26	0.3768	6385	61.07	39.4	35.705	10.34
	0.06	547.93	0.3768	6385.3	65.9	36.51	33.804	8.003
	0.06	164.49	0.3768	6385	73.17	32.88	29.834	10.21
	0.06	173.3	0.3768	6385	69.46	34.64	30.955	11.89
	0.06	163.08	0.3768	6385.3	73.81	32.6	29.477	10.58
	0.09	893.56	0.5652	6384.2	53.88	66.97	53.929	24.18
	0.09	1387.3	0.5652	6383.7	34.7	104	63.94	62.62
	0.09	2416.38	0.5652	4029.3	12.58	181	95.717	89.13
	0.09	916.08	0.5652	6384.5	52.55	68.67	65.31	5.142
	0.09	810.63	0.5652	6385	59.39	60.76	56.368	7.8
	0.09	787.47	0.5652	6385.2	61.14	59.03	54.836	7.642
	0.09	152.8	0.5652	6385.1	78.78	45.81	42.232	8.469
	0.09	184.41	0.5652	6384.9	65.27	55.29	47.961	15.28
	0.09	177.85	0.5652	6385	67.68	53.32	47.266	12.81
		1						
	0.12	185.46	0.7536	6383.8	64.89	74.14	56.345	31.58
	0.12	289.9	0.7536	6383.5	41.51	115.9	71.899	61.19
	0.12	482.42	0.7536	4245	16.59	192.8	105.58	82.63
	0.12	195.29	0.7536	6384.6	61.63	78.07	69.829	11.8
	0.12	182.29	0.7536	6384.8	66.03	72.87	65.702	10.91

10h(left)

Y/m	X/m	resistivity	K	U	I	K*U/I	R	(H-R)/R*100
	0.03	512.79	0.1884	6383.6	46.94	25.62	16.046	59.68
	0.03	805.79	0.1884	6383.4	29.87	40.26	20.039	100.9
	0.03	876.46	0.1884	4746.2	20.42	43.79	28.302	54.72
	0.03	402.5	0.1884	6384.1	59.8	20.11	17.607	14.23
	0.03	384.07	0.1884	6384.5	62.67	19.19	16.974	13.07
	0.03	374.89	0.1884	6384.6	64.21	18.73	16.566	13.08
	0.03	419.11	0.1884	6384.4	57.43	20.94	18.296	14.47

	0.03	396.43	0.1884	6384.6	60.72	19.81	17.332	14.3
	0.03	381.49	0.1884	6384.8	63.1	19.06	17.142	11.21
	0.03	394.53	0.1884	6384.7	61.02	19.71	18.151	8.605
	0.03	349.87	0.1884	6384.9	68.8	17.48	15.501	12.79
	0.03	367.75	0.1884	6385	65.46	18.38	16.273	12.93
	0.03	185.19	0.1884	6384.7	64.99	18.51	16.423	12.7
	0.03	181.1	0.1884	6384.9	66.46	18.1	17.014	6.384
	0.03	154.2	0.1884	6384.9	78.06	15.41	13.807	11.61
	0.06	769.3	0.3768	6384	46.93	51.26	31.931	60.52
	0.06	1243.91	0.3768	6383.3	29.02	82.88	40.475	104.8
	0.06	1435.36	0.3768	4770.9	18.8	95.62	60.978	56.81
	0.06	650.17	0.3768	6384.3	55.53	43.32	39.064	10.9
	0.06	609.96	0.3768	6384.3	59.19	40.64	36.383	11.71
	0.06	612.47	0.3768	6384.6	58.95	40.81	36.818	10.84
	0.06	683.47	0.3768	6384.6	52.83	45.54	41.256	10.38
	0.06	612.66	0.3768	6384.8	58.94	40.82	35.705	14.32
	0.06	567.96	0.3768	6385	63.58	37.84	33.804	11.94
	0.06	169.96	0.3768	6384.7	70.82	33.97	29.834	13.86
	0.06	177.07	0.3768	6384.8	67.97	35.39	30.955	14.34
	0.06	166.67	0.3768	6385	72.22	33.31	29.477	13.01
	0.09	1100.79	0.5652	6383.8	43.73	82.51	53.929	53
	0.09	1790.16	0.5652	6383.3	26.89	134.2	63.94	109.8
	0.09	2230.2	0.5652	5319.3	17.99	167.1	95.717	74.6
	0.09	944.58	0.5652	6384.3	50.97	70.79	65.31	8.398
	0.09	842.21	0.5652	6384.8	57.16	63.13	56.368	12
	0.09	818.68	0.5652	6384.8	58.81	61.36	54.836	11.9
	0.09	158.75	0.5652	6384.8	75.82	47.6	42.232	12.7
	0.09	191.08	0.5652	6384.6	62.99	57.29	47.961	19.45
	0.09	184.49	0.5652	6384.8	65.24	55.31	47.266	17.03
		1						
	0.12	230.53	0.7536	6383.8	52.2	92.16	56.345	63.57
	0.12	365.45	0.7536	6303.4	32.52	146.1	71.899	103.2
	0.12	420.51	0.7536	4938.1	22.14	168.1	105.58	59.19
	0.12	200.52	0.7536	6384.5	60.02	80.16	69.829	14.8
	0.12	189.47	0.7536	6384.5	63.52	75.75	65.702	15.29

15h(left)

Y/m	X/m	resistivity	K	U	I	K*U/I	R	(H-R)/R*100
	0.03	509.93	0.1884	6385.4	47.21	25.48	16.046	58.81
	0.03	624.07	0.1884	6384.4	38.57	31.19	20.039	55.62
	0.03	487.21	0.1884	6384.7	49.41	24.34	28.302	-14
	0.03	429.15	0.1884	6385.4	56.1	21.44	17.607	21.79
	0.03	387.07	0.1884	6385.7	62.2	19.34	16.974	13.95
	0.03	373.8	0.1884	6385.9	64.41	18.68	16.566	12.75
	0.03	413.94	0.1884	6385.7	58.16	20.69	18.296	13.06
	0.03	391.88	0.1884	6385.8	61.44	19.58	17.332	12.98
	0.03	382.08	0.1884	6386	63.02	19.09	17.142	11.37
	0.03	388.23	0.1884	6386.1	62.02	19.4	18.151	6.877
	0.03	346.7	0.1884	6386	69.44	17.33	15.501	11.77
	0.03	366.09	0.1884	6386.1	65.77	18.29	16.273	12.42

	0.03	185.26	0.1884	6385.9	64.98	18.52	16.423	12.74
	0.03	178.26	0.1884	6386.2	67.54	17.81	17.014	4.705
	0.03	151.69	0.1884	6386.3	79.36	15.16	13.807	9.806
	0.06	757.88	0.3768	6384.6	47.64	50.5	31.931	58.15
	0.06	932.4	0.3768	6384.5	38.73	62.11	40.475	53.46
	0.06	789.91	0.3768	6384.9	45.71	52.63	60.978	-13.7
	0.06	691.75	0.3768	6385.5	52.2	46.09	39.064	17.99
	0.06	611.5	0.3768	6385.8	59.06	40.74	36.383	11.98
	0.06	613.69	0.3768	6385.9	58.85	40.89	36.818	11.05
	0.06	674.85	0.3768	6385.9	53.52	44.96	41.256	8.977
	0.06	607.28	0.3768	6386	59.47	40.46	35.705	13.32
	0.06	566.02	0.3768	6386.2	63.81	37.71	33.804	11.56
	0.06	169.51	0.3768	6385.9	71.02	33.88	29.834	13.56
	0.06	173.95	0.3768	6386.1	69.21	34.77	30.955	12.32
	0.06	164.51	0.3768	6386.3	73.18	32.88	29.477	11.55
	0.09	1077.33	0.5652	6384.7	44.69	80.75	53.929	49.73
	0.09	1294.51	0.5652	6384.7	37.19	97.03	63.94	51.75
	0.09	1128.56	0.5652	6385	42.66	84.59	95.717	-11.6
	0.09	999.51	0.5652	6385.6	48.18	74.91	65.31	14.7
	0.09	846.46	0.5652	6386.1	56.89	63.45	56.368	12.56
	0.09	815.5	0.5652	6386.2	59.05	61.13	54.836	11.47
	0.09	158.77	0.5652	6386	75.82	47.6	42.232	12.72
	0.09	187.72	0.5652	6386	64.13	56.28	47.961	17.35
	0.09	183.23	0.5652	6386.1	65.7	54.94	47.266	16.23
		1						
	0.12	257.86	0.7536	6384.4	46.68	103.1	56.345	82.92
	0.12	298.93	0.7536	6385.9	40.27	119.5	71.899	66.21
	0.12	234.7	0.7536	6384.7	51.28	93.83	105.58	-11.1
	0.12	213.53	0.7536	6385.5	56.38	85.35	69.829	22.23
	0.12	190.61	0.7536	6385.8	63.16	76.19	65.702	15.97

36h(left)

Y/m	X/m	resistivity	K	U	I	K*U/I	R	(H-R)/R*100
	0.03	551.68	0.1884	6384.1	43.63	27.57	16.046	71.81
	0.03	627.9	0.1884	6383.1	38.33	31.37	20.039	56.57
	0.03	446.95	0.1884	6383.7	53.85	22.33	28.302	-21.1
	0.03	409.81	0.1884	6384.1	58.73	20.48	17.607	16.31
	0.03	360.58	0.1884	6384.3	66.75	18.02	16.974	6.157
	0.03	380.87	0.1884	6384.5	63.2	19.03	16.566	14.89
	0.03	359.05	0.1884	6384.5	67.04	17.94	18.296	-1.94
	0.03	323.2	0.1884	6384.6	74.48	16.15	17.332	-6.82
	0.03	302.67	0.1884	6384.9	79.53	15.13	17.142	-11.8
	0.03	318.42	0.1884	6384.9	75.6	15.91	18.151	-12.3
	0.03	281.21	0.1884	6385	85.6	14.05	15.501	-9.34
	0.03	298.22	0.1884	6384.9	80.72	14.9	16.273	-8.42
	0.03	143.81	0.1884	6384.7	83.69	14.37	16.423	-12.5
	0.03	143.76	0.1884	6385.2	83.73	14.37	17.014	-15.6
	0.03	120.11	0.1884	6385.1	100.2	12	13.807	-13.1
	0.06	791.62	0.3768	6383.5	45.61	52.74	31.931	65.16
	0.06	932.52	0.3768	6383.3	38.71	62.13	40.475	53.52

	0.06	713.52	0.3768	6383.5	50.6	47.54	60.978	-22
	0.06	649	0.3768	6384	55.63	43.24	39.064	10.69
	0.06	560.69	0.3768	6384.3	64.39	37.36	36.383	2.685
	0.06	601.03	0.3768	6384.3	60.07	40.05	36.818	8.77
	0.06	593.54	0.3768	6384.7	60.83	39.55	41.256	-4.14
	0.06	502.42	0.3768	6384.8	71.87	33.47	35.705	-6.25
	0.06	468.39	0.3768	6385.2	77.09	31.21	33.804	-7.68
	0.06	138.58	0.3768	6384.9	86.85	27.7	29.834	-7.15
	0.06	139.4	0.3768	6385	86.34	27.86	30.955	-9.98
	0.06	133	0.3768	6385.2	90.5	26.59	29.477	-9.81
	0.09	1115.57	0.5652	6383.5	43.15	83.61	53.929	55.04
	0.09	1291.72	0.5652	6383.3	37.26	96.83	63.94	51.44
	0.09	998.32	0.5652	6383.7	48.22	74.82	95.717	-21.8
	0.09	944.14	0.5652	6384.3	50.99	70.77	65.31	8.355
	0.09	778	0.5652	6384.7	61.88	58.32	56.368	3.456
	0.09	826.52	0.5652	6384.7	58.25	61.95	54.836	12.97
	0.09	170.44	0.5652	6384.5	70.62	51.1	42.232	20.99
	0.09	165.03	0.5652	6384.6	72.93	49.48	47.961	3.166
	0.09	153.42<br>1	0.5652	6384.9	78.45	46	47.266	-2.68
	0.12	284.22	0.7536	6382.9	42.34	113.6	56.345	101.6
	0.12	316.04	0.7536	6384.6	38.09	126.3	71.899	75.69
	0.12	248.15	0.7536	6383.4	48.49	99.21	105.58	-6.04
	0.12	214.6	0.7536	6384.4	56.08	85.79	69.829	22.86
	0.12	179.4	0.7536	6384.9	67.09	71.72	65.702	9.159

48h(left)

Y/m	X/m	resistivity	K	U	I	K*U/I	R	(H-R)/R*100
	0.03	593.02	0.1884	6384.1	40.59	29.63	16.046	84.67
	0.03	620.66	0.1884	6383.5	38.78	31.01	20.039	54.76
	0.03	445.71	0.1884	6383.9	54	22.27	28.302	-21.3
	0.03	395.17	0.1884	6384.7	60.92	19.75	17.607	12.14
	0.03	347.75	0.1884	6384.5	69.22	17.38	16.974	2.374
	0.03	371.43	0.1884	6384.5	64.81	18.56	16.566	12.03
	0.03	351.62	0.1884	6384.5	68.46	17.57	18.296	-3.97
	0.03	304.98	0.1884	6384.8	78.93	15.24	17.332	-12.1
	0.03	280.38	0.1884	6385	85.86	14.01	17.142	-18.3
	0.03	300.85	0.1884	6385	80.02	15.03	18.151	-17.2
	0.03	266.23	0.1884	6385.2	90.42	13.3	15.501	-14.2
	0.03	282.93	0.1884	6385.1	85.08	14.14	16.273	-13.1
	0.03	130.6	0.1884	6385	92.16	13.05	16.423	-20.5
	0.03	135.38	0.1884	6385.2	88.91	13.53	17.014	-20.5
	0.03	113.22	0.1884	6385.1	106.3	11.32	13.807	-18
	0.06	841.19	0.3768	6383.6	42.92	56.04	31.931	75.51
	0.06	922.87	0.3768	6383.6	39.12	61.49	40.475	51.91
	0.06	703.99	0.3768	6383.8	51.28	46.91	60.978	-23.1
	0.06	625.03	0.3768	6384.2	57.77	41.64	39.064	6.596
	0.06	540.54	0.3768	6384.6	66.8	36.01	36.383	-1.02
	0.06	579.25	0.3768	6384.6	62.33	38.6	36.818	4.831
	0.06	581.69	0.3768	6384.7	62.07	38.76	41.256	-6.05

	0.06	477.71	0.3768	6385.1	75.59	31.83	35.705	-10.9
	0.06	449.51	0.3768	6385.3	80.33	29.95	33.804	-11.4
	0.06	130.62	0.3768	6385	92.15	26.11	29.834	-12.5
	0.06	130.49	0.3768	6385.2	92.24	26.08	30.955	-15.7
	0.06	124.87	0.3768	6385.5	96.4	24.96	29.477	-15.3
	0.09	1181.22	0.5652	6383.6	40.75	88.54	53.929	64.18
	0.09	1275.31	0.5652	6383.6	37.75	95.58	63.94	49.48
	0.09	973.79	0.5652	6384	49.43	73	95.717	-23.7
	0.09	909.04	0.5652	6384.3	52.96	68.13	65.31	4.325
	0.09	751.97	0.5652	6384.7	64.02	56.37	56.368	-0
	0.09	811.08	0.5652	6384.7	59.36	60.79	54.836	10.86
	0.09	167.93	0.5652	6384.9	71.68	50.35	42.232	19.21
	0.09	161.9	0.5652	6384.8	74.34	48.54	47.961	1.213
	0.09	144.44	0.5652	6385	83.33	43.31	47.266	-8.37
		1						
	0.12	300.49	0.7536	6382.9	40.05	120.1	56.345	113.2
	0.12	310.65	0.7536	6384.8	38.75	124.2	71.899	72.7
	0.12	249.1	0.7536	6383.5	48.31	99.58	105.58	-5.69
	0.12	211.14	0.7536	6384.5	57	84.41	69.829	20.88
	0.12	172.97	0.7536	6385.1	69.59	69.14	65.702	5.24

1h(right)

Y/m	X/m	resistiv	K	U	I	K*U/I	R	((K*U/I)-R)/R*100
	0.03	389.32	0.1884	6385.3	61.84	19.45	18.004	8.048
	0.03	501.28	0.1884	6384.8	48.02	25.05	23.918	4.735
	0.03	405.98	0.1884	6385.5	59.3	20.29	20.005	1.409
	0.03	382.2	0.1884	6385.7	62.99	19.1	18.873	1.202
	0.03	404.55	0.1884	6385.5	59.51	20.22	19.866	1.758
	0.03	420.57	0.1884	6385.7	57.25	21.01	20.493	2.544
	0.03	492.81	0.1884	6385.6	48.85	24.63	23.49	4.844
	0.03	340.14	0.1884	6386.1	70.79	17	16.946	0.292
	0.03	351.86	0.1884	6385.9	68.42	17.58	17.629	-0.25
	0.03	372.67	0.1884	6386.1	64.61	18.62	18.993	-1.96
	0.03	383.74	0.1884	6386.1	62.74	19.18	19.26	-0.44
	0.03	350.9	0.1884	6386.2	68.62	17.53	17.588	-0.31
	0.03	157.13	0.1884	6386.5	76.62	15.7	15.698	0.038
	0.03	210.44	0.1884	6385.8	57.21	21.03	20.773	1.235
	0.03	162.91	0.1884	6386.4	73.9	16.28	16.476	-1.18
	0.06	568.66	0.3768	6385.5	63.5	37.89	35.817	5.79
	0.06	792.33	0.3768	6385.1	45.58	52.78	50.316	4.905
	0.06	633.92	0.3768	6385.4	56.97	42.23	41.652	1.396
	0.06	729.04	0.3768	6385.3	49.53	48.58	47.319	2.656
	0.06	604.06	0.3768	6385.8	59.79	40.24	39.827	1.047
	0.06	638.51	0.3768	6385.9	56.56	42.54	42.083	1.091
	0.06	660.01	0.3768	6385.8	54.72	43.97	43.664	0.706
	0.06	611.67	0.3768	6385.8	59.04	40.76	41.113	-0.87
	0.06	576.16	0.3768	6386.2	62.68	38.39	38.528	-0.36

	0.06	170.63	0.3768	6386.4	70.56	34.1	33.452	1.951
	0.06	209.57	0.3768	6385.6	57.44	41.89	40.531	3.351
	0.06	153.79	0.3768	6386.4	78.29	30.74	30.489	0.813
	0.09	955.57	0.5652	6385.1	50.39	71.62	67.476	6.14
	0.09	1066.29	0.5652	6385.1	45.15	79.93	75.979	5.202
	0.09	855.78	0.5652	6385.7	56.27	64.14	63.84	0.471
	0.09	871.33	0.5652	6385.7	55.26	65.31	66.144	-1.26
	0.09	930.87	0.5652	6385.9	51.73	69.77	69.844	-0.1
	0.09	918.09	0.5652	6385.9	52.45	68.81	68.145	0.982
	0.09	198.86	0.5652	6385.9	60.54	59.62	58.003	2.786
	0.09	187.54	0.5652	6385.9	64.19	56.23	54.228	3.69
	0.09	168.43	0.5652	6386.1	71.48	50.5	49.774	1.451
		1						
	0.12	166.16	0.7536	6385.3	72.44	66.43	61.901	7.311
	0.12	252.34	0.7536	6384.9	47.7	100.9	95.918	5.167
	0.12	210.44	0.7536	6385.4	57.2	84.13	81.941	2.668
	0.12	195.82	0.7536	6385.7	61.48	78.27	75.799	3.265
	0.12	185.51	0.7536	6386	64.89	74.16	72.797	1.877

2h(right)

Y/m	X/m	resistiv	K	U	I	K*U/I	R	((K*U/I)-R)/R*100
	0.03	484.98	0.1884	6385.1	49.64	24.23	18.004	34.6
	0.03	617.45	0.1884	6384.9	38.99	30.85	23.918	28.99
	0.03	475.68	0.1884	6385.4	50.61	23.77	20.005	18.82
	0.03	457.87	0.1884	6385.6	52.58	22.88	18.873	21.24
	0.03	487.21	0.1884	6385.5	49.42	24.34	19.866	22.53
	0.03	498.31	0.1884	6385.7	48.32	24.9	20.493	21.5
	0.03	603.35	0.1884	6385.5	39.9	30.15	23.49	28.36
	0.03	400.46	0.1884	6386.1	60.12	20.01	16.946	18.09
	0.03	419.45	0.1884	6386	57.4	20.96	17.629	18.9
	0.03	426.14	0.1884	6386.2	56.5	21.29	18.993	12.12
	0.03	449.63	0.1884	6386	53.55	22.47	19.26	16.65
	0.03	419.61	0.1884	6386.1	57.38	20.97	17.588	19.22
	0.03	192.44	0.1884	6386.3	62.56	19.23	15.698	22.52
	0.03	247.5	0.1884	6385.8	48.64	24.73	20.773	19.07
	0.03	190.55	0.1884	6386.4	63.18	19.04	16.476	15.59
	0.06	691.12	0.3768	6385.3	52.25	46.05	35.817	28.56
	0.06	975.72	0.3768	6384.9	37.01	65.01	50.316	29.19
	0.06	751.85	0.3768	6385.4	48.03	50.09	41.652	20.27
	0.06	877.35	0.3768	6385.3	41.16	58.45	47.319	23.53
	0.06	723.44	0.3768	6385.9	49.92	48.2	39.827	21.03
	0.06	746.73	0.3768	6385.8	48.36	49.76	42.083	18.23
	0.06	775.64	0.3768	6385.8	46.56	51.68	43.664	18.36
	0.06	712.49	0.3768	6386	50.69	47.47	41.113	15.46
	0.06	674.53	0.3768	6386.1	53.54	44.94	38.528	16.65
	0.06	207.15	0.3768	6386.4	58.12	41.4	33.452	23.77
	0.06	253.9	0.3768	6385.6	47.41	50.75	40.531	25.22
	0.06	182.37	0.3768	6386.5	66.02	36.45	30.489	19.55

	0.09	1167.45	0.5652	6385.1	41.24	87.51	67.476	29.69
	0.09	1325.04	0.5652	6385	36.34	99.31	75.979	30.7
	0.09	1007.33	0.5652	6385.8	47.8	75.51	63.84	18.28
	0.09	1006.95	0.5652	6385.7	47.82	75.48	66.144	14.11
	0.09	1101.91	0.5652	6385.7	43.7	82.59	69.844	18.25
	0.09	1058.09	0.5652	6385.9	45.51	79.31	68.145	16.38
	0.09	232.86	0.5652	6385.9	51.7	69.81	58.003	20.36
	0.09	230.03	0.5652	6385.7	52.33	68.97	54.228	27.19
	0.09	200.36	0.5652	6386.1	60.09	60.07	49.774	20.68
		1						
	0.12	205.5	0.7536	6385.3	58.57	82.16	61.901	32.72
	0.12	314	0.7536	6385	38.33	125.5	95.918	30.88
	0.12	244.29	0.7536	6385.5	49.28	97.65	81.941	19.17
	0.12	240.41	0.7536	6385.5	50.07	96.11	75.799	26.79
	0.12	224.39	0.7536	6385.8	53.65	89.7	72.797	23.22

3h(right)

Y/m	X/m	resistiv	K	U	I	K*U/I	R	((K*U/I)-R)/R*100
	0.03	479.67	0.1884	6384.8	50.19	23.97	18.004	33.12
	0.03	608.47	0.1884	6384.5	39.56	30.41	23.918	27.13
	0.03	471.78	0.1884	6385.1	51.03	23.57	20.005	17.84
	0.03	448.05	0.1884	6385.4	53.73	22.39	18.873	18.64
	0.03	475.4	0.1884	6385.2	50.64	23.76	19.866	19.58
	0.03	491.22	0.1884	6385.3	49.01	24.55	20.493	19.78
	0.03	587.93	0.1884	6385.1	40.95	29.38	23.49	25.06
	0.03	393.91	0.1884	6385.8	61.12	19.68	16.946	16.15
	0.03	410.81	0.1884	6385.8	58.61	20.53	17.629	16.44
	0.03	415.83	0.1884	6385.8	57.9	20.78	18.993	9.401
	0.03	441.12	0.1884	6385.9	54.58	22.04	19.26	14.45
	0.03	411.2	0.1884	6385.8	58.55	20.55	17.588	16.83
	0.03	186.72	0.1884	6386	64.47	18.66	15.698	18.88
	0.03	241.69	0.1884	6385.4	49.81	24.15	20.773	16.27
	0.03	187.07	0.1884	6386.1	64.35	18.7	16.476	13.48
	0.06	690.94	0.3768	6384.9	52.26	46.04	35.817	28.53
	0.06	953.56	0.3768	6384.6	37.87	63.53	50.316	26.25
	0.06	740.26	0.3768	6385.1	48.78	49.32	41.652	18.41
	0.06	855.58	0.3768	6384.8	42.21	57	47.319	20.45
	0.06	705.41	0.3768	6385.4	51.19	47	39.827	18.02
	0.06	737.92	0.3768	6385.4	48.94	49.16	42.083	16.82
	0.06	756.41	0.3768	6385.4	47.74	50.4	43.664	15.42
	0.06	702.18	0.3768	6385.6	51.43	46.78	41.113	13.79
	0.06	666.41	0.3768	6385.7	54.19	44.4	38.528	15.25
	0.06	202.34	0.3768	6386.1	59.5	40.44	33.452	20.9
	0.06	248.87	0.3768	6385.4	48.37	49.74	40.531	22.73
	0.06	178.77	0.3768	6386.1	67.34	35.73	30.489	17.2
	0.09	1155.96	0.5652	6384.7	41.65	86.64	67.476	28.41
	0.09	1283.99	0.5652	6384.8	37.5	96.23	75.979	26.66
	0.09	994.08	0.5652	6385.3	48.44	74.5	63.84	16.71

	0.09	986.49	0.5652	6385.4	48.81	73.94	66.144	11.79
	0.09	1076.89	0.5652	6385.4	44.71	80.72	69.844	15.57
	0.09	1050.95	0.5652	6385.6	45.82	78.77	68.145	15.59
	0.09	231.36	0.5652	6385.5	52.03	69.37	58.003	19.59
	0.09	224.46	0.5652	6385.5	53.63	67.3	54.228	24.1
	0.09	196.92	0.5652	6385.8	61.13	59.04	49.774	18.62
		1						
	0.12	206.29	0.7536	6385	58.35	82.46	61.901	33.22
	0.12	308.13	0.7536	6384.6	39.06	123.2	95.918	28.42
	0.12	243.9	0.7536	6385	49.35	97.5	81.941	18.99
	0.12	234.37	0.7536	6385.2	51.36	93.69	75.799	23.6
	0.12	219.62	0.7536	6385.4	54.81	87.8	72.797	20.6

5h(right)

Y/m	X/m	resistiv	K	U	I	K*U/I	R	((K*U/I)-R)/R*100
	0.03	470.81	0.1884	6384.2	51.13	23.52	18.004	30.66
	0.03	587.34	0.1884	6384	40.98	29.35	23.918	22.71
	0.03	455.47	0.1884	6384.8	52.85	22.76	20.005	13.77
	0.03	429.58	0.1884	6384.8	56.04	21.46	18.873	13.74
	0.03	457.53	0.1884	6384.6	52.61	22.86	19.866	15.09
	0.03	475.82	0.1884	6384.8	50.59	23.78	20.493	16.03
	0.03	572.98	0.1884	6384.6	42.01	28.63	23.49	21.89
	0.03	378.26	0.1884	6385.3	63.64	18.9	16.946	11.55
	0.03	394.86	0.1884	6385.2	60.97	19.73	17.629	11.92
	0.03	384.86	0.1884	6385.4	62.55	19.23	18.993	1.261
	0.03	422.11	0.1884	6385.2	57.03	21.09	19.26	9.518
	0.03	394.12	0.1884	6385.3	61.08	19.7	17.588	11.98
	0.03	179.7	0.1884	6385.5	66.99	17.96	15.698	14.4
	0.03	227.76	0.1884	6385	52.85	22.76	20.773	9.574
	0.03	178.46	0.1884	6385.6	67.45	17.84	16.476	8.258
	0.06	673.48	0.3768	6384.3	53.61	44.87	35.817	25.28
	0.06	919.93	0.3768	6384.2	39.25	61.29	50.316	21.81
	0.06	712.42	0.3768	6384.7	50.68	47.47	41.652	13.97
	0.06	828.03	0.3768	6384.5	43.61	55.16	47.319	16.58
	0.06	676.31	0.3768	6385	53.39	45.06	39.827	13.14
	0.06	712.02	0.3768	6384.9	50.71	47.44	42.083	12.74
	0.06	709.38	0.3768	6385.1	50.9	47.27	43.664	8.251
	0.06	670.57	0.3768	6385.1	53.85	44.68	41.113	8.669
	0.06	639.04	0.3768	6385.3	56.51	42.58	38.528	10.51
	0.06	195.44	0.3768	6385.5	61.59	39.07	33.452	16.78
	0.06	242.38	0.3768	6384.7	49.66	48.44	40.531	19.53
	0.06	172.32	0.3768	6385.5	69.86	34.44	30.489	12.96
	0.09	1128.55	0.5652	6384.2	42.66	84.58	67.476	25.35
	0.09	1237.46	0.5652	6384.3	38.9	92.76	75.979	22.09
	0.09	954.42	0.5652	6384.8	50.44	71.54	63.84	12.07
	0.09	920.38	0.5652	6385	52.31	68.99	66.144	4.3
	0.09	1027.64	0.5652	6385	46.85	77.03	69.844	10.29
	0.09	1011.98	0.5652	6385.1	47.58	75.85	68.145	11.3

	0.09	225.05	0.5652	6384.9	53.48	67.48	58.003	16.34
	0.09	216.75	0.5652	6384.9	55.53	64.99	54.228	19.84
	0.09	190.23	0.5652	6385.3	63.28	57.03	49.774	14.58
		1						
	0.12	201.94	0.7536	6384.5	59.6	80.73	61.901	30.41
	0.12	297.58	0.7536	6384.1	40.44	119	95.918	24.03
	0.12	236.68	0.7536	6384.6	50.85	94.62	81.941	15.47
	0.12	226.82	0.7536	6384.7	53.07	90.66	75.799	19.61
	0.12	211.79	0.7536	6385	56.83	84.67	72.797	16.31

10h(right)

Y/m	X/m	resistiv	K	U	I	K*U/I	R	((K*U/I)-R)/R*100
	0.03	508.27	0.1884	6383.4	47.35	25.4	18.004	41.07
	0.03	643.76	0.1884	6383.2	37.39	32.16	23.918	34.48
	0.03	515.79	0.1884	6383.6	46.66	25.78	20.005	28.84
	0.03	443.23	0.1884	6384.1	54.31	22.15	18.873	17.35
	0.03	473.08	0.1884	6383.9	50.88	23.64	19.866	18.99
	0.03	492.31	0.1884	6384	48.89	24.6	20.493	20.05
	0.03	593.91	0.1884	6383.8	40.53	29.67	23.49	26.33
	0.03	389.87	0.1884	6384.4	61.74	19.48	16.946	14.96
	0.03	406.25	0.1884	6384.2	59.25	20.3	17.629	15.16
	0.03	392.81	0.1884	6384.6	61.28	19.63	18.993	3.347
	0.03	427.59	0.1884	6384.4	56.29	21.37	19.26	10.94
	0.03	403.48	0.1884	6384.5	59.66	20.16	17.588	14.63
	0.03	183.14	0.1884	6384.8	65.72	18.3	15.698	16.6
	0.03	235.41	0.1884	6384.2	51.12	23.53	20.773	13.27
	0.03	179.19	0.1884	6384.8	67.17	17.91	16.476	8.696
	0.06	726.19	0.3768	6383.5	49.71	48.39	35.817	35.09
	0.06	1004.39	0.3768	6383.2	35.94	66.92	50.316	33
	0.06	802.86	0.3768	6383.6	44.97	53.49	41.652	28.42
	0.06	855.66	0.3768	6383.6	42.19	57.01	47.319	20.48
	0.06	697.45	0.3768	6384.2	51.77	46.47	39.827	16.67
	0.06	735.68	0.3768	6384.1	49.08	49.01	42.083	16.47
	0.06	731.67	0.3768	6384.2	49.35	48.75	43.664	11.64
	0.06	681.29	0.3768	6384.3	53	45.39	41.113	10.4
	0.06	659.17	0.3768	6384.5	54.78	43.91	38.528	13.98
	0.06	201.22	0.3768	6384.7	59.82	40.22	33.452	20.22
	0.06	251.03	0.3768	6384	47.94	50.18	40.531	23.8
	0.06	177.35	0.3768	6384.8	67.87	35.45	30.489	16.26
	0.09	1211.5	0.5652	6383.2	39.73	90.81	67.476	34.58
	0.09	1353.71	0.5652	6383.4	35.56	101.5	75.979	33.54
	0.09	1081.46	0.5652	6383.8	44.51	81.06	63.84	26.98
	0.09	946.86	0.5652	6384.1	50.84	70.97	66.144	7.301
	0.09	1049.18	0.5652	6384.2	45.88	78.65	69.844	12.6
	0.09	1046.59	0.5652	6384.2	46	78.44	68.145	15.11
	0.09	233.93	0.5652	6384.1	51.45	70.13	58.003	20.91
	0.09	224.64	0.5652	6384.1	53.57	67.36	54.228	24.21
	0.09	196.58	0.5652	6384.5	61.22	58.94	49.774	18.42

		1						
	0.12	219.4	0.7536	6384	54.85	87.71	61.901	41.7
	0.12	325.42	0.7536	6383.2	36.98	130.1	95.918	35.62
	0.12	266.24	0.7536	6383.6	45.2	106.4	81.941	29.89
	0.12	235.36	0.7536	6383.8	51.13	94.09	75.799	24.13
	0.12	218.83	0.7536	6384.2	55	87.47	72.797	20.16

15h(right)

Y/m	X/m	resistiv	K	U	I	K*U/I	R	((K*U/I)-R)/R*100
	0.03	514.66	0.1884	6384.8	46.77	25.72	18.004	42.85
	0.03	651.43	0.1884	6384.5	36.95	32.55	23.918	36.11
	0.03	522.49	0.1884	6385.2	46.08	26.11	20.005	30.5
	0.03	454.23	0.1884	6385.5	53	22.7	18.873	20.27
	0.03	468.97	0.1884	6385.4	51.34	23.43	19.866	17.95
	0.03	486.85	0.1884	6385.5	49.45	24.33	20.493	18.72
	0.03	585.56	0.1884	6385.4	41.12	29.26	23.49	24.55
	0.03	387.36	0.1884	6386	62.16	19.36	16.946	14.22
	0.03	402.9	0.1884	6385.8	59.76	20.13	17.629	14.2
	0.03	390.96	0.1884	6386.1	61.59	19.53	18.993	2.851
	0.03	423.68	0.1884	6385.9	56.83	21.17	19.26	9.916
	0.03	398.44	0.1884	6386	60.43	19.91	17.588	13.2
	0.03	180.24	0.1884	6386	66.79	18.01	15.698	14.75
	0.03	232.73	0.1884	6385.7	51.73	23.26	20.773	11.96
	0.03	177.25	0.1884	6386.3	67.92	17.71	16.476	7.521
	0.06	738.03	0.3768	6385	48.93	49.17	35.817	37.28
	0.06	1013.95	0.3768	6384.7	35.61	67.56	50.316	34.27
	0.06	810.59	0.3768	6385.2	44.55	54.01	41.652	29.66
	0.06	864.62	0.3768	6385.2	41.77	57.6	47.319	21.73
	0.06	691.94	0.3768	6385.6	52.19	46.1	39.827	15.76
	0.06	729.76	0.3768	6385.6	49.49	48.62	42.083	15.53
	0.06	725.89	0.3768	6385.7	49.75	48.36	43.664	10.76
	0.06	677.31	0.3768	6385.8	53.32	45.13	41.113	9.762
	0.06	654.68	0.3768	6385.9	55.16	43.62	38.528	13.22
	0.06	198.44	0.3768	6386.2	60.67	39.66	33.452	18.57
	0.06	248.18	0.3768	6385.4	48.5	49.61	40.531	22.4
	0.06	175.82	0.3768	6386.2	68.47	35.14	30.489	15.27
	0.09	1223.43	0.5652	6384.7	39.35	91.71	67.476	35.91
	0.09	1367.01	0.5652	6384.7	35.22	102.5	75.979	34.85
	0.09	1097.11	0.5652	6385.3	43.89	82.23	63.84	28.8
	0.09	971.56	0.5652	6385.5	49.56	72.82	66.144	10.1
	0.09	1041.87	0.5652	6385.6	46.22	78.09	69.844	11.8
	0.09	1038.83	0.5652	6385.8	46.35	77.87	68.145	14.27
	0.09	232.2	0.5652	6385.7	51.84	69.62	58.003	20.03
	0.09	222.3	0.5652	6385.5	54.15	66.65	54.228	22.91
	0.09	195.01	0.5652	6386	61.73	58.47	49.774	17.47
		1						
	0.12	229.02	0.7536	6385.4	52.56	91.55	61.901	47.9
	0.12	328.54	0.7536	6384.6	36.64	131.3	95.918	36.91

	0.12	268.94	0.7536	6385.1	44.76	107.5	81.941	31.2
	0.12	240.46	0.7536	6385.3	50.06	96.12	75.799	26.81
	0.12	217.12	0.7536	6385.6	55.44	86.8	72.797	19.24

36h(right)

Y/m	X/m	resistiv	K	U	I	K*U/I	R	((K*U/I)-R)/R*100
	0.03	573.15	0.1884	6383.8	41.99	28.64	18.004	59.09
	0.03	647.57	0.1884	6383.8	37.17	32.36	23.918	35.29
	0.03	485.62	0.1884	6384.3	49.57	24.26	20.005	21.29
	0.03	440.39	0.1884	6384.4	54.66	22.01	18.873	16.6
	0.03	442.68	0.1884	6384.3	54.37	22.12	19.866	11.36
	0.03	463.68	0.1884	6384.3	51.91	23.17	20.493	13.07
	0.03	469.06	0.1884	6384.4	51.32	23.44	23.49	-0.22
	0.03	316.84	0.1884	6384.9	75.98	15.83	16.946	-6.57
	0.03	328.03	0.1884	6384.9	73.38	16.39	17.629	-7.01
	0.03	318.3	0.1884	6385.1	75.63	15.91	18.993	-16.3
	0.03	338.14	0.1884	6385	71.19	16.9	19.26	-12.3
	0.03	324.48	0.1884	6385.1	74.19	16.21	17.588	-7.81
	0.03	143.83	0.1884	6385.3	83.69	14.37	15.698	-8.43
	0.03	181.98	0.1884	6384.6	66.14	18.19	20.773	-12.4
	0.03	139.21	0.1884	6385.4	86.47	13.91	16.476	-15.6
	0.06	836.69	0.3768	6384	43.15	55.75	35.817	55.65
	0.06	1007.62	0.3768	6383.6	35.83	67.13	50.316	33.42
	0.06	746.51	0.3768	6384.2	48.37	49.73	41.652	19.4
	0.06	790.31	0.3768	6384.1	45.68	52.66	47.319	11.29
	0.06	656.13	0.3768	6384.5	55.03	43.72	39.827	9.765
	0.06	695.93	0.3768	6384.5	51.88	46.37	42.083	10.19
	0.06	588.4	0.3768	6384.9	61.37	39.2	43.664	-10.2
	0.06	547.28	0.3768	6384.9	65.98	36.46	41.113	-11.3
	0.06	540.27	0.3768	6385	66.84	35.99	38.528	-6.58
	0.06	161.87	0.3768	6385.4	74.36	32.36	33.452	-3.27
	0.06	198.14	0.3768	6384.6	60.74	39.61	40.531	-2.28
	0.06	142.07	0.3768	6385.5	84.73	28.4	30.489	-6.86
	0.09	1299.4	0.5652	6383.7	37.05	97.38	67.476	44.32
	0.09	1360.85	0.5652	6383.6	35.37	102	75.979	34.26
	0.09	1011.38	0.5652	6384.2	47.6	75.81	63.84	18.74
	0.09	934.45	0.5652	6384.5	51.52	70.04	66.144	5.892
	0.09	970.42	0.5652	6384.6	49.61	72.74	69.844	4.145
	0.09	996.26	0.5652	6384.6	48.32	74.68	68.145	9.592
	0.09	223.07	0.5652	6384.5	53.96	66.87	58.003	15.3
	0.09	181.42	0.5652	6384.6	66.34	54.39	54.228	0.308
	0.09	159.55	0.5652	6385.1	75.44	47.84	49.774	-3.89
		1						
	0.12	279.28	0.7536	6384.1	43.09	111.7	61.901	80.37
	0.12	342.15	0.7536	6383.6	35.17	136.8	95.918	42.6
	0.12	276.36	0.7536	6384.3	43.55	110.5	81.941	34.82
	0.12	230.17	0.7536	6384.7	52.29	92.02	75.799	21.4
	0.12	207.46	0.7536	6384.7	58.02	82.93	72.797	13.92

38h(right)

Y/m	X/m	resistiv	K	U	I	K*U/I	R	((K*U/I)-R)/R*100
	0.03	589.38	0.1884	6383.7	40.84	29.45	18.004	63.56
	0.03	609.08	0.1884	6383.9	39.52	30.43	23.918	27.24
	0.03	476.52	0.1884	6384.3	50.51	23.81	20.005	19.04
	0.03	429.08	0.1884	6384.3	56.1	21.44	18.873	13.61
	0.03	441.01	0.1884	6384	54.58	22.04	19.866	10.92
	0.03	466.79	0.1884	6384.1	51.57	23.32	20.493	13.81
	0.03	444.44	0.1884	6384.3	54.16	22.21	23.49	-5.46
	0.03	292.03	0.1884	6384.9	82.43	14.59	16.946	-13.9
	0.03	317.18	0.1884	6384.7	75.89	15.85	17.629	-10.1
	0.03	306.85	0.1884	6384.8	78.45	15.33	18.993	-19.3
	0.03	328.87	0.1884	6384.6	73.19	16.43	19.26	-14.7
	0.03	313.79	0.1884	6384.9	76.72	15.68	17.588	-10.9
	0.03	137.13	0.1884	6385.2	87.78	13.7	15.698	-12.7
	0.03	172.16	0.1884	6384.5	69.91	17.21	20.773	-17.2
	0.03	135.93	0.1884	6385.3	88.55	13.59	16.476	-17.5
	0.06	866.2	0.3768	6384	41.68	57.71	35.817	61.13
	0.06	968.04	0.3768	6383.6	37.3	64.49	50.316	28.16
	0.06	729.16	0.3768	6384	49.52	48.58	41.652	16.63
	0.06	757.48	0.3768	6384	47.66	50.47	47.319	6.661
	0.06	655.82	0.3768	6384.3	55.05	43.7	39.827	9.722
	0.06	703.6	0.3768	6384.3	51.32	46.87	42.083	11.39
	0.06	562.39	0.3768	6384.8	64.2	37.47	43.664	-14.2
	0.06	514.53	0.3768	6384.6	70.18	34.28	41.113	-16.6
	0.06	526.44	0.3768	6384.8	68.59	35.08	38.528	-8.96
	0.06	155.28	0.3768	6385.2	77.52	31.04	33.452	-7.22
	0.06	189.16	0.3768	6384.4	63.63	37.81	40.531	-6.72
	0.06	137	0.3768	6385.3	87.86	27.38	30.489	-10.2
	0.09	1317.73	0.5652	6383.8	36.53	98.77	67.476	46.38
	0.09	1307.95	0.5652	6383.5	36.8	98.04	75.979	29.04
	0.09	988.12	0.5652	6384	48.72	74.06	63.84	16.01
	0.09	902.14	0.5652	6384.5	53.36	67.63	66.144	2.24
	0.09	969.96	0.5652	6384.3	49.63	72.71	69.844	4.099
	0.09	1008.53	0.5652	6384.4	47.74	75.59	68.145	10.92
	0.09	225.6	0.5652	6384.2	53.35	67.64	58.003	16.61
	0.09	174.75	0.5652	6384.4	68.87	52.4	54.228	-3.38
	0.09	145.67	0.5652	6385.1	82.63	43.67	49.774	-12.3
		1						
	0.12	285.13	0.7536	6383.9	42.21	114	61.901	84.12
	0.12	329.87	0.7536	6383.5	36.48	131.9	95.918	37.48
	0.12	278.04	0.7536	6384.1	43.29	111.1	81.941	35.63
	0.12	222.22	0.7536	6384.8	54.16	88.84	75.799	17.21
	0.12	199.88	0.7536	6384.7	60.22	79.9	72.797	9.755

FIG7-1 48h(left)

Y/m	X/m	resistiv	K	U	I	K*U/I	R	((K*U/I)-R)/R*100
	0.03	593.02	0.1884	6384.1	40.59	29.63	16.046	84.67
	0.03	620.66	0.1884	6383.5	38.78	31.01	20.039	54.76
	0.03	445.71	0.1884	6383.9	54	22.27	28.302	-21.3
	0.03	395.17	0.1884	6384.7	60.92	19.75	17.607	12.14
	0.03	347.75	0.1884	6384.5	69.22	17.38	16.974	2.374
	0.03	371.43	0.1884	6384.5	64.81	18.56	16.566	12.03
	0.03	351.62	0.1884	6384.5	68.46	17.57	18.296	-3.97
	0.03	304.98	0.1884	6384.8	78.93	15.24	17.332	-12.1
	0.03	280.38	0.1884	6385	85.86	14.01	17.142	-18.3
	0.03	300.85	0.1884	6385	80.02	15.03	18.151	-17.2
	0.03	266.23	0.1884	6385.2	90.42	13.3	15.501	-14.2
	0.03	282.93	0.1884	6385.1	85.08	14.14	16.273	-13.1
	0.03	130.6	0.1884	6385	92.16	13.05	16.423	-20.5
	0.03	135.38	0.1884	6385.2	88.91	13.53	17.014	-20.5
	0.03	113.22	0.1884	6385.1	106.3	11.32	13.807	-18
	0.06	841.19	0.3768	6383.6	42.92	56.04	31.931	75.51
	0.06	922.87	0.3768	6383.6	39.12	61.49	40.475	51.91
	0.06	703.99	0.3768	6383.8	51.28	46.91	60.978	-23.1
	0.06	625.03	0.3768	6384.2	57.77	41.64	39.064	6.596
	0.06	540.54	0.3768	6384.6	66.8	36.01	36.383	-1.02
	0.06	579.25	0.3768	6384.6	62.33	38.6	36.818	4.831
	0.06	581.69	0.3768	6384.7	62.07	38.76	41.256	-6.05
	0.06	477.71	0.3768	6385.1	75.59	31.83	35.705	-10.9
	0.06	449.51	0.3768	6385.3	80.33	29.95	33.804	-11.4
	0.06	130.62	0.3768	6385	92.15	26.11	29.834	-12.5
	0.06	130.49	0.3768	6385.2	92.24	26.08	30.955	-15.7
	0.06	124.87	0.3768	6385.5	96.4	24.96	29.477	-15.3
	0.09	1181.22	0.5652	6383.6	40.75	88.54	53.929	64.18
	0.09	1275.31	0.5652	6383.6	37.75	95.58	63.94	49.48
	0.09	973.79	0.5652	6384	49.43	73	95.717	-23.7
	0.09	909.04	0.5652	6384.3	52.96	68.13	65.31	4.325
	0.09	751.97	0.5652	6384.7	64.02	56.37	56.368	-0
	0.09	811.08	0.5652	6384.7	59.36	60.79	54.836	10.86
	0.09	167.93	0.5652	6384.9	71.68	50.35	42.232	19.21
	0.09	161.9	0.5652	6384.8	74.34	48.54	47.961	1.213
	0.09	144.44	0.5652	6385	83.33	43.31	47.266	-8.37
		1						
	0.12	300.49	0.7536	6382.9	40.05	120.1	56.345	113.2
	0.12	310.65	0.7536	6384.8	38.75	124.2	71.899	72.7
	0.12	249.1	0.7536	6383.5	48.31	99.58	105.58	-5.69
	0.12	211.14	0.7536	6384.5	57	84.41	69.829	20.88
	0.12	172.97	0.7536	6385.1	69.59	69.14	65.702	5.24

49h(left)

Y/m	X/m	resistiv	K	U	I	K*U/I	R	((K*U/I)-R)/R*100
	0.03	593.02	0.1884	6384.1	40.59	29.63	16.046	84.67
	0.03	620.66	0.1884	6383.5	38.78	31.01	20.039	54.76
	0.03	445.71	0.1884	6383.9	54	22.27	28.302	-21.3
	0.03	395.17	0.1884	6384.7	60.92	19.75	17.607	12.14
	0.03	347.75	0.1884	6384.5	69.22	17.38	16.974	2.374
	0.03	371.43	0.1884	6384.5	64.81	18.56	16.566	12.03
	0.03	351.62	0.1884	6384.5	68.46	17.57	18.296	-3.97
	0.03	304.98	0.1884	6384.8	78.93	15.24	17.332	-12.1
	0.03	280.38	0.1884	6385	85.86	14.01	17.142	-18.3
	0.03	300.85	0.1884	6385	80.02	15.03	18.151	-17.2
	0.03	266.23	0.1884	6385.2	90.42	13.3	15.501	-14.2
	0.03	282.93	0.1884	6385.1	85.08	14.14	16.273	-13.1
	0.03	130.6	0.1884	6385	92.16	13.05	16.423	-20.5
	0.03	135.38	0.1884	6385.2	88.91	13.53	17.014	-20.5
	0.03	113.22	0.1884	6385.1	106.3	11.32	13.807	-18
	0.06	841.19	0.3768	6383.6	42.92	56.04	31.931	75.51
	0.06	922.87	0.3768	6383.6	39.12	61.49	40.475	51.91
	0.06	703.99	0.3768	6383.8	51.28	46.91	60.978	-23.1
	0.06	625.03	0.3768	6384.2	57.77	41.64	39.064	6.596
	0.06	540.54	0.3768	6384.6	66.8	36.01	36.383	-1.02
	0.06	579.25	0.3768	6384.6	62.33	38.6	36.818	4.831
	0.06	581.69	0.3768	6384.7	62.07	38.76	41.256	-6.05
	0.06	477.71	0.3768	6385.1	75.59	31.83	35.705	-10.9
	0.06	449.51	0.3768	6385.3	80.33	29.95	33.804	-11.4
	0.06	130.62	0.3768	6385	92.15	26.11	29.834	-12.5
	0.06	130.49	0.3768	6385.2	92.24	26.08	30.955	-15.7
	0.06	124.87	0.3768	6385.5	96.4	24.96	29.477	-15.3
	0.09	1181.22	0.5652	6383.6	40.75	88.54	53.929	64.18
	0.09	1275.31	0.5652	6383.6	37.75	95.58	63.94	49.48
	0.09	973.79	0.5652	6384	49.43	73	95.717	-23.7
	0.09	909.04	0.5652	6384.3	52.96	68.13	65.31	4.325
	0.09	751.97	0.5652	6384.7	64.02	56.37	56.368	-0
	0.09	811.08	0.5652	6384.7	59.36	60.79	54.836	10.86
	0.09	167.93	0.5652	6384.9	71.68	50.35	42.232	19.21
	0.09	161.9	0.5652	6384.8	74.34	48.54	47.961	1.213
	0.09	144.44	0.5652	6385	83.33	43.31	47.266	-8.37
		1						
	0.12	300.49	0.7536	6382.9	40.05	120.1	56.345	113.2
	0.12	310.65	0.7536	6384.8	38.75	124.2	71.899	72.7
	0.12	249.1	0.7536	6383.5	48.31	99.58	105.58	-5.69
	0.12	211.14	0.7536	6384.5	57	84.41	69.829	20.88
	0.12	172.97	0.7536	6385.1	69.59	69.14	65.702	5.24

50h(left)

Y/m	X/m	resistiv	K	U	I	K*U/I	R	((K*U/I)-R)/R*100
	0.03	642.96	0.1884	6384	37.44	32.12	16.046	100.2
	0.03	593.71	0.1884	6383.5	40.54	29.67	20.039	48.04
	0.03	436.92	0.1884	6383.9	55.09	21.83	28.302	-22.9
	0.03	388.78	0.1884	6384.6	61.91	19.43	17.607	10.35
	0.03	340.35	0.1884	6384.6	70.73	17.01	16.974	0.189
	0.03	363.77	0.1884	6384.5	66.17	18.18	16.566	9.731
	0.03	344.52	0.1884	6384.6	69.87	17.22	18.296	-5.91
	0.03	296.61	0.1884	6384.9	81.16	14.82	17.332	-14.5
	0.03	274.73	0.1884	6385.2	87.62	13.73	17.142	-19.9
	0.03	295.21	0.1884	6385.2	81.55	14.75	18.151	-18.7
	0.03	260.52	0.1884	6385.2	92.4	13.02	15.501	-16
	0.03	277.36	0.1884	6385.1	86.79	13.86	16.273	-14.8
	0.03	127.84	0.1884	6385.1	94.16	12.78	16.423	-22.2
	0.03	133.11	0.1884	6385.3	90.43	13.3	17.014	-21.8
	0.03	110.64	0.1884	6385.2	108.8	11.06	13.807	-19.9
	0.06	898.08	0.3768	6383.6	40.2	59.83	31.931	87.39
	0.06	880.37	0.3768	6383.7	41.01	58.65	40.475	44.91
	0.06	687.33	0.3768	6383.8	52.53	45.79	60.978	-24.9
	0.06	614.67	0.3768	6384.3	58.74	40.95	39.064	4.838
	0.06	528.63	0.3768	6384.5	68.3	35.22	36.383	-3.19
	0.06	567.71	0.3768	6384.6	63.6	37.83	36.818	2.737
	0.06	572.01	0.3768	6384.8	63.13	38.11	41.256	-7.63
	0.06	465.23	0.3768	6385	77.62	31	35.705	-13.2
	0.06	441.49	0.3768	6385.4	81.79	29.42	33.804	-13
	0.06	127.53	0.3768	6385.1	94.39	25.49	29.834	-14.6
	0.06	127.16	0.3768	6385.3	94.66	25.42	30.955	-17.9
	0.06	121.88	0.3768	6385.5	98.76	24.36	29.477	-17.4
	0.09	1238.02	0.5652	6383.6	38.88	92.8	53.929	72.08
	0.09	1203.38	0.5652	6383.6	40	90.2	63.94	41.07
	0.09	946.27	0.5652	6383.9	50.87	70.93	95.717	-25.9
	0.09	895.92	0.5652	6384.3	53.73	67.16	65.31	2.831
	0.09	735.27	0.5652	6384.9	65.48	55.11	56.368	-2.23
	0.09	795.51	0.5652	6384.8	60.52	59.63	54.836	8.738
	0.09	164.46	0.5652	6384.9	73.19	49.31	42.232	16.75
	0.09	158.41	0.5652	6384.9	75.98	47.5	47.961	-0.97
	0.09	140.51	0.5652	6385.2	85.67	42.13	47.266	-10.9
	0.12	314.72	0.7536	6085.3	36.45	125.8	56.345	123.3
	0.12	298.51	0.7536	6384.9	40.32	119.3	71.899	65.98
	0.12	244.6	0.7536	6383.5	49.2	97.78	105.58	-7.39
	0.12	208.3	0.7536	6384.5	57.78	83.27	69.829	19.25
	0.12	168.33	0.7536	6385.1	71.51	67.29	65.702	2.415

52h(left)

Y/m	X/m	resistiv	K	U	I	K*U/I	R	((K*U/I)-R)/R*100
	0.03	682.37	0.1884	6383.6	35.27	34.1	16.046	112.5
	0.03	623.99	0.1884	6383.1	38.57	31.18	20.039	55.59
	0.03	449.42	0.1884	6383.5	53.55	22.46	28.302	-20.6
	0.03	389.31	0.1884	6384.2	61.83	19.45	17.607	10.48
	0.03	339.76	0.1884	6384.3	70.84	16.98	16.974	0.029
	0.03	361.13	0.1884	6384.1	66.65	18.05	16.566	8.934
	0.03	348.01	0.1884	6384.1	69.16	17.39	18.296	-4.95
	0.03	292.38	0.1884	6384.6	82.33	14.61	17.332	-15.7
	0.03	270.63	0.1884	6384.8	88.95	13.52	17.142	-21.1
	0.03	291.38	0.1884	6384.6	82.61	14.56	18.151	-19.8
	0.03	258.41	0.1884	6384.8	93.15	12.91	15.501	-16.7
	0.03	275.06	0.1884	6384.8	87.51	13.75	16.273	-15.5
	0.03	124.84	0.1884	6384.7	96.41	12.48	16.423	-24
	0.03	130.72	0.1884	6384.9	92.08	13.06	17.014	-23.2
	0.03	109.56	0.1884	6384.8	109.9	10.95	13.807	-20.7
	0.06	974.85	0.3768	6383.3	37.03	64.95	31.931	103.4
	0.06	933.83	0.3768	6383.3	38.66	62.22	40.475	53.71
	0.06	708.56	0.3768	6383.4	50.95	47.21	60.978	-22.6
	0.06	614.49	0.3768	6383.9	58.75	40.94	39.064	4.814
	0.06	527.65	0.3768	6384.2	68.43	35.15	36.383	-3.38
	0.06	562.38	0.3768	6384.1	64.2	37.47	36.818	1.77
	0.06	576.9	0.3768	6384.3	62.59	38.43	41.256	-6.84
	0.06	460.91	0.3768	6384.6	78.34	30.71	35.705	-14
	0.06	439.34	0.3768	6384.7	82.18	29.27	33.804	-13.4
	0.06	125.86	0.3768	6384.7	95.63	25.16	29.834	-15.7
	0.06	125.28	0.3768	6384.9	96.08	25.04	30.955	-19.1
	0.06	120.18	0.3768	6385.1	100.2	24.02	29.477	-18.5
	0.09	1390.27	0.5652	6383.2	34.62	104.2	53.929	93.24
	0.09	1288.79	0.5652	6383.2	37.35	96.59	63.94	51.07
	0.09	978.65	0.5652	6383.6	49.19	73.35	95.717	-23.4
	0.09	894.45	0.5652	6384.1	53.82	67.04	65.31	2.654
	0.09	734.57	0.5652	6384.5	65.54	55.06	56.368	-2.32
	0.09	792.18	0.5652	6384.3	60.77	59.38	54.836	8.283
	0.09	163.61	0.5652	6384.5	73.57	49.05	42.232	16.14
	0.09	160.47	0.5652	6384.5	75	48.11	47.961	0.318
	0.09	138.22	0.5652	6384.8	87.08	41.44	47.266	-12.3
		1						
	0.12	322.83	0.7536	5982.8	34.94	129	56.345	129
	0.12	313.52	0.7536	6384.4	38.39	125.3	71.899	74.31
	0.12	250.04	0.7536	6383.1	48.13	99.94	105.58	-5.34
	0.12	212.36	0.7536	6384.2	56.67	84.9	69.829	21.58
	0.12	167.74	0.7536	6384.7	71.75	67.06	65.702	2.066
55h(left)								

Y/m	X/m	resistiv	K	U	I	K*U/I	R	((K*U/I)-R)/R*100
	0.03	641.76	0.1884	6383	37.5	32.07	16.046	99.86
	0.03	618.72	0.1884	6382.4	38.89	30.92	20.039	54.3
	0.03	446.4	0.1884	6383	53.91	22.31	28.302	-21.2
	0.03	392.04	0.1884	6383.6	61.39	19.59	17.607	11.26
	0.03	343.18	0.1884	6383.7	70.13	17.15	16.974	1.033
	0.03	364	0.1884	6383.7	66.12	18.19	16.566	9.801
	0.03	345.32	0.1884	6383.7	69.7	17.26	18.296	-5.69
	0.03	284.54	0.1884	6383.9	84.59	14.22	17.332	-18
	0.03	267.99	0.1884	6384.3	89.82	13.39	17.142	-21.9
	0.03	289.19	0.1884	6384.2	83.23	14.45	18.151	-20.4
	0.03	255.59	0.1884	6384.3	94.17	12.77	15.501	-17.6
	0.03	271.93	0.1884	6384.3	88.52	13.59	16.273	-16.5
	0.03	123.35	0.1884	6384.2	97.57	12.33	16.423	-24.9
	0.03	129.59	0.1884	6384.5	92.88	12.95	17.014	-23.9
	0.03	108.44	0.1884	6384.4	111	10.84	13.807	-21.5
	0.06	890.39	0.3768	6382.6	40.54	59.32	31.931	85.79
	0.06	918.89	0.3768	6382.6	39.28	61.23	40.475	51.27
	0.06	699.73	0.3768	6382.8	51.59	46.62	60.978	-23.5
	0.06	617.75	0.3768	6383.4	58.44	41.16	39.064	5.36
	0.06	532.21	0.3768	6383.6	67.83	35.46	36.383	-2.53
	0.06	567.2	0.3768	6383.6	63.65	37.79	36.818	2.641
	0.06	573.35	0.3768	6383.8	62.97	38.2	41.256	-7.41
	0.06	449.76	0.3768	6384.2	80.27	29.97	35.705	-16.1
	0.06	435.32	0.3768	6384.4	82.94	29	33.804	-14.2
	0.06	124.01	0.3768	6384.2	97.05	24.79	29.834	-16.9
	0.06	123.64	0.3768	6384.3	97.34	24.71	30.955	-20.2
	0.06	118.69	0.3768	6384.4	101.4	23.72	29.477	-19.5
	0.09	1219.94	0.5652	6382.6	39.45	91.44	53.929	69.56
	0.09	1255.69	0.5652	6382.5	38.33	94.11	63.94	47.19
	0.09	957.8	0.5652	6383	50.25	71.79	95.717	-25
	0.09	900.4	0.5652	6383.4	53.46	67.49	65.31	3.335
	0.09	740.15	0.5652	6383.9	65.04	55.48	56.368	-1.58
	0.09	797.27	0.5652	6383.9	60.38	59.76	54.836	8.974
	0.09	165.51	0.5652	6384.1	72.71	49.63	42.232	17.51
	0.09	159.59	0.5652	6383.9	75.41	47.85	47.961	-0.24
	0.09	134.32	0.5652	6384.4	89.6	40.27	47.266	-14.8
		1						
	0.12	316.64	0.7536	6159.4	36.67	126.6	56.345	124.7
	0.12	316.88	0.7536	6383.9	37.98	126.7	71.899	76.18
	0.12	252.36	0.7536	6382.5	47.68	100.9	105.58	-4.46
	0.12	214.51	0.7536	6383.7	56.1	85.75	69.829	22.81
	0.12	167.55	0.7536	6384.1	71.83	66.98	65.702	1.944

58h(left)

Y/m	X/m	resistivity	K	U	I	K*U/I	R	((K*U/I)-R)/R*100
	0.03	681.59	0.1884	6384.1	35.32	34.05	16.046	112.2
	0.03	638.79	0.1884	6383.5	37.68	31.92	20.039	59.28
	0.03	457.09	0.1884	6383.9	52.66	22.84	28.302	-19.3
	0.03	389.92	0.1884	6384.7	61.74	19.48	17.607	10.65
	0.03	342.96	0.1884	6384.7	70.19	17.14	16.974	0.961
	0.03	360.94	0.1884	6384.6	66.69	18.04	16.566	8.878
	0.03	344.9	0.1884	6384.6	69.79	17.24	18.296	-5.8
	0.03	280.66	0.1884	6385.2	85.77	14.03	17.332	-19.1
	0.03	264.7	0.1884	6385.3	90.95	13.23	17.142	-22.8
	0.03	284.13	0.1884	6385.3	84.73	14.2	18.151	-21.8
	0.03	252.14	0.1884	6385.4	95.48	12.6	15.501	-18.7
	0.03	268.98	0.1884	6385.5	89.5	13.44	16.273	-17.4
	0.03	121.52	0.1884	6385.2	99.05	12.15	16.423	-26
	0.03	126.71	0.1884	6385.6	95	12.66	17.014	-25.6
	0.03	106.91	0.1884	6385.6	112.6	10.68	13.807	-22.6
	0.06	967.98	0.3768	6383.7	37.3	64.49	31.931	102
	0.06	955.66	0.3768	6383.7	37.78	63.67	40.475	57.3
	0.06	719.9	0.3768	6384	50.15	47.97	60.978	-21.3
	0.06	614.29	0.3768	6384.5	58.78	40.93	39.064	4.77
	0.06	532.54	0.3768	6384.6	67.8	35.48	36.383	-2.48
	0.06	563.09	0.3768	6384.8	64.12	37.52	36.818	1.908
	0.06	571.96	0.3768	6384.9	63.13	38.11	41.256	-7.63
	0.06	445.17	0.3768	6385.3	81.12	29.66	35.705	-16.9
	0.06	431.21	0.3768	6385.4	83.74	28.73	33.804	-15
	0.06	122.22	0.3768	6385.3	98.49	24.43	29.834	-18.1
	0.06	121.59	0.3768	6385.5	99	24.3	30.955	-21.5
	0.06	116.63	0.3768	6385.3	103.2	23.31	29.477	-20.9
	0.09	1380.85	0.5652	6383.6	34.86	103.5	53.929	91.92
	0.09	1325.21	0.5652	6383.7	36.33	99.31	63.94	55.32
	0.09	993.44	0.5652	6384.2	48.46	74.46	95.717	-22.2
	0.09	894.35	0.5652	6384.5	53.83	67.04	65.31	2.642
	0.09	741.24	0.5652	6385	64.95	55.56	56.368	-1.43
	0.09	792.2	0.5652	6385	60.78	59.38	54.836	8.277
	0.09	164.17	0.5652	6385.1	73.32	49.22	42.232	16.55
	0.09	159.61	0.5652	6385	75.41	47.86	47.961	-0.22
	0.09	131.96	0.5652	6385.5	91.22	39.56	47.266	-16.3
		1						
	0.12	322.13	0.7536	5944.7	34.79	128.8	56.345	128.5
	0.12	327.98	0.7536	6384.9	36.7	131.1	71.899	82.35
	0.12	257.42	0.7536	6383.7	46.75	102.9	105.58	-2.54
	0.12	215.42	0.7536	6384.8	55.87	86.12	69.829	23.33
	0.12	167.83	0.7536	6385.2	71.72	67.09	65.702	2.118

70h(left)

Y/m	X/m	resistivity	K	U	I	K*U/I	R	((K*U/I)-R)/R*100
	0.03	669.73	0.1884	6383.2	35.94	33.46	16.046	108.5
	0.03	633.49	0.1884	6382.7	37.99	31.65	20.039	57.96
	0.03	451.12	0.1884	6383.1	53.35	22.54	28.302	-20.4
	0.03	390.66	0.1884	6383.8	61.61	19.52	17.607	10.87
	0.03	345.07	0.1884	6383.8	69.75	17.24	16.974	1.584
	0.03	363.32	0.1884	6383.7	66.25	18.15	16.566	9.585
	0.03	346.29	0.1884	6383.8	69.5	17.31	18.296	-5.42
	0.03	279.22	0.1884	6384.4	86.21	13.95	17.332	-19.5
	0.03	263.7	0.1884	6384.6	91.28	13.18	17.142	-23.1
	0.03	283.28	0.1884	6384.4	84.97	14.16	18.151	-22
	0.03	251.15	0.1884	6384.5	95.84	12.55	15.501	-19
	0.03	268.2	0.1884	6384.6	89.75	13.4	16.273	-17.6
	0.03	120.98	0.1884	6384.4	99.48	12.09	16.423	-26.4
	0.03	126.42	0.1884	6384.8	95.21	12.63	17.014	-25.7
	0.03	106.49	0.1884	6384.6	113	10.64	13.807	-22.9
	0.06	932.68	0.3768	6382.9	38.7	62.15	31.931	94.63
	0.06	944.44	0.3768	6382.8	38.22	62.93	40.475	55.47
	0.06	707.42	0.3768	6383.1	51.03	47.13	60.978	-22.7
	0.06	615.56	0.3768	6383.6	58.65	41.01	39.064	4.987
	0.06	536.26	0.3768	6383.7	67.32	35.73	36.383	-1.79
	0.06	566.99	0.3768	6383.9	63.68	37.77	36.818	2.598
	0.06	574.17	0.3768	6384	62.88	38.26	41.256	-7.27
	0.06	443.47	0.3768	6384.4	81.42	29.55	35.705	-17.2
	0.06	430.2	0.3768	6384.6	83.93	28.66	33.804	-15.2
	0.06	121.5	0.3768	6384.5	99.06	24.29	29.834	-18.6
	0.06	120.96	0.3768	6384.6	99.5	24.18	30.955	-21.9
	0.06	115.88	0.3768	6384.6	103.9	23.16	29.477	-21.4
	0.09	1286.67	0.5652	6382.8	37.41	96.43	53.929	78.81
	0.09	1286.57	0.5652	6382.6	37.41	96.43	63.94	50.81
	0.09	972.48	0.5652	6383.1	49.49	72.9	95.717	-23.8
	0.09	896.64	0.5652	6383.6	53.68	67.21	65.31	2.915
	0.09	746.39	0.5652	6384.1	64.5	55.94	56.368	-0.76
	0.09	797.94	0.5652	6384.1	60.33	59.81	54.836	9.068
	0.09	165.27	0.5652	6384.2	72.82	49.55	42.232	17.33
	0.09	160.22	0.5652	6384.1	75.11	48.04	47.961	0.163
	0.09	131.03	0.5652	6384.6	91.86	39.28	47.266	-16.9
		1						
	0.12	319.73	0.7536	5967.5	35.19	127.8	56.345	126.8
	0.12	327.65	0.7536	6383.9	36.73	131	71.899	82.17
	0.12	256.77	0.7536	6382.7	46.86	102.6	105.58	-2.78
	0.12	216.78	0.7536	6383.9	55.52	86.65	69.829	24.09
	0.12	168.79	0.7536	6384.4	71.3	67.48	65.702	2.706

48h(right)

Y/m	X/m	resistivity	K	U	I	K*U/I	R	((K*U/I)-R)/R*100
	0.03	589.38	0.1884	6383.7	40.84	29.45	18.004	63.56
	0.03	609.08	0.1884	6383.9	39.52	30.43	23.918	27.24
	0.03	476.52	0.1884	6384.3	50.51	23.81	20.005	19.04
	0.03	429.08	0.1884	6384.3	56.1	21.44	18.873	13.61
	0.03	441.01	0.1884	6384	54.58	22.04	19.866	10.92
	0.03	466.79	0.1884	6384.1	51.57	23.32	20.493	13.81
	0.03	444.44	0.1884	6384.3	54.16	22.21	23.49	-5.46
	0.03	292.03	0.1884	6384.9	82.43	14.59	16.946	-13.9
	0.03	317.18	0.1884	6384.7	75.89	15.85	17.629	-10.1
	0.03	306.85	0.1884	6384.8	78.45	15.33	18.993	-19.3
	0.03	328.87	0.1884	6384.6	73.19	16.43	19.26	-14.7
	0.03	313.79	0.1884	6384.9	76.72	15.68	17.588	-10.9
	0.03	137.13	0.1884	6385.2	87.78	13.7	15.698	-12.7
	0.03	172.16	0.1884	6384.5	69.91	17.21	20.773	-17.2
	0.03	135.93	0.1884	6385.3	88.55	13.59	16.476	-17.5
	0.06	866.2	0.3768	6384	41.68	57.71	35.817	61.13
	0.06	968.04	0.3768	6383.6	37.3	64.49	50.316	28.16
	0.06	729.16	0.3768	6384	49.52	48.58	41.652	16.63
	0.06	757.48	0.3768	6384	47.66	50.47	47.319	6.661
	0.06	655.82	0.3768	6384.3	55.05	43.7	39.827	9.722
	0.06	703.6	0.3768	6384.3	51.32	46.87	42.083	11.39
	0.06	562.39	0.3768	6384.8	64.2	37.47	43.664	-14.2
	0.06	514.53	0.3768	6384.6	70.18	34.28	41.113	-16.6
	0.06	526.44	0.3768	6384.8	68.59	35.08	38.528	-8.96
	0.06	155.28	0.3768	6385.2	77.52	31.04	33.452	-7.22
	0.06	189.16	0.3768	6384.4	63.63	37.81	40.531	-6.72
	0.06	137	0.3768	6385.3	87.86	27.38	30.489	-10.2
	0.09	1317.73	0.5652	6383.8	36.53	98.77	67.476	46.38
	0.09	1307.95	0.5652	6383.5	36.8	98.04	75.979	29.04
	0.09	988.12	0.5652	6384	48.72	74.06	63.84	16.01
	0.09	902.14	0.5652	6384.5	53.36	67.63	66.144	2.24
	0.09	969.96	0.5652	6384.3	49.63	72.71	69.844	4.099
	0.09	1008.53	0.5652	6384.4	47.74	75.59	68.145	10.92
	0.09	225.6	0.5652	6384.2	53.35	67.64	58.003	16.61
	0.09	174.75	0.5652	6384.4	68.87	52.4	54.228	-3.38
	0.09	145.67	0.5652	6385.1	82.63	43.67	49.774	-12.3
		1						
	0.12	285.13	0.7536	6383.9	42.21	114	61.901	84.12
	0.12	329.87	0.7536	6383.5	36.48	131.9	95.918	37.48
	0.12	278.04	0.7536	6384.1	43.29	111.1	81.941	35.63
	0.12	222.22	0.7536	6384.8	54.16	88.84	75.799	17.21
	0.12	199.88	0.7536	6384.7	60.22	79.9	72.797	9.755

49h(right)

Y/m	X/m	resistivity	K	U	I	K*U/I	R	((K*U/I)-R)/R*100
	0.03	575.37	0.1884	6383.4	41.83	28.75	18.004	59.69
	0.03	587.65	0.1884	6383.7	40.96	29.36	23.918	22.77
	0.03	465.31	0.1884	6384.4	51.73	23.25	20.005	16.23
	0.03	421.29	0.1884	6384.2	57.14	21.05	18.873	11.54
	0.03	434.16	0.1884	6384	55.44	21.69	19.866	9.202
	0.03	459.41	0.1884	6384.1	52.39	22.96	20.493	12.03
	0.03	436.86	0.1884	6384.3	55.1	21.83	23.49	-7.07
	0.03	287.33	0.1884	6385.1	83.78	14.36	16.946	-15.3
	0.03	312.36	0.1884	6384.6	77.06	15.61	17.629	-11.5
	0.03	301.71	0.1884	6384.9	79.79	15.08	18.993	-20.6
	0.03	322.98	0.1884	6384.8	74.53	16.14	19.26	-16.2
	0.03	308.98	0.1884	6384.9	77.91	15.44	17.588	-12.2
	0.03	135.01	0.1884	6385.3	89.16	13.49	15.698	-14
	0.03	168.97	0.1884	6384.6	71.23	16.89	20.773	-18.7
	0.03	133.49	0.1884	6385.3	90.18	13.34	16.476	-19
	0.06	842.04	0.3768	6383.8	42.88	56.1	35.817	56.62
	0.06	926.28	0.3768	6383.6	38.98	61.71	50.316	22.64
	0.06	709.43	0.3768	6383.9	50.89	47.27	41.652	13.48
	0.06	741.95	0.3768	6383.7	48.66	49.43	47.319	4.466
	0.06	645.67	0.3768	6384.3	55.92	43.02	39.827	8.014
	0.06	692.08	0.3768	6384.3	52.17	46.11	42.083	9.571
	0.06	551.96	0.3768	6384.6	65.42	36.77	43.664	-15.8
	0.06	504.21	0.3768	6384.8	71.61	33.6	41.113	-18.3
	0.06	518.76	0.3768	6384.8	69.6	34.57	38.528	-10.3
	0.06	153.05	0.3768	6385.1	78.65	30.59	33.452	-8.55
	0.06	186.08	0.3768	6384.4	64.68	37.19	40.531	-8.24
	0.06	135.11	0.3768	6385.3	89.09	27.01	30.489	-11.4
	0.09	1273.13	0.5652	6383.5	37.81	95.42	67.476	41.42
	0.09	1241.45	0.5652	6383.6	38.78	93.04	75.979	22.45
	0.09	955.33	0.5652	6384.1	50.39	71.61	63.84	12.17
	0.09	881.99	0.5652	6384.3	54.58	66.11	66.144	-0.05
	0.09	951.98	0.5652	6384.3	50.57	71.35	69.844	2.162
	0.09	991.54	0.5652	6384.3	48.55	74.32	68.145	9.068
	0.09	222.02	0.5652	6384.3	54.21	66.56	58.003	14.76
	0.09	172.06	0.5652	6384.4	69.95	51.59	54.228	-4.87
	0.09	143.37	0.5652	6385	83.95	42.99	49.774	-13.6
		1						
	0.12	279.55	0.7536	6383.7	43.05	111.7	61.901	80.53
	0.12	319.97	0.7536	6383.5	37.61	127.9	95.918	33.35
	0.12	271.8	0.7536	6384	44.28	108.6	81.941	32.59
	0.12	218.01	0.7536	6384.6	55.21	87.15	75.799	14.97
	0.12	196.42	0.7536	6384.6	61.28	78.52	72.797	7.855

50h(right)

Y/m	X/m	resistivity	K	U	I	K*U/I	R	((K*U/I)-R)/R*100
	0.03	656.12	0.1884	6383.7	36.68	32.79	18.004	82.12
	0.03	593.18	0.1884	6384.1	40.58	29.64	23.918	23.92
	0.03	470.38	0.1884	6384.7	51.18	23.5	20.005	17.48
	0.03	411.31	0.1884	6384.5	58.52	20.55	18.873	8.911
	0.03	424.64	0.1884	6384.4	56.69	21.22	19.866	6.8
	0.03	443.77	0.1884	6384.5	54.24	22.18	20.493	8.215
	0.03	418.63	0.1884	6384.7	57.5	20.92	23.49	-10.9
	0.03	278.56	0.1884	6385.3	86.42	13.92	16.946	-17.9
	0.03	305.63	0.1884	6385.1	78.77	15.27	17.629	-13.4
	0.03	293.27	0.1884	6385.2	82.09	14.65	18.993	-22.8
	0.03	312.4	0.1884	6385.1	77.06	15.61	19.26	-18.9
	0.03	299.31	0.1884	6385.2	80.43	14.96	17.588	-15
	0.03	130.05	0.1884	6385.6	92.57	13	15.698	-17.2
	0.03	161.41	0.1884	6384.9	74.57	16.13	20.773	-22.3
	0.03	128.36	0.1884	6385.8	93.78	12.83	16.476	-22.1
	0.06	972.38	0.3768	6383.9	37.13	64.79	35.817	80.88
	0.06	940.82	0.3768	6384	38.38	62.68	50.316	24.56
	0.06	715.76	0.3768	6384.5	50.45	47.68	41.652	14.48
	0.06	716.96	0.3768	6384.2	50.36	47.77	47.319	0.947
	0.06	630.77	0.3768	6384.6	57.24	42.03	39.827	5.529
	0.06	672.35	0.3768	6384.6	53.7	44.8	42.083	6.454
	0.06	534.07	0.3768	6384.9	67.61	35.58	43.664	-18.5
	0.06	487.93	0.3768	6385.2	74.01	32.51	41.113	-20.9
	0.06	507.6	0.3768	6385.2	71.14	33.82	38.528	-12.2
	0.06	147.67	0.3768	6385.5	81.52	29.52	33.452	-11.8
	0.06	178.61	0.3768	6384.8	67.39	35.7	40.531	-11.9
	0.06	131.37	0.3768	6385.7	91.63	26.26	30.489	-13.9
	0.09	1464.23	0.5652	6383.7	32.88	109.7	67.476	62.63
	0.09	1273.33	0.5652	6383.9	37.81	95.43	75.979	25.6
	0.09	972.08	0.5652	6384.5	49.53	72.85	63.84	14.12
	0.09	857.51	0.5652	6384.6	56.14	64.28	66.144	-2.82
	0.09	925.59	0.5652	6384.8	52.02	69.37	69.844	-0.68
	0.09	963.26	0.5652	6384.7	49.98	72.2	68.145	5.953
	0.09	215.58	0.5652	6384.7	55.83	64.64	58.003	11.44
	0.09	166.89	0.5652	6384.8	72.12	50.04	54.228	-7.73
	0.09	138.66	0.5652	6385.4	86.81	41.57	49.774	-16.5
		1						
	0.12	317.26	0.7536	6383.8	37.93	126.8	61.901	104.9
	0.12	320.19	0.7536	6383.9	37.59	128	95.918	33.43
	0.12	271.52	0.7536	6384.3	44.33	108.5	81.941	32.45
	0.12	211.8	0.7536	6385.1	56.83	84.67	75.799	11.7
	0.12	191.64	0.7536	6385	62.81	76.61	72.797	5.234

52h(right)

Y/m	X/m	resistivity	K	U	I	K*U/I	R	((K*U/I)-R)/R*100
	0.03	633.83	0.1884	6382.9	37.97	31.67	18.004	75.9
	0.03	586.22	0.1884	6383.3	41.06	29.29	23.918	22.46
	0.03	465	0.1884	6383.7	51.76	23.24	20.005	16.15
	0.03	412.13	0.1884	6383.7	58.4	20.59	18.873	9.121
	0.03	423.98	0.1884	6383.5	56.77	21.18	19.866	6.636
	0.03	440.82	0.1884	6383.7	54.6	22.03	20.493	7.488
	0.03	416.72	0.1884	6383.9	57.76	20.82	23.49	-11.4
	0.03	276.95	0.1884	6384.4	86.91	13.84	16.946	-18.3
	0.03	303.88	0.1884	6384.1	79.21	15.18	17.629	-13.9
	0.03	291.41	0.1884	6384.5	82.6	14.56	18.993	-23.3
	0.03	310.69	0.1884	6384.3	77.47	15.53	19.26	-19.4
	0.03	297.92	0.1884	6384.5	80.8	14.89	17.588	-15.4
	0.03	129.01	0.1884	6384.8	93.3	12.89	15.698	-17.9
	0.03	160.83	0.1884	6384.1	74.83	16.07	20.773	-22.6
	0.03	127.61	0.1884	6384.8	94.32	12.75	16.476	-22.6
	0.06	931.87	0.3768	6383.1	38.74	62.08	35.817	73.34
	0.06	926.06	0.3768	6383	38.98	61.7	50.316	22.63
	0.06	704.09	0.3768	6383.3	51.27	46.91	41.652	12.63
	0.06	718.07	0.3768	6383.3	50.27	47.85	47.319	1.113
	0.06	629.6	0.3768	6383.8	57.34	41.95	39.827	5.331
	0.06	667.83	0.3768	6383.8	54.06	44.5	42.083	5.731
	0.06	529.7	0.3768	6384.1	68.16	35.29	43.664	-19.2
	0.06	484.58	0.3768	6384.3	74.51	32.29	41.113	-21.5
	0.06	506.01	0.3768	6384.3	71.35	33.72	38.528	-12.5
	0.06	146.83	0.3768	6384.5	81.97	29.35	33.452	-12.3
	0.06	177.96	0.3768	6384	67.63	35.57	40.531	-12.2
	0.06	130.68	0.3768	6384.9	92.1	26.12	30.489	-14.3
	0.09	1367.25	0.5652	6382.9	35.2	102.5	67.476	51.89
	0.09	1233.35	0.5652	6383	39.03	92.43	75.979	21.66
	0.09	950.16	0.5652	6383.5	50.66	71.22	63.84	11.56
	0.09	857.19	0.5652	6383.7	56.16	64.25	66.144	-2.87
	0.09	922.64	0.5652	6383.7	52.17	69.16	69.844	-0.98
	0.09	958.43	0.5652	6383.8	50.23	71.83	68.145	5.411
	0.09	214.31	0.5652	6383.8	56.15	64.26	58.003	10.79
	0.09	165.8	0.5652	6384.1	72.58	49.71	54.228	-8.32
	0.09	137.83	0.5652	6384.6	87.32	41.33	49.774	-17
		1						
	0.12	310.63	0.7536	6383	38.74	124.2	61.901	100.6
	0.12	319.67	0.7536	6383	37.64	127.8	95.918	33.23
	0.12	269.14	0.7536	6383.4	44.71	107.6	81.941	31.31
	0.12	211.55	0.7536	6384.3	56.89	84.57	75.799	11.57
	0.12	191.2	0.7536	6384	62.94	76.44	72.797	5.001

55h(right)

Y/m	X/m	resistivity	K	U	I	K*U/I	R	((K*U/I)-R)/R*100
	0.03	644.57	0.1884	6382.9	37.34	32.21	18.004	78.87
	0.03	618.48	0.1884	6383.3	38.91	30.91	23.918	29.23
	0.03	477.4	0.1884	6383.7	50.42	23.85	20.005	19.24
	0.03	410.57	0.1884	6383.7	58.62	20.52	18.873	8.712
	0.03	423.48	0.1884	6383.5	56.83	21.16	19.866	6.523
	0.03	431.71	0.1884	6383.8	55.75	21.57	20.493	5.272
	0.03	403.14	0.1884	6383.9	59.7	20.15	23.49	-14.2
	0.03	274.9	0.1884	6384.6	87.56	13.74	16.946	-18.9
	0.03	298.21	0.1884	6384.3	80.72	14.9	17.629	-15.5
	0.03	287.48	0.1884	6384.5	83.73	14.37	18.993	-24.4
	0.03	302.24	0.1884	6384.4	79.64	15.1	19.26	-21.6
	0.03	292.51	0.1884	6384.5	82.29	14.62	17.588	-16.9
	0.03	125.54	0.1884	6384.9	95.88	12.55	15.698	-20.1
	0.03	156.28	0.1884	6384.2	77.01	15.62	20.773	-24.8
	0.03	123.94	0.1884	6385	97.11	12.39	16.476	-24.8
	0.06	969.4	0.3768	6383.1	37.24	64.59	35.817	80.32
	0.06	974.42	0.3768	6383.1	37.05	64.92	50.316	29.02
	0.06	724.32	0.3768	6383.4	49.84	48.26	41.652	15.86
	0.06	707.91	0.3768	6383.5	51	47.16	47.319	-0.33
	0.06	627.7	0.3768	6383.7	57.52	41.82	39.827	5.001
	0.06	655.46	0.3768	6383.8	55.08	43.67	42.083	3.774
	0.06	519.84	0.3768	6384.2	69.45	34.64	43.664	-20.7
	0.06	477.61	0.3768	6384.2	75.59	31.82	41.113	-22.6
	0.06	498.29	0.3768	6384.3	72.46	33.2	38.528	-13.8
	0.06	143.43	0.3768	6384.9	83.92	28.67	33.452	-14.3
	0.06	172.35	0.3768	6384.2	69.83	34.45	40.531	-15
	0.06	127.91	0.3768	6385.1	94.11	25.56	30.489	-16.2
	0.09	1437.9	0.5652	6382.8	33.47	107.8	67.476	59.74
	0.09	1312.41	0.5652	6383	36.68	98.35	75.979	29.45
	0.09	980.41	0.5652	6383.5	49.1	73.48	63.84	15.1
	0.09	853.59	0.5652	6383.7	56.39	63.98	66.144	-3.27
	0.09	912.21	0.5652	6383.8	52.77	68.37	69.844	-2.1
	0.09	941.59	0.5652	6384	51.13	70.57	68.145	3.558
	0.09	210.42	0.5652	6384	57.2	63.08	58.003	8.756
	0.09	162.53	0.5652	6384.1	74.04	48.73	54.228	-10.1
	0.09	136.47	0.5652	6384.7	88.2	40.91	49.774	-17.8
		1						
	0.12	317.75	0.7536	6383	37.87	127	61.901	105.2
	0.12	333.14	0.7536	6383	36.12	133.2	95.918	38.84
	0.12	274.03	0.7536	6383.6	43.92	109.5	81.941	33.67
	0.12	209.19	0.7536	6384.2	57.53	83.63	75.799	10.33
	0.12	192.91	0.7536	6384.1	62.39	77.11	72.797	5.928
58h(right)								

Y/m	X/m	resistivity	K	U	I	K*U/I	R	((K*U/I)-R)/R*100
	0.03	633.06	0.1884	6383.5	38.02	31.63	18.004	75.69
	0.03	609.14	0.1884	6383.7	39.51	30.44	23.918	27.27
	0.03	471.51	0.1884	6384.3	51.05	23.56	20.005	17.78
	0.03	411.22	0.1884	6384.3	58.54	20.55	18.873	8.871
	0.03	421.97	0.1884	6384.1	57.04	21.09	19.866	6.14
	0.03	430.45	0.1884	6384.3	55.92	21.51	20.493	4.961
	0.03	401.06	0.1884	6384.6	60.02	20.04	23.49	-14.7
	0.03	272.74	0.1884	6385.1	88.26	13.63	16.946	-19.6
	0.03	295.93	0.1884	6384.9	81.34	14.79	17.629	-16.1
	0.03	285.98	0.1884	6385.2	84.18	14.29	18.993	-24.8
	0.03	300.64	0.1884	6384.9	80.07	15.02	19.26	-22
	0.03	290.85	0.1884	6385.1	82.77	14.53	17.588	-17.4
	0.03	124.51	0.1884	6385.5	96.68	12.44	15.698	-20.7
	0.03	155.71	0.1884	6384.9	77.3	15.56	20.773	-25.1
	0.03	123.02	0.1884	6385.7	97.86	12.29	16.476	-25.4
	0.06	945.29	0.3768	6383.6	38.19	62.98	35.817	75.85
	0.06	958.38	0.3768	6383.5	37.67	63.85	50.316	26.9
	0.06	713.52	0.3768	6384	50.6	47.54	41.652	14.14
	0.06	708.93	0.3768	6383.9	50.93	47.23	47.319	-0.19
	0.06	624.93	0.3768	6384.4	57.78	41.63	39.827	4.539
	0.06	653.11	0.3768	6384.4	55.28	43.52	42.083	3.409
	0.06	515.32	0.3768	6384.8	70.07	34.33	43.664	-21.4
	0.06	473.53	0.3768	6384.9	76.25	31.55	41.113	-23.3
	0.06	496.09	0.3768	6384.9	72.79	33.05	38.528	-14.2
	0.06	142.6	0.3768	6385.5	84.41	28.5	33.452	-14.8
	0.06	171.82	0.3768	6384.6	70.05	34.34	40.531	-15.3
	0.06	127.28	0.3768	6385.6	94.58	25.44	30.489	-16.6
	0.09	1390.43	0.5652	6383.4	34.62	104.2	67.476	54.45
	0.09	1279.45	0.5652	6383.5	37.62	95.9	75.979	26.23
	0.09	961.99	0.5652	6384.1	50.04	72.11	63.84	12.95
	0.09	856.85	0.5652	6384.3	56.18	64.23	66.144	-2.89
	0.09	908.27	0.5652	6384.4	53	68.08	69.844	-2.52
	0.09	939.69	0.5652	6384.6	51.23	70.44	68.145	3.366
	0.09	209.81	0.5652	6384.5	57.37	62.9	58.003	8.442
	0.09	161	0.5652	6384.7	74.76	48.27	54.228	-11
	0.09	135.56	0.5652	6385.2	88.79	40.65	49.774	-18.3
		1						
	0.12	315.15	0.7536	6383.6	38.19	126	61.901	103.5
	0.12	330.83	0.7536	6383.4	36.38	132.2	95.918	37.86
	0.12	272.05	0.7536	6384.1	44.24	108.7	81.941	32.72
	0.12	208.93	0.7536	6385	57.61	83.52	75.799	10.19
	0.12	191.69	0.7536	6384.7	62.79	76.63	72.797	5.263
70h(right)								

Y/m	X/m	resistivity	K	U	I	K*U/I	R	((K*U/I)-R)/R*100
	0.03	654.74	0.1884	6383.3	36.76	32.72	18.004	81.71
	0.03	634.41	0.1884	6383.5	37.94	31.7	23.918	32.53
	0.03	486.03	0.1884	6384	49.52	24.29	20.005	21.41
	0.03	410.02	0.1884	6384.1	58.7	20.49	18.873	8.571
	0.03	422.15	0.1884	6383.8	57.01	21.1	19.866	6.192
	0.03	430.06	0.1884	6384	55.97	21.49	20.493	4.862
	0.03	394.11	0.1884	6384.3	61.08	19.69	23.49	-16.2
	0.03	271.78	0.1884	6384.8	88.57	13.58	16.946	-19.9
	0.03	294.84	0.1884	6384.5	81.64	14.73	17.629	-16.4
	0.03	281.46	0.1884	6384.7	85.53	14.06	18.993	-26
	0.03	294.45	0.1884	6384.7	81.75	14.71	19.26	-23.6
	0.03	288.03	0.1884	6384.8	83.57	14.39	17.588	-18.2
	0.03	123.48	0.1884	6385.2	97.48	12.34	15.698	-21.4
	0.03	152.23	0.1884	6384.5	79.06	15.21	20.773	-26.8
	0.03	119.73	0.1884	6385.3	100.5	11.97	16.476	-27.4
	0.06	996.25	0.3768	6383.3	36.24	66.37	35.817	85.3
	0.06	1003.71	0.3768	6383.5	35.97	66.87	50.316	32.9
	0.06	740.54	0.3768	6383.7	48.75	49.34	41.652	18.46
	0.06	704.97	0.3768	6383.7	51.21	46.97	47.319	-0.74
	0.06	624.48	0.3768	6384.2	57.82	41.6	39.827	4.463
	0.06	654.36	0.3768	6384	55.17	43.6	42.083	3.608
	0.06	506.66	0.3768	6384.4	71.26	33.76	43.664	-22.7
	0.06	468.34	0.3768	6384.6	77.1	31.2	41.113	-24.1
	0.06	492.43	0.3768	6384.6	73.32	32.81	38.528	-14.8
	0.06	140.87	0.3768	6385.1	85.45	28.16	33.452	-15.8
	0.06	168.78	0.3768	6384.2	71.31	33.73	40.531	-16.8
	0.06	125.51	0.3768	6385.2	95.91	25.09	30.489	-17.7
	0.09	1498.34	0.5652	6383.3	32.13	112.3	67.476	66.41
	0.09	1359.39	0.5652	6383.3	35.41	101.9	75.979	34.1
	0.09	1009.99	0.5652	6383.8	47.66	75.71	63.84	18.59
	0.09	852.48	0.5652	6384.1	56.47	63.9	66.144	-3.4
	0.09	900.84	0.5652	6384.2	53.44	67.52	69.844	-3.33
	0.09	938.7	0.5652	6384.2	51.28	70.37	68.145	3.259
	0.09	209.46	0.5652	6384.2	57.46	62.8	58.003	8.267
	0.09	158.88	0.5652	6384.4	75.75	47.64	54.228	-12.2
	0.09	135.141	0.5652	6385	89.07	40.52	49.774	-18.6
	0.12	325.29	0.7536	6383.4	36.99	130	61.901	110.1
	0.12	341.82	0.7536	6383.3	35.21	136.6	95.918	42.43
	0.12	279.72	0.7536	6383.8	43.02	111.8	81.941	36.47
	0.12	207.69	0.7536	6384.5	57.95	83.03	75.799	9.535
	0.12	193.1	0.7536	6384.4	62.33	77.19	72.797	6.036

fig5-3	LNAPL Migration Depth vs. Time	Time/h	Depth/cm
		0.5	9
		1	11.6
		1.5	13
		2	14.1
		2.5	15.3
		3	16.2
		3.5	17
		4	18.3
		5	20.5
		6	22
		7	22.6
		8	23.3
		9	25.3
		11	27.4
		13	32.2
		18	40
		20	43.3
		24	45
		28	46
		30	46.1
		32	46.1
		34	46.2
		36	46.2
		48	46.3

fig6-2	Constant Water [illegible]	Time/h	Depth/cm
		0.5	1.4
	1	1	1.8
		1.5	2.5
	2	2	2.8
		3	3.7
	3	4	5.4
		5	6.8
	4	6	7.2
		7	7.4
	5	8	7.9
		13	12.4
	6	24	18.9
		28	19.7
	7	30	20.1
		34	21
	8	36	21.9

			47	22.2
			50	22.3
fig7-2	Groundwater		55	22.4
		1	62	26.4
			62.5	29.1
		2	63	30.4
			63.5	31.3
		3	64	33.1
			64.5	36.4
		4	65.5	38.7
			66	41.6
		5	66.5	43.7
			67	44.3
			67.5	44.3
		6	68.5	46
			69.5	48.3
			70.5	48.7
		7	72.5	53
			87	60

FIG5-4	normal probability plot of	x (axis)	residual distribution
		0	-1.2556
		0.5	-0.5583
		1	2.57263
		1.5	1.19878
		2	0.09786
		2.5	-0.4378
		3	-0.9021
		3.5	-1.1769
		4	-0.7323
		5	0.18469
		6	0.67692
		7	0.29523
		8	-0.1118
		9	0.58025
		11	-0.5727
		13	0.39125
		18	-0.6541
		20	0.37368
		24	-0.1022
		28	0.29303
		30	0.10333
		32	-0.2188
		34	-0.2491
		36	0.20632
		48	-0.0024

FIG5-5	rate fitting	x1 = 0	
		x2 = 55	
		n = 100	Y(x) = (4055009207296 8793*x^6)/6044629098073145873 53088 - (1273622240429 9433*x^5)/1180591620717411303 424 + (2457290778058 8495*x^4)/3689348814741910323 2 - (2831056400637
		0	5.2383
		0.55556	4.3425
		1.11111	3.60001
		1.66667	2.99281
		2.22222	2.5043
		2.77778	2.11917
		3.33333	1.82338
		3.88889	1.60409
		4.44444	1.4496
		5	1.34929
		5.55556	1.29355
		6.11111	1.27375
		6.66667	1.28215
		7.22222	1.31188
		7.77778	1.35686
		8.33333	1.41177
		8.88889	1.47201
		9.44444	1.53359
		10	1.59318
		10.5556	1.64799
		11.1111	1.69573
		11.6667	1.73463
		12.2222	1.76332

12.7778	1.78084
13.3333	1.7866
13.8889	1.78032
14.4444	1.76202
15	1.73195
15.5556	1.69061
16.1111	1.63867
16.6667	1.57698
17.2222	1.5065
17.7778	1.42832
18.3333	1.3436
18.8889	1.25355
19.4444	1.15943
20	1.06251
20.5556	0.96405
21.1111	0.86529
21.6667	0.76744
22.2222	0.67164
22.7778	0.57896
23.3333	0.4904
23.8889	0.40686
24.4444	0.32915
25	0.25795
25.5556	0.19383
26.1111	0.13726
26.6667	0.08855
27.2222	0.04791
27.7778	0.01539
32.2222	0.01487
32.7778	0.03849
33.3333	0.06456
33.8889	0.09208
34.4444	0.11998
35	0.1472
35.5556	0.17268
36.1111	0.19538
36.6667	0.2143
37.2222	0.22854
37.7778	0.23726
38.3333	0.23975
38.8889	0.23546
39.4444	0.22401
40	0.20521
40.5556	0.17914
41.1111	0.14611
41.6667	0.10678
42.2222	0.06212
42.7778	0.01349
48.8889	0.10758

FIG5-6	Time (x-axis)	1h	2h	3h	5h	10h	15h	36h	48h
	9	46.3938	48.3962	49.058	51.675	54.77	56.16	60.685	63.28
	12	36.4293	36.4486	36.976	37.318	38.87	41.16	59.825	57.94
	15	39.9276	39.8527	40.239	40.462	41.32	41.98	88.332	81.55
	18	40.7724	40.5915	40.903	40.894	43.54	44.16	69.107	67.13
	21	68.3283	71.5126	68.794	69.368	77.3	76.97	147.03	127.7
	24	43.5261	43.1176	43.68	44.031	46.47	47.23	65.444	65.58
	27	45.2358	44.5636	44.703	44.024	48.02	48.86	62.687	62.35
	30	33.3416	32.8038	32.871	32.625	35.45	36.74	40.657	40.84
	33	46.9669	48.6557	50.835	55.359	67.33	68.24	103.69	125.2
	36	40.0827	41.705	43.745	47.632	58.04	59.35	132.85	164.2
	39	42.9041	43.8158	44.596	44.785	41.25	41.34	79.029	80.26
	42	31.4709	31.1473	31.64	31.692	30.29	31.58	90.404	99.35

FIG6-3	normal probability plot of	x (axis)	residual distribution
		0	-0.5189
		0.5	0.26405
		1	0.06385
		1.5	0.17986
		2	-0.0886
		3	-0.281
		4	0.38202
		5	0.79634
		6	0.25817
		7	-0.4359
		8	-0.7891
		13	-0.0297
		24	0.58349
		28	-0.079
		30	-0.2771
		34	-0.3067
		36	0.25919
		47	-0.0291
		50	0.08274
		55	-0.0346

FIG6-4	rate fitting	
		x1 = 0
		x2 = 48

n = 100	Y(x) = (3205920157890 5445*x^4)/37778931862957161709568 - (8097013843714 07*x^3)/9223372036854775808 + (1454479653351
0	1.2018
0.48485	1.16922
0.9697	1.13806
1.45455	1.10827
1.93939	1.07979
2.42424	1.05256
2.90909	1.02652
3.39394	1.00163
3.87879	0.97783
4.36364	0.95507
4.84848	0.93329
5.33333	0.91246
5.81818	0.89251
6.30303	0.87341
6.78788	0.85511
7.27273	0.83756
7.75758	0.82071
8.24242	0.80454
8.72727	0.78899
9.21212	0.77402
9.69697	0.7596
10.1818	0.74569
10.6667	0.73224
11.1515	0.71924
11.6364	0.70663
12.1212	0.69438
12.6061	0.68248
13.0909	0.67087
13.5758	0.65953
14.0606	0.64844
14.5455	0.63757
15.0303	0.62688
15.5152	0.61636
16	0.60597

16.4849	0.5957
16.9697	0.58552
17.4546	0.57541
17.9394	0.56535
18.4242	0.55532
18.9091	0.54531
19.3939	0.5353
19.8788	0.52526
20.3636	0.5152
20.8485	0.50509
21.3333	0.49492
21.8182	0.48468
22.303	0.47436
22.7879	0.46396
23.2727	0.45346
23.7576	0.44286
24.2424	0.43215
24.7273	0.42132
25.2121	0.41038
25.697	0.39933
26.1818	0.38816
26.6667	0.37687
27.1515	0.36546
27.6364	0.35394
28.1212	0.3423
28.6061	0.33056
29.0909	0.31872
29.5758	0.30679
30.0606	0.29477
30.5455	0.28268
31.0303	0.27052
31.5152	0.25831
32	0.24605
32.4849	0.23377
32.9697	0.22147
33.4546	0.20917
33.9394	0.1969
34.4242	0.18466
34.9091	0.17248
35.3939	0.16038
35.8788	0.14838
36.3636	0.1365
36.8485	0.12476
37.3333	0.1132
37.8182	0.10183
38.303	0.09069
38.7879	0.07979
39.2727	0.06918
39.7576	0.05889
40.2424	0.04893
40.7273	0.03935

		41.2121	0.03019
		41.697	0.02147
		42.1818	0.01323
		42.6667	0.00551

FIG6-5	Time (x-axis)	1h	2h	3h	5h	10h	15h	36h	48h
	9	35.8168	46.0475	46.036	44.873	48.39	49.17	55.748	57.71
	12	50.316	65.0051	63.526	61.288	66.92	67.56	67.132	64.49
	15	41.6515	50.0939	49.321	47.469	53.49	54.01	49.732	48.58
	18	47.3194	58.4544	56.996	55.163	57.01	57.6	52.66	50.47
	21	39.8267	48.2011	47.002	45.062	46.47	46.1	43.716	43.7
	24	42.0831	49.7551	49.163	47.443	49.01	48.62	46.37	46.87
	27	43.6645	51.6791	50.399	47.267	48.75	48.36	39.202	37.47
	30	41.1134	47.4696	46.784	44.678	45.39	45.13	36.463	34.28
	33	38.5277	44.9434	44.402	42.576	43.91	43.62	35.994	35.08
	36	33.4517	41.4038	40.442	39.065	40.22	39.66	32.356	31.04
	39	40.5307	50.7508	49.742	48.444	50.18	49.61	39.607	37.81
	42	30.489	36.45	35.733	34.441	35.45	35.14	28.397	27.38

FIG7-3	normal probability plot of	x (axis)	residual distribution
		0	-0.1267
		0.5	-0.3546
		1	0.23829
		1.5	-0.2386
		2	2.10967
		2.5	-0.0361
		3	-1.1082
		3.5	-1.2511
		4	0.17893
		5	-0.9196
		5.5	0.48908
		6	1.24665
		6.5	0.65124
		7	-0.4058
		8	-0.4685
		9	0.38444
		10	-0.5785
		12	0.18965
		26.5	-0.0005

FIG7-4	rate fitting

x1 = 0
x2 = 30

n = 100	Y(x) = -(11048123960626125*x^4)/737869762948382064 64 + (2307687683621065*x^3)/288230376151711744 -
0	3.1834
0.30303	3.30573
0.60606	3.40576
0.90909	3.4848
1.21212	3.54411
1.51515	3.5849
1.81818	3.60839
2.12121	3.61575
2.42424	3.60811
2.72727	3.58658
3.0303	3.55224
3.33333	3.50615
3.63636	3.44931
3.93939	3.38273
4.24242	3.30735
4.54545	3.22411
4.84848	3.13389
5.15152	3.03758
5.45455	2.936
5.75758	2.82996
6.06061	2.72024
6.36364	2.60758
6.66667	2.4927
6.9697	2.37629
7.27273	2.259
7.57576	2.14145
7.87879	2.02424
8.18182	1.90793
8.48485	1.79306
8.78788	1.68012
9.09091	1.5696
9.39394	1.46194
9.69697	1.35755
10	1.2568
10.303	1.16006

10.6061	1.06764
10.9091	0.97984
11.2121	0.89691
11.5152	0.81909
11.8182	0.74658
12.1212	0.67954
12.4242	0.61813
12.7273	0.56244
13.0303	0.51255
13.3333	0.46853
13.6364	0.43037
13.9394	0.39808
14.2424	0.37161
14.5455	0.35088
14.8485	0.33581
15.1515	0.32624
15.4546	0.32202
15.7576	0.32296
16.0606	0.32883
16.3636	0.33938
16.6667	0.35433
16.9697	0.37335
17.2727	0.3961
17.5758	0.42222
17.8788	0.45128
18.1818	0.48287
18.4849	0.5165
18.7879	0.55169
19.0909	0.58791
19.3939	0.62459
19.697	0.66116
20	0.697
20.303	0.73145
20.6061	0.76384
20.9091	0.79347
21.2121	0.81958
21.5152	0.84142
21.8182	0.85817
22.1212	0.86903
22.4242	0.87311
22.7273	0.86954
23.0303	0.85738
23.3333	0.8357
23.6364	0.80351
23.9394	0.7598
24.2424	0.70352
24.5455	0.6336
24.8485	0.54895
25.1515	0.44842
25.4546	0.33086
25.7576	0.19508

		26.0606	0.03983					
FIG7-5	Time (x-axis)	48h	49h	50h	52h	55h	58h	70h
	9	57.7132	61.5934	60.589	63.379	63.92	66.15	64.256
	12	64.4867	62.59	67.301	69.797	72.25	80.21	76.986
	15	48.5762	88.7964	82.803	83.432	84.82	89.06	89.302
	18	50.4715	71.1022	74.354	83.541	84.24	82.12	84.037
	21	43.6985	123.259	121.83	124.47	125.5	125.4	125.79
	24	46.8744	68.512	71.59	75.19	80.67	86.4	86.954
	27	37.4733	62.7281	63.86	69.502	75.51	80.61	82.203
	30	34.2794	40.4085	40.321	40.659	40.93	41.39	41.86
	33	35.0751	165.535	162.22	159.09	159.1	150.9	151.6
	36	31.0362	162.346	157.86	149.61	149.4	144.9	145.89
	39	37.8067	86.681	91.008	106.76	106.2	104.4	105.9
	42	27.3844	114.745	121.18	124.2	126	134.4	129.43
